# Abnormal expression of *bHLH3* disrupts a flavonoid homeostasis network, causing differences in pigment composition among mulberry fruits

**DOI:** 10.1038/s41438-020-0302-8

**Published:** 2020-06-01

**Authors:** Han Li, Zhen Yang, Qiwei Zeng, Shibo Wang, Yiwei Luo, Yan Huang, Youchao Xin, Ningjia He

**Affiliations:** State Key Laboratory of Silkworm Genome Biology, Southwest University, Beibei, 400715 Chongqing, P.R. China

**Keywords:** Secondary metabolism, Plant molecular biology

## Abstract

Mulberry fruits with high concentrations of anthocyanins are favored by consumers because of their good taste, bright color, and high nutritional value. However, neither the regulatory mechanism controlling flavonoid biosynthesis in mulberry nor the molecular basis of different mulberry fruit colors is fully understood. Here, we report that a flavonoid homeostasis network comprising activation and feedback regulation mechanisms determines mulberry fruit color. In vitro and in vivo assays showed that MYBA-bHLH3-TTG1 regulates the biosynthesis of anthocyanins, while TT2L1 and TT2L2 work with bHLH3 or GL3 and form a MYB-bHLH-WD40 (MBW) complex with TTG1 to regulate proanthocyanidin (PA) synthesis. Functional and expression analyses showed that *bHLH3* is a key regulator of the regulatory network controlling mulberry fruit coloration and that *MYB4* is activated by MBW complexes and participates in negative feedback control of the regulatory network to balance the accumulation of anthocyanins and proanthocyanidins. Our research demonstrates that the interaction between bHLH3 and MYB4 in the homeostasis regulatory network ensures that the fruits accumulate desirable flavonoids and that this network is stable in pigment-rich mulberry fruits. However, the abnormal expression of *bHLH3* disrupts the balance of the network and redirects flavonoid metabolic flux in pale-colored fruits, resulting in differences in the levels and proportions of anthocyanins, flavones, and flavonols among differently colored mulberry fruits (red, yellow, and white). The results of our study reveal the molecular basis of the diversity of mulberry fruit colors.

## Introduction

Fruits are an essential part of people’s daily diet and provide nutrients such as vitamins, minerals, and dietary fiber. The color, flavor, and nutritional value of fruits are the main factors for consumer acceptance^1^. In plant reproductive organs, flavonoids/anthocyanins, carotenoids, and betalains are the three main pigments for coloration^2^. Depending on their structures, flavonoids can be divided into more than a dozen groups, including flavonols, chalcones, anthocyanins, and flavones^3^. Anthocyanins, which are water-soluble compounds that are widely found in the plant kingdom, are the most important pigments in fruits and flowers and confer red-to-blue coloration to plant tissues. The types and quantities of anthocyanins are major factors affecting fruit quality^2^. Carotenoids, the lipid-soluble isoprenoid compounds that are widely found in seed plants, are indispensable components of photosystems and have various colors ranging from yellow to red^4^. In contrast to carotenoids and anthocyanins, betalains, which are water-soluble compounds with a yellow-to-red color range, are found only in the order Caryophyllalles^5^. All of these pigments also have a variety of biological activities. Flavonoids/anthocyanins have high nutritional value because of their health effects on the human body^6^. Anthocyanins have a broad spectrum of medicinal effects, such as protection against liver injury, reduction in blood pressure, improvement of eyesight, strong anti-inflammatory activity, and anticancer properties^7^. Because these pigments contribute to the health-promoting quality of fruits, fruits that are rich in anthocyanins are more likely to be favored by consumers than those that are not^8^.

The flavonoid pathway is mainly regulated by MYB–bHLH–WD40 (MBW) transcription complexes, which consist of R2R3-myeloblastosis (MYB), basic helix-loop-helix (bHLH), and WD40 proteins^9^. In *Arabidopsis*, anthocyanin synthesis is controlled by different groups of MBW transcription complexes, which are composed of four subgroup 6 MYBs (AtPAP1, AtPAP2, AtMYB113, AtMYB114), three subgroup IIIf bHLHs (AtTT8, AtGL3, AtEGL3), and a TTG1 protein^10–14^. The specific accumulation of proanthocyanidins (PAs) also involves at least three MBW complexes, namely, AtTT2–AtEGL3–AtTTG1, AtTT2–AtGL3–AtTTG1, and AtTT2–AtTT8–AtTTG1, which have partially overlapping functions^12,15–18^. Similarly, anthocyanin synthesis in petunia petal cells is regulated by an MBW complex comprising the subgroup 6 MYB PhAN4 or PhAN2, the subgroup IIIf bHLH PhAN1 or PhJAF13, and the WD40 regulator PhAN11^19–21^. In contrast, subgroup 7 MYBs, such as *Arabidopsis* AtMYB111, AtMYB12, and AtMYB11 and grapevine VvMYBF1, regulate the flavonol pathway independently of any WD40 and bHLH partners^22–24^. In these cases, activator-type MYBs determine which metabolic pathway is regulated, and pleiotropic bHLHs (e.g., *Arabidopsis* AtGL3 and AtEGL3, which play a vital role in anthocyanin synthesis and the formation of root hairs and trichomes) interact with specific MYBs to regulate downstream biosynthetic genes^25^. Any locus mutation or abnormal expression of *MYB* and *bHLH* genes affects flavonoid biosynthesis, resulting in changes in plant tissue pigmentation. Several studies have demonstrated that the expression of *AtTT8* and *MtTT8* in *Arabidopsis* and *Medicago*, respectively, is highly correlated with the pigment content in the seed coat^15,26^. The *AtTT8* mutant has a low PA content in the pale-yellow seed coat, and the *MtTT8* mutant seeds have low PA and anthocyanin levels, exhibiting a transparent-testa phenotype. In apple, *MdbHLH3* expression is induced at low temperature, and then, MdbHLH3 directly regulates the expression of *MdMYB1*. MdbHLH3 and MdMYB1 then work together to activate anthocyanin biosynthesis. This suggests that the upregulation of *MdMYB1* mediated by MdbHLH3 amplifies the regulatory signal for anthocyanin biosynthesis^27^.

Flavonoids as well as lignin, which is derived from phenylalanine, can also be regulated by repressor-type MYBs, including R2R3-MYB and R3-MYB factors^28^. The R2R3-MYB repressors have two repeats in the DNA-binding domain and include *Arabidopsis* AtMYB4, petunia PhMYB27, and strawberry FaMYB1^29^. The R3-MYB repressors have only a single repeat in the DNA-binding domain and include petunia PhMYBx, *Arabidopsis* AtTRY, and AtMYBL2^29^. Both types of repressors have been shown to disrupt the interaction between the MYB activator and bHLH by interacting with bHLH cofactors, resulting in a passive repression function^28^.

Mulberry, belonging to the genus *Morus* in the family Moraceae, is an essential food crop for the domesticated silkworm. Mulberry fruits are also popular in Asia because of their good taste, bright colors, and high nutritional value^30^. Similar to apples, grapes, and pears, mulberry fruits have various colors ranging from white to yellow, red, and purple. Previous studies have shown that anthocyanins are abundant in purple mulberry fruits but not in pale (yellow or white) fruits^31,32^. The genes involved in the biosynthesis of anthocyanins in mulberry plants have been identified and cloned, and the transcriptional levels of *ANS*, *F3*’*H1*, *F3H1*, *CHI*, and *CHS1* have been shown to be correlated with anthocyanin concentrations during fruit ripening^32^. However, neither the regulation of the flavonoid pathway in mulberry nor the molecular basis for the different mulberry fruit colors is fully understood.

In the present study, we conducted metabolomic, transcriptomic, in vitro, and in vivo analyses of differently colored mulberry fruits. Eight regulatory factors participating in the regulation of the flavonoid pathway were identified: MYBA, TT2L1, TT2L2, bHLH3, GL3, TTG1, MYBF, and MYB4. Functional and expression analyses showed that a flavonoid homeostasis network based on the interaction between bHLH3 and MYB4 is stable in pigment-rich mulberry fruits: bHLH3 is an indispensable factor that activates anthocyanin biosynthesis, while MYB4, a repressor activated by the transcription complex that includes bHLH3, prevents the formation of the MBW transcriptional complex through competitive binding with bHLH3 to prevent the overaccumulation of anthocyanins. The results of our study demonstrate that the abnormal expression of *bHLH3* in pale-colored fruits disrupts the homeostasis network and redirects flavonoid metabolic flux, resulting in differences in pigment composition among mulberry fruits. All these findings reveal the molecular basis for the different fruit colors of mulberry.

## Results

### Differences in flavonoid content and composition are responsible for color differences among mulberry fruits

**Fig. 1:**
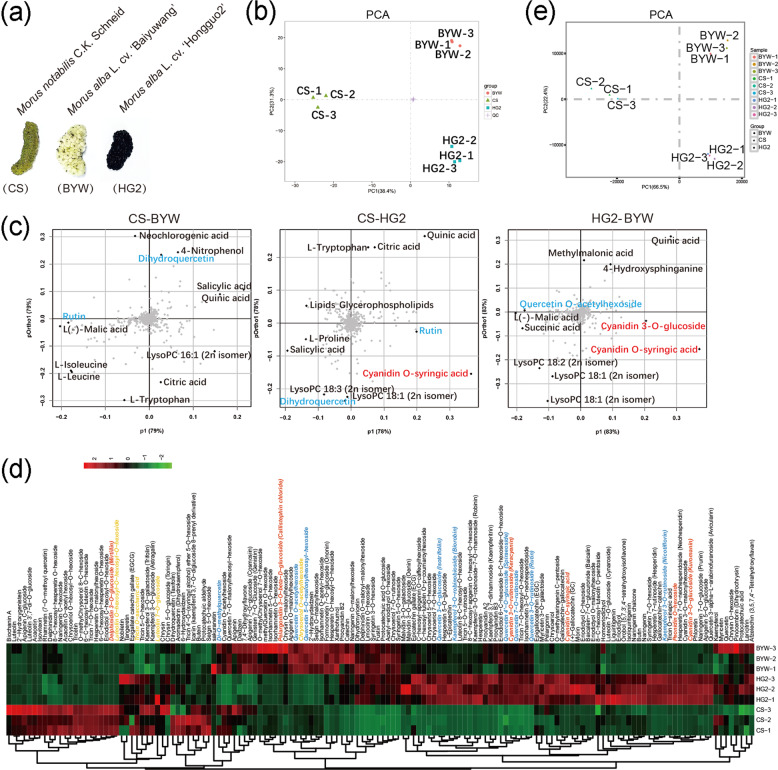
Metabolome and transcriptome analyses of differently colored mulberry fruits. **a** Three representative cultivars with typical colored fruits. **b** PCA score plot of metabolite profiles of CS (yellow), BYW (white), and HG2 (purple) mulberry fruits. **c** OPLS-DA loading plots for different mulberry cultivars. **d** Hierarchical clustering of flavonoid metabolites in CS, BYW, and HG2 fruits. Intensity values were adjusted by log transformation and then normalized. Flavones, flavonols, and anthocyanins with significant differences in abundance among differently colored fruits are indicated in yellow, blue, and red text, respectively. **e** PCA score plot of transcript profiles from CS, BYW, and HG2 fruits

Three representative cultivars with typical colored fruits, namely, *Morus notabilis* C.K. Schneid (CS), *Morus alba* L. cv. Hongguo2 (HG2), and *M. alba* L. cv. Baiyuwang (BYW), were selected for this study. The fruits of HG2 are deep purple and rich in pigments, while those of CS are yellow and those of BYW are white (Fig. 1a). LC–ESI–MS/MS-based metabolomics was performed on the fruits, and the data generated were then used for principal component analysis (PCA). The three groups (CS, HG2, and BYW) were clearly separated on the PC1 × PC2 score plot (Fig. 1b). To identify the differentially accumulated metabolites associated with the color differences among the fruits, score plots of orthogonal partial least-squares discriminant analysis (OPLS-DA) were generated to analyze the differences between pairs of the samples (Fig. S1). The metabolites with loadings that were distant from the origin on the OPLS-DA-loading plots were inferred to make the greatest contribution to class separation. Organic acids, lipids, and flavonols were major contributors to the separation among the three mulberry fruits. Anthocyanins were significant contributors to the separation between CS and HG2 and between HG2 and BYW (Fig. 1c). Overall, there were 103 compounds with significantly different levels among the three cultivars, and one-third of these compounds were flavonoids (Table S1–4). A heatmap cluster analysis was conducted to illustrate the differences in flavonoid content among the three cultivars. This analysis showed that purple fruits (HG2) contain the highest levels of anthocyanins, while yellow (CS) and white (BYW) fruits tend to accumulate flavones and flavonols, respectively (Fig. 1d).

Next, we compared the fruit transcriptomes of the three cultivars. *De novo* transcriptome assembly was carried out to construct transcripts. A total of 44–56 million clean reads were obtained after data cleanup and stringent quality checks, and the longest transcripts were considered unigenes (Table S5). CS, HG2, and BYW were clearly separated into three groups in the PCA analysis (Fig. 1e). The differentially expressed genes (DEGs) among the three cultivars were identified and then mapped to the KEGG database (Fig. S2a, b). The top 20 enriched pathways in each cultivar are listed in Fig. S2b. The pathways of phenylalanine metabolism, flavonoid biosynthesis, and phenylpropanoid biosynthesis, all of which are involved in anthocyanin synthesis, were significantly strengthened in the fruits of HG2, while the flavonol pathway was significantly strengthened in the fruits of BYW compared with those of CS.

### Combined metabolomic and transcriptomic analyses reveal redirection of metabolic flux in the flavonoid pathway in mulberry

**Fig. 2:**
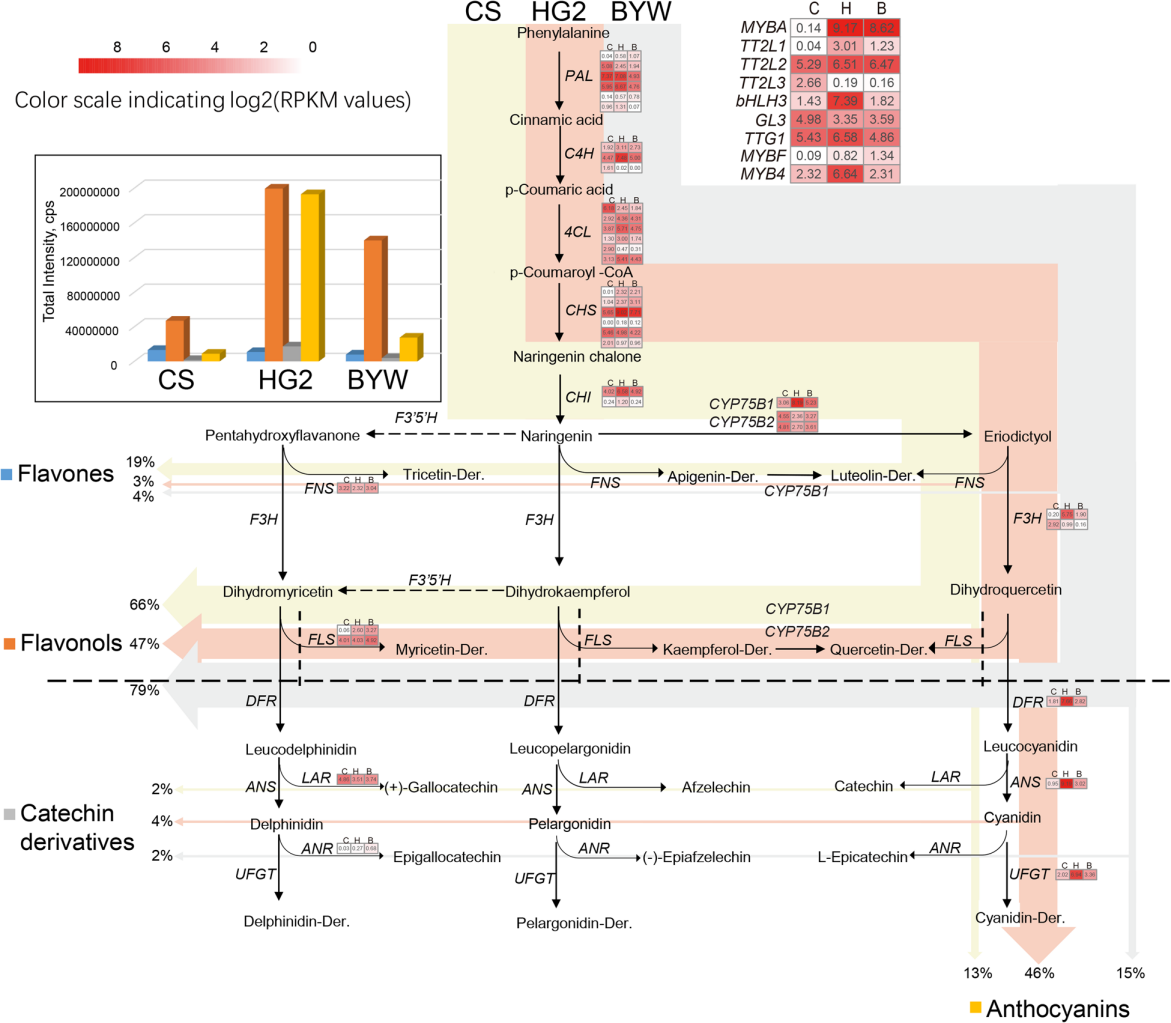
Flavonoid biosynthesis pathway in mulberry fruits. C, CS; H, HG2; B, BYW. Each colored cell represents the average log_2_(RPKM) value of each pathway gene according to the color scale. Dotted arrows indicate unidentified enzymes. Total flavonoids (flavones, flavonols, catechin derivatives, and anthocyanins) in each cultivar were set to 100% to define the relative proportion of each product

Genes involved in flavonoid synthesis were identified based on the unigenes above and the *M. notabilis* genome (Table S6). Although our metabolome analyses have shown that mulberry contains delphinidin derivatives (Del), we did not find any *F3*′*5*′*H* homologs belonging to the CYP75A subfamily that encode enzymes for Del biosynthesis (Fig. S3). This result suggested that there may be another member of the CYP75B subfamily in mulberry with catalytic activities at the 3′ and 5′ positions of the flavonoid B-ring, such as some specific *F3*′*5*′*H*s within the CYP75B subfamily in members of Asteraceae^33^. To test this idea, we constructed a yeast strain heterologously expressing *CYP75B1* or *CYP75B2* with a mulberry gene encoding a P450 reductase. However, the products of both *CYP75B* genes showed typical catalytic activities at the 3′ position and converted naringenin and kaempferol to eriodictyol and quercetin, respectively (Fig. S4b, d). Notably, the *CYP75B* gene products could not hydroxylate the 3′ position of dihydrokaempferol, and only the *CYP75B1* gene product could convert apigenin to luteolin (Fig. S4a, c). On the basis of these findings, we rearranged the flavonoid biosynthesis pathway of mulberry fruits as shown in Fig. 2.

The expression of the late (LBG) anthocyanin biosynthesis genes (*UFGT*, *ANS*, *DFR*, and *CYP75B1*) in fruits was much lower in CS and BYW than in HG2 (Fig. 2). Correspondingly, the anthocyanin content in fruits was much lower in CS and BYW than in HG2. Anthocyanins accounted for 46% of the flavonoids in HG2 but only 13–15% of the flavonoids in CS and BYW. *DFR* encodes an enzyme that is crucial at a late stage of anthocyanin formation, and the first branch upstream of this gene is the flavonol biosynthetic pathway. The ratio of flavonols to flavonoids was higher in CS and BYW than in HG2. Because of the low transcript levels of flavonoid structural genes in CS and BYW, the levels of flavonols, anthocyanins, catechins, and their derivatives were lower in CS and BYW than in HG2. However, CS had the highest concentration of flavones. The proportion of flavones in the total flavonoids was much higher in CS than in HG2 and BYW. The transcript levels of two genes, namely, *FLS* and *FNS*, were correlated with high flavonol and high flavone levels, respectively. The highest expression of *FLS* was observed in BYW, which also had the highest proportion of flavonols in the total flavonoids among the three cultivars. The lowest expression of *FLS* was observed in CS, indicating that the carbon flux to flavonols is limited in CS relative to that in BYW. This would cause some of the carbon flux to be diverted to upstream branches. Flavone biosynthesis, controlled by *FNS*, is upstream of the flavonol synthesis pathway. The highest expression of *FNS* in CS caused the carbon flux to flow to the flavone branch. This explains why CS had the highest proportion of flavones among the three mulberry resources.

### Transcription factors involved in the flavonoid pathway in mulberry fruits and their transcription patterns during fruit pigmentation

**Fig. 3:**
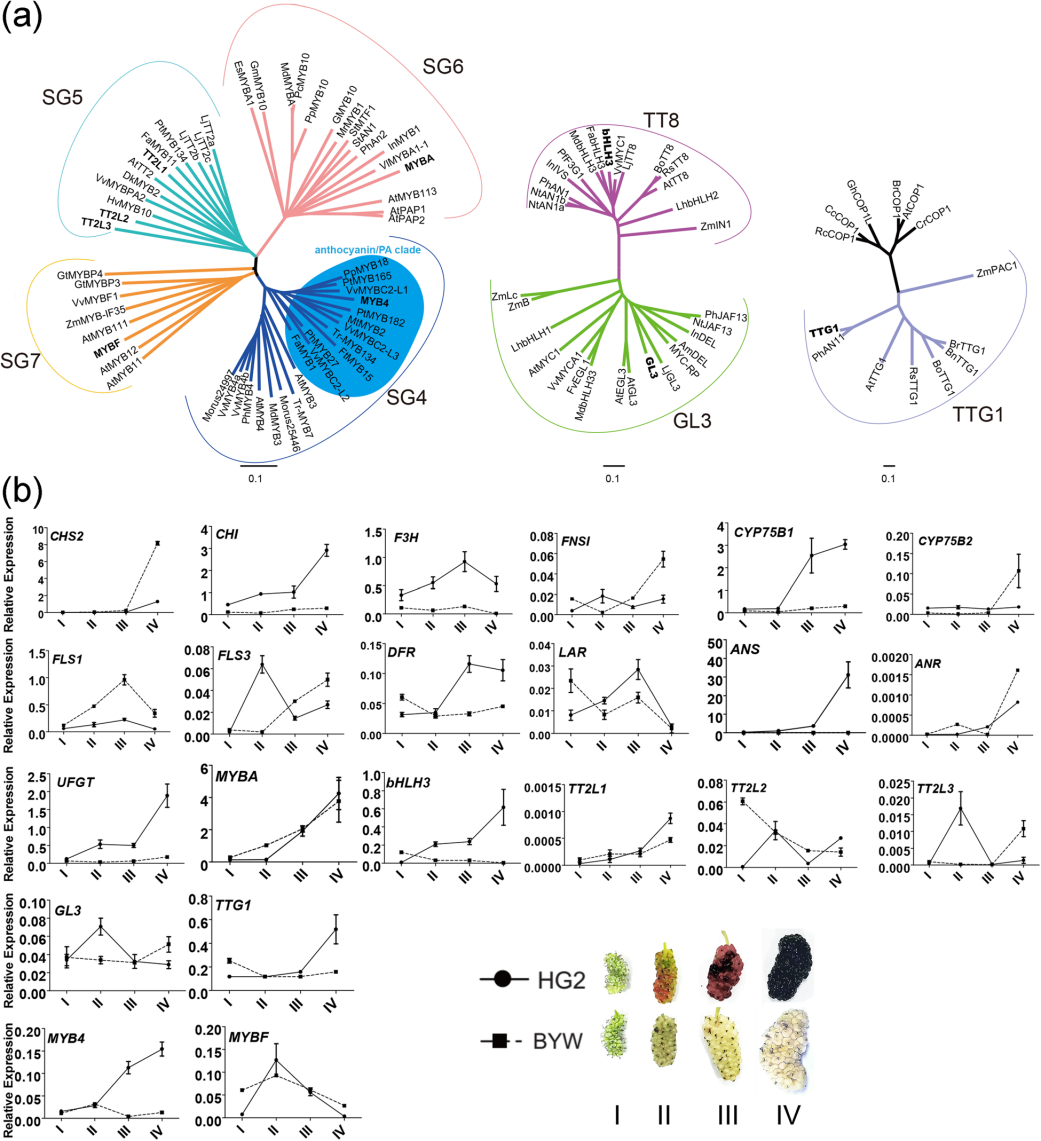
Transcription factors (TFs) related to flavonoid biosynthesis in mulberry fruits and their gene expression patterns during fruit pigmentation. **a** Phylogenetic analysis of candidate TFs in mulberry and other species. **b** Transcription profiles of flavonoid biosynthesis-related genes in mulberry fruits

Eight genes related to the regulation of the flavonoid pathway were identified (Fig. 3a). Sequence and phylogenetic analyses revealed that MYBA and MYBF show high similarity to AtMYB113 and AtMYB111, respectively; TT2L1, TT2L2, and TT2L3 are in the subgroup 5 family; two bHLH proteins, namely, bHLH3 and GL3, are in subgroup IIIf (bHLH3 in the TT8 clade and GL3 in the GL3 clade); and TTG1 is a homolog protein of AtTTG1 (Figs. 3a, S5). The products of all eight genes were localized in the nucleus (Fig. S6a).

We conducted qRT-PCR analyses to confirm the expression patterns of flavonoid synthesis-related genes in mulberry fruits. Comparison of HG2 and BYW showed that all anthocyanin biosynthesis genes except for *CHS2* were highly expressed in the purple fruits of HG2 in the final stages of fruit ripening, while their expression was almost undetectable in the pale-colored fruits of BYW and during the early stages of fruit development (Fig. 3b). The transcript level of *bHLH3* was highly correlated with those of anthocyanin-related genes, while the expression of *MYBA* showed no difference between HG2 and BYW. *MYBF* and two *FLS* genes showed the highest transcript levels at the middle stage of fruit ripening. In contrast, *ANR* showed the highest transcript level at the final stage of fruit ripening in both cultivars, while the transcript level of *LAR* fluctuated during fruit ripening. In HG2, the transcription patterns of *GL3*, *TT2L2*, and *TT2L3* were similar to that of *LAR*.

### Interaction between different regulators in MBW complexes

**Fig. 4:**
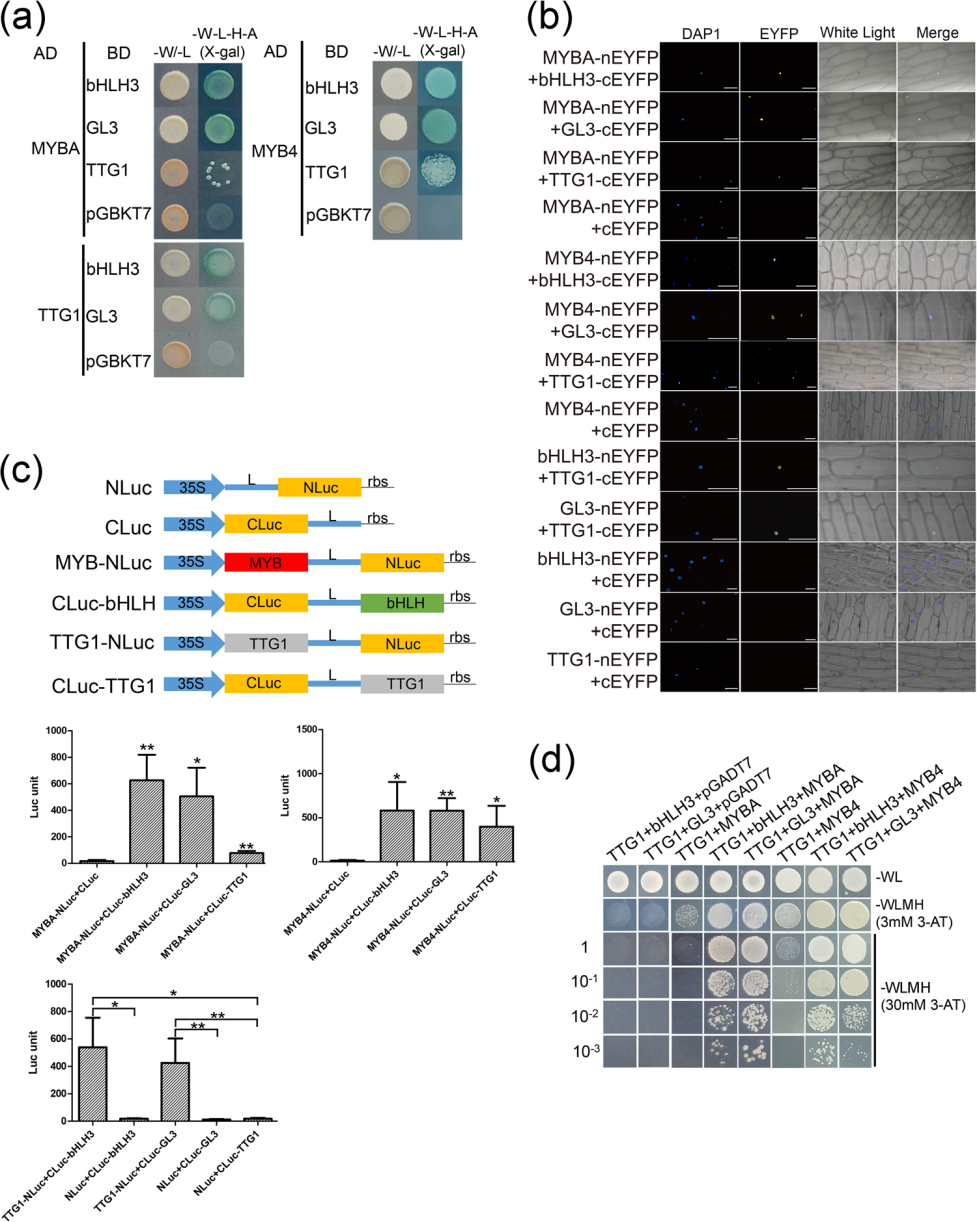
Validation of interaction between different regulators in MYB–bHLH–WD40 complexes. **a** Yeast two-hybrid assays. -W/-L: SD-Trp-Leu medium; -W-L-H-A(X-gal): SD-Trp-Leu-His-Ade+(X-a-Gal) medium. **b** Bimolecular fluorescence complementation assays. Scale bars are 100 μm. **c** Split luciferase complementation assays in *Nicotiana benthamiana* leaves. L: Gly/Ser linker, rbs: transcription terminator. The data represent averages of four experiments. Asterisks (*) indicate the statistical significance of the difference between the experimental and the control groups. (Student’s *t* test, *P < 0.05, **P < 0.01). **d** Yeast three-hybrid assays. *MYBA* and *MYB4* were inserted into the vector pGADT7. *TTG1* was recombined into pBridge at MCS I; *bHLH3* or *GL3* was recombined into pBridge at MCS II. Interactions were analyzed on SC-Leu-Trp-Met-His medium containing the indicated concentrations of 3-AT (0 and 30 mM). Transformed yeast cells were diluted 10-fold, 100-fold, and 1000-fold on the medium

In yeast two-hybrid (Y2H) and bimolecular fluorescence complementation (BiFC) assays, both bHLH3 and GL3 interacted with TTG1 and three MYB proteins (MYBA, TT2L1, and TT2L2) but not with MYBF or TT2L3 (Figs. 4a, b, S7a, b). Among the five MYB transcription factors, only MYBA was able to interact with TTG1 (Fig. 4a, b). The results of the split luciferase complementation assays in *Nicotiana benthamiana* supported these findings. The intense luciferase activity was restored when the CLuc-bHLH3 or CLuc-GL3 fusion protein was coexpressed with MYBA-NLuc, TT2L1-NLuc, or TT2L2-NLuc (Figs. 4c, S7c). The same signals were detected when TTG1-NLuc was coexpressed with CLuc-bHLH3 or CLuc-GL3, whereas no signals were detected in tissues harboring control constructs (Fig. 4c). In addition, the results of yeast three-hybrid assays showed that MYB (MYBA, TT2L1, and TT2L2), bHLH (bHLH3 and GL3), and TTG1 could form ternary complexes. All yeast strains expressing MBW proteins grew on high concentrations of 3-amino-1,2,4-triazole, suggesting equal interaction strength within the complexes (Figs. 4d, S7d).

### Functional analysis of transcription factors involved in the regulation of the flavonoid pathway

**Fig. 5:**
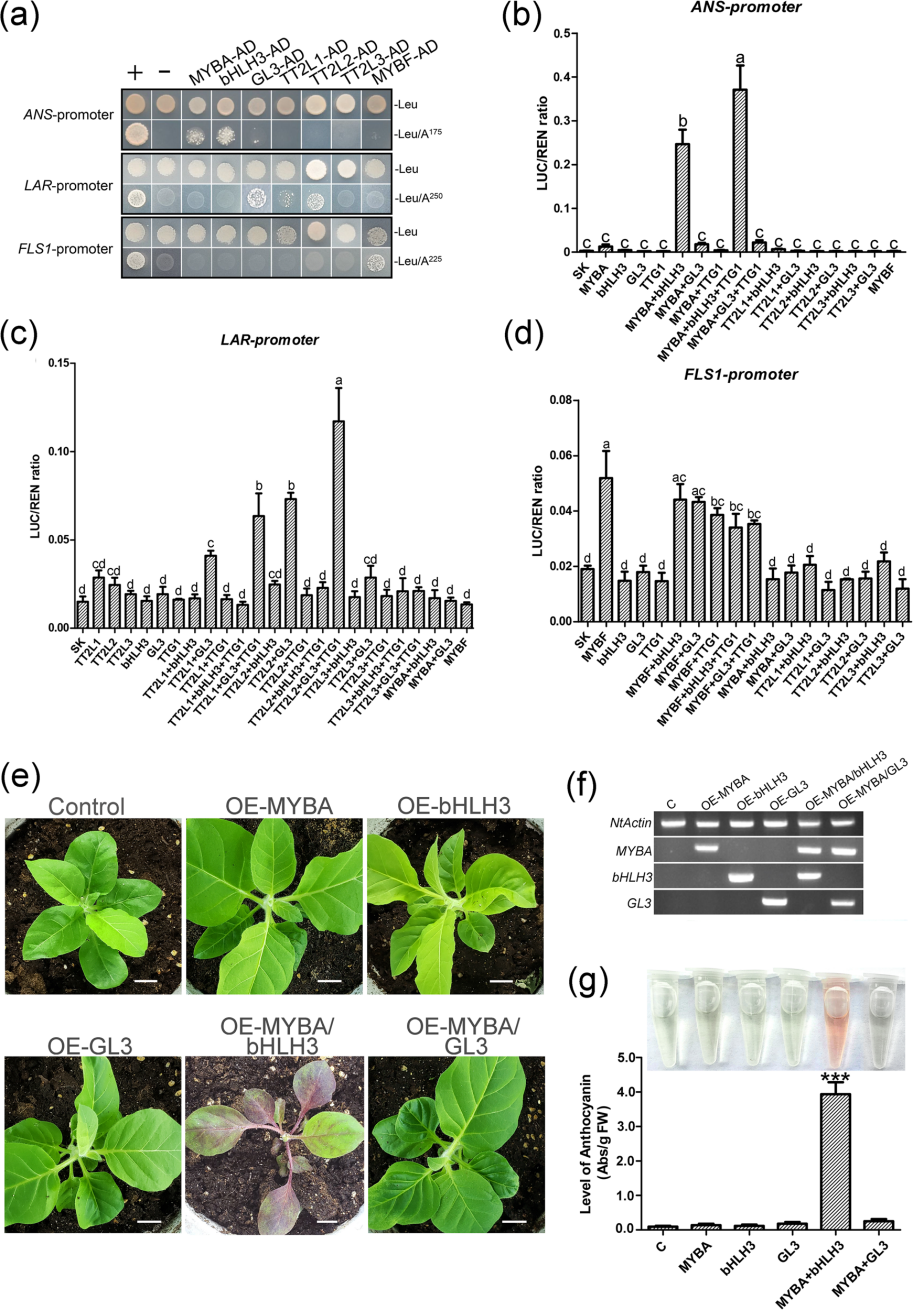
Functional analysis of regulators involved in the regulation of flavonoid biosynthesis. **a** Yeast one-hybrid assays. –Leu: SD-Leu medium. Binding was screened on SD-Leu medium in the presence of AbA^175^, AbA^250^, and AbA^225^. **b–d** Dual-luciferase reporter analysis in tobacco leaves. SK, empty vector. The data represent averages of four experiments. Significant differences between treatments were determined using one-way ANOVA (*P* < 0.05). **e** Phenotypes of transgenic tobacco harboring *MYBA*, *bHLH3*, *GL3*, *MYBA/bHLH3*, and *MYBA/GL3*. Scale bars are 1 cm. **f** The positive transgenic lines were determined by semiquantitative RT-PCR. *NtActin* served as an internal standard. **g** Total anthocyanin levels in leaves of transgenic tobacco plants. Asterisks (*) indicate the statistical significance of difference between the experimental and the control groups (Student’s *t* test, ***P < 0.001)

Yeast one-hybrid (Y1H) assays were carried out to identify the specific binding of the candidate MYBs and bHLHs to the promoters of *ANS*, *LAR*, and *FLS1*. MYBA and bHLH3 directly bound to the *ANS* promoter, and TT2L1, TT2L2, and GL3 interacted with the *LAR* promoter. Only MYBF bound to the *FLS1* promoter (Fig. 5a). Dual luciferase reporter assays in tobacco leaves were performed to confirm these results. As shown in Fig. 5b, MYBA had weak activity on the *ANS* promoter but strong activity when interacting with bHLH3. The highest *ANS* promoter activity was with the combination of MYBA, bHLH3, and TTG1. Similarly, TT2L1 and TT2L2 had high activity on the *LAR* promoter when interacting with GL3 and the highest activity when TTG1 was added (Fig. 5c). In contrast, MYBF affected the promoter activity of *FLS1* without bHLH3 or GL3 (Fig. 5d). Transformed tobacco lines overexpressing these candidate TFs were cultured to verify their functions (Figs. 5e–g, S8). Compared with the control plants harboring the empty vector, the lines overexpressing *MYBA* and *bHLH3* accumulated high concentrations of anthocyanins throughout the plant, while plants expressing other TFs had green foliage. Overexpression of *TT2L1*, *TT2L2*, and *MYBF* in tobacco reduced the anthocyanin content in flowers. Compared with the control plants, the plants transformed with *TT2L1* or *TT2L2* accumulated high levels of PAs, while overexpression of *MYBF* in tobacco increased the flavonol content (Fig. S8a–g).

### bHLH3 is indispensable for transcriptional regulation of flavonoid biosynthesis

**Fig. 6:**
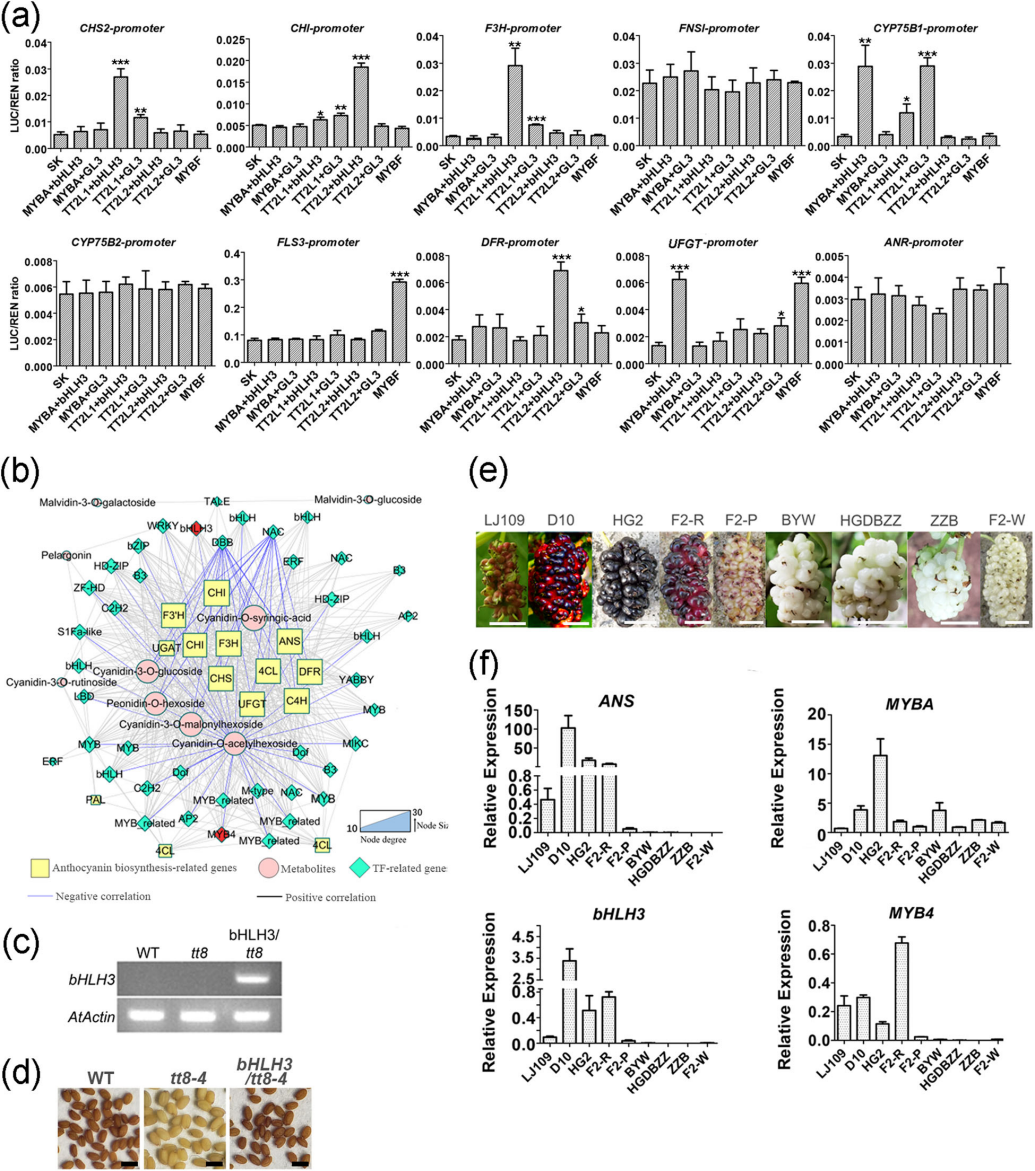
bHLH3 is an indispensable factor that activates flavonoid biosynthesis. **a** Dual-luciferase reporter assay showing that activation of the promoters of *CHS2*, *CHI*, *F3H*, *CYP75B1*, *DFR*, and *UFGT* requires participation of *bHLH3*. the data represent averages of four experiments. Asterisks (*) indicate the statistical significance of the difference between the experimental and the control groups. (Student’s *t* test, *P < 0.05, **P < 0.01, ***P < 0.001). **b** Connection network among transcription factors, anthocyanin pathway genes, and anthocyanins. **c** The positive transgenic lines were determined by semiquantitative RT-PCR. *AtActin* served as an internal standard. **d** Restored seed pigmentation in the *Arabidopsis tt8* mutant overexpressing *bHLH3*. WT, wild-type; *tt8-4,* *Arabidopsis tt8* mutant; *bHLH3*/*tt8*, T2 seed of *bHLH3-*transgenic plant. Scale bars are 1 mm. **e** Phenotypes of fruits of different mulberry cultivars. F2-R, F2-P, and F2-W are components of the F2 population derived from LJ109 and ZZB. Scale bars are 1 cm. **f** qRT-PCR analysis of *ANS*, *MYBA*, *bHLH3*, and *MYB4* expression levels in ripe fruits of different mulberry cultivars

We further investigated the effects of the candidate TFs on the promoter activity of flavonoid pathway genes, namely, *CHS2*, *CHI*, *F3H*, *FNSI*, *CYP75B1*, *CYP75B2*, *FLS3*, *DFR*, *UFGT*, and *ANR* (Fig. 6a). Interestingly, the two TT2-type TFs (TT2L1 and TT2L2) combined with bHLH3 to activate the promoters of *CHS2*, *F3H*, *CYP75B1*, *CHI*, and *DFR*. MYBA also combined with bHLH3 to activate the promoters of *CYP75B1* and *UFGT*. Notably, the participation of bHLH3 was required for the activation of most promoters, including those of *CHS2*, *CHI*, *F3H*, *CYP75B1*, *DFR*, and *UFGT*. Next, nine anthocyanin compounds and 55 transcripts were organized into a connection network (Fig. 6b). Although MYBA combined with bHLH3 to activate the promoter of *ANS* and produce excess anthocyanins in tobacco, only *bHLH3* was highly connected with anthocyanin compounds and anthocyanin pathway genes in the connection network. Additionally, our transcriptome data also showed that the gene products of *MYBA* and *bHLH3* did not differ between purple fruits and pale-colored fruits (Fig. S5a, b). Overexpression of *bHLH3*, which is a homolog of *Arabidopsis TT8*, in the *Arabidopsis tt8* mutant restored the brown color of the seed coat to the wild-type phenotype (Fig. 6c, d).

The ripe fruits of six mulberry cultivars were selected to further analyze the correlation between *bHLH3* transcription and fruit pigmentation (Fig. 6e, f). There were high transcript levels of *ANS* in pigment-rich fruits of HG2, LJ109, and D10 but undetectable or low transcript levels in the white fruits of BYW, HGDBZZ, and ZZB. The expression pattern of *bHLH3* was the same as that of *ANS*, but there was no significant difference in the transcription of *MYBA* between LJ109 and HGDBZZ or between D10 and BYW. The fruits of the F2 population derived from LJ109 and ZZB could be divided into three distinct phenotypes, namely, alternating red and white fruits (F2-P), red fruits (F2-R), and white fruits (F2-W). The expression characteristics of *bHLH3* were determined in the F2 population by qRT-PCR (Fig. 6e, f). The transcript level of *bHLH3* was highest in F2-R, followed by F2-P, and lowest in F2-W, identical to the transcription pattern of *ANS*. The expression levels of *MYBA* did not differ significantly among the three F2 populations. To further investigate the reason for the low expression of *bHLH3* in pale-colored fruits, we analyzed the promoter sequence of *bHLH3* in CS, BYW, and HG2 because *cis*-acting elements are the main factors determining gene expression. Unexpectedly, except for a CT-repeat element (a 5′-UTR Py-rich stretch), no significant SNP loci correlated with fruit pigmentation were found (Fig. S9a). The 5′-UTR Py-rich stretch is usually involved in increasing gene expression^34^, and it was the longest in pigment-rich fruits and short in pale-colored fruits. The promoters of *bHLH3* from CS, HG2, or BYW were linked with *GUS* and transformed into tobacco leaves to test whether this CT-repeat element affects expression. The leaves transiently expressing the three *bHLH3p:GUS* constructs exhibited the same GUS activities (Fig. S9b–e).

### MYB4 is activated by bHLH3 and represses anthocyanin and proanthocyanidin biosynthesis

An R2R3-MYB repressor with C1 and C2 repression motifs at its C-terminus, designated MYB4, caught our attention, as it was present in the connection network and was positively associated with *bHLH3* expression (Figs. S5a, 6b). The transcript level of *MYB4* was found to be highly correlated with those of *ANS* and *bHLH3* at different stages of fruit ripening and in white and pigment-rich fruits (Figs. 3b, 6f). Consistent with its role as a transcriptional repressor, MYB4 was localized in the nucleus of onion cells (Fig. S6a). In Y2H, BiFC, and split luciferase complementation assays, MYB4 interacted with bHLH3, GL3, and TTG1. In Y3H assays, MYB4 was able to form ternary complexes with bHLH and TTG1 (Fig. 4a–d). These results suggested that *MYB4* functions as a member of a negative feedback regulation mechanism to balance flavonoid accumulation in mulberry fruits.

**Fig. 7:**
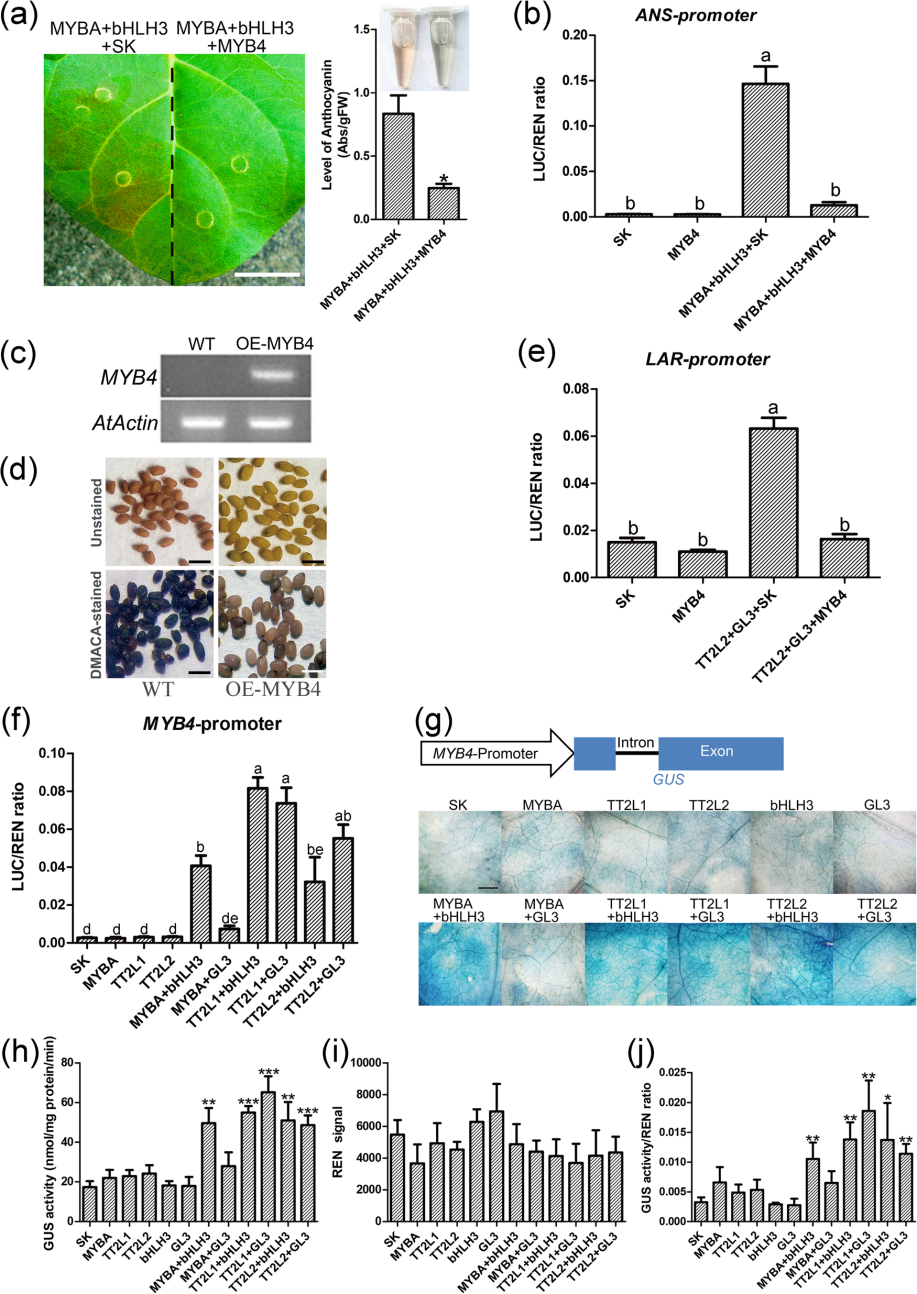
MYB4 participates in a negative feedback mechanism to balance the accumulation of anthocyanins and PAs. **a** Transient expression of *MYBA*, *bHLH3*, and *MYB4* in tobacco leaves. SK, empty vector. **b** Dual-luciferase reporter assays demonstrated that MYB4 can inhibit the activity of the *ANS* promoter. The data represent averages of four experiments. **c** The positive transgenic lines were determined by semiquantitative RT-PCR. *AtActin* served as an internal standard. **d** DMACA-stained (bottom row) and unstained (top row) seeds of non-transgenic wild-type *Arabidopsis* and transgenic *Arabidopsis* plants overexpressing *MYB4*. WT: wild-type, OE-MYB4: T2 seed of *MYB4*-transgenic plants. Scale bars are 1 mm. **e** Dual-luciferase reporter assays demonstrated that MYB4 can inhibit the activity of the *LAR* promoter. The data represent averages of four experiments. **f** Effect of MYB activators together with different bHLH cofactors on the activation of the *MYB4* promoter. SK, empty vector. The data represent averages of four experiments. **g** Validation of the activation effect of MYB/bHLH binary complexes on the *MYB4* promoter using a transgenic tobacco transient expression system. Transgenic tobacco plants expressing *GUS* driven by the *MYB4* promoter (transgenic line 1). GUS was expressed in transgenic tobacco leaves transiently transformed with candidate transcription factors and Renilla luciferase at a ratio of 10:1. The bar is 1 mm. **h** Measurement of GUS activity in transgenic tobacco leaves transiently transformed with candidate TFs and Renilla luciferase. **i** Measurement of Renilla luciferase activity in transgenic tobacco leaves transiently transformed with candidate TFs and Renilla luciferase. **j** Activation of the *MYB4* promoter by anthocyanin and PA activation complexes. A signal from Renilla luciferase was used as an internal control to normalize GUS activity. The data represent averages of three experiments. Asterisks (*) indicate the statistical significance of the difference between the experimental and the control groups. (Student’s *t* test, *P < 0.05, **P < 0.01, ***P < 0.001). Significant differences between treatments were determined using one-way ANOVA (*P* < 0.05)

To determine whether MYB4 inhibits flavonoid synthesis, transient color assays in tobacco leaves were conducted. Transformation of tobacco leaves with *MYBA*, *bHLH3*, and the empty vector resulted in red pigmentation after a week, whereas no pigmentation occurred when *MYBA* and *bHLH3* were coexpressed with *MYB4* (Fig. 7a). Dual-luciferase reporter assays supported this result; infiltration of *MYBA*, *bHLH3*, and the empty vector activated the *ANS* promoter, while replacing the empty vector with *MYB4* led to significantly decreased *ANS* promoter activity (Fig. 7b). To test whether MYB4 also negatively affects PA accumulation, we heterologously expressed *MYB4* driven by the CaMV 35S promoter in *Arabidopsis*. As expected, wild-type *Arabidopsis* accumulated abundant PAs in the seed coat, conferring a brown color. In contrast, transgenic plants overexpressing *MYB4* formed seeds with a pale-yellow seed coat that could not be stained with *p*-dimethylamino-cinnamaldehyde (DMACA), suggesting a very low PA content (Fig. 7c, d). In the dual-luciferase assay, addition of *MYB4* to the *TT2L2*/*GL3* complex reduced *LAR* promoter activity (Fig. 7e).

We further investigated whether bHLH3 participates in the activation of *MYB4* expression. In in vivo dual-luciferase reporter assays, the promoter of *MYB4* was activated by the combination of MYBA and bHLH3. In addition, both TT2L1 and TT2L2 activated the promoter of *MYB4* when interacting with bHLH3 or GL3 (Fig. 7f). To verify this result, we generated transgenic tobacco plants expressing *GUS* driven by the *MYB4* promoter. Then, each gene was transiently expressed in this background to determine whether it was able to activate the *MYB4* promoter, as detected by monitoring GUS activity. Compared with tobacco leaves expressing the empty vector or a single component, those transiently expressing a binary complex, such as *MYBA/bHLH3*, *TT2L1/bHLH3*, *TT2L1/GL3*, *TT2L2/bHLH3*, or *TT2L2/GL3*, had high GUS activity (Fig. 7g–j).

## Discussion

### bHLH3 is indispensable for regulation of the flavonoid pathway in mulberry fruits

The MYB–bHLH–WD40 transcriptional complex is a highly conserved model for the regulation of the flavonoid pathway, and activator-type MYBs primarily determine the target genes^35^. In this study, we identified five R2R3-MYBs, two bHLHs from subgroup IIIf, and one TTG1 protein involved in the regulation of flavonoid synthesis in mulberry fruits. Our results show that, except for MYBF and TT2L3, the other MYB factors, namely, MYBA, TT2L1, and TT2L2, interact with bHLH3 or GL3 and form an MBW complex with TTG1. Despite equal interaction strength within the different complexes, specific target genes are activated only by the complex formed by specific MYB and bHLH proteins. For example, although MYBA interacts with bHLH3 or GL3 to form an MBW complex, only the combination of MYBA and bHLH3 activates the expression of anthocyanin biosynthetic genes (*CYP75B1*, *ANS*, and *UFGT*) to control anthocyanin biosynthesis. While MYBA works only with bHLH3, the TT2L1 and TT2L2 proteins interact with bHLH3 or GL3 to activate the expression of structural genes. For example, the TT2L1/GL3 and TT2L2/GL3 complexes activate the expression of *LAR*, while TT2L1/bHLH3 activates the promoters of *CHS2*, *CYP75B1*, and *F3H*. Similarly, TT2L2/bHLH3 activates the promoters of *DFR* and *CHI*. We found that overexpression of *TT2L1* or *TT2L2* in tobacco led to increased PA content, which is indicative of the regulatory role of these genes in PA biosynthesis. In contrast, MYBF activates *FLS1*, *FLS3*, and *UFGT* and regulates flavonol biosynthesis without any partner.

Several studies have shown that the differences in anthocyanin levels in the same tissue among certain plant cultivars are mainly attributed to the MYB component of the MBW complex^36–38^. For example, *PavMYB10.1* determines the fruit skin color in sweet cherry, while its truncated allele, *PavMYB10.1b*, reduces the biosynthesis of anthocyanins in the cultivar ‘Rainier’^37^. In potato, *Stan2* is indispensable for the regulation of the biosynthesis of purple and red anthocyanins in tuber skin. This gene is expressed in the tuber skin of purple-colored and red-colored progeny but not in white tubers, implying that its expression determines the color of tuber skin^38^. In this study, the gene products of *MYBA* and *bHLH3* did not differ between purple fruits and pale-colored fruits, and we did not detect any splice site alterations or premature stop codons in the alleles of pale-colored fruits. Although *MYBA* and *bHLH3* participate in the regulation of anthocyanin biosynthesis, only *bHLH3* transcript levels were correlated with those of early (EBG) and late (LBG) anthocyanin biosynthesis genes between purple and pale-colored fruits. Additionally, in the F2 population derived from LJ109 and ZZB, the expression of *bHLH3* was highly correlated with that of *ANS* and with the degree of fruit coloration, but the transcript levels of *MYBA* did not differ among the F2 population. Because bHLH3 is a shared partner of MYBA, TT2L1, and TT2L2, it can form three MBW complexes to control the biosynthesis of flavonoids in mulberry fruits. This finding suggests that bHLH3 is the backbone of the flavonoid biosynthetic regulation network. However, the reason for the differences in *bHLH3* transcript levels between pigment-rich and pale-colored fruits remains unknown.

*Cis* and *trans*-acting elements are considered to be the main factors determining gene expression^39^. Except for the 5′-UTR Py-rich stretch element, no significant SNP loci correlated with fruit pigmentation were found in the promoters of *bHLH3* in CS, BYW, and HG2. The 5′-UTR Py-rich stretch is associated with high transcription^34^. This stretch was longest in the pigment-rich mulberry fruits and short in the pale-colored fruits, but the promoters of *bHLH3* from CS, HG2, and BYW exhibited the same transcriptional activities. Instead, the difference in *bHLH3* expression between pigment-rich and pale-colored fruits may be caused by *trans*-acting elements. In the connection network constructed based on anthocyanin compounds and transcripts, 36 transcription factors were strongly correlated with the expression of *bHLH3* (Fig. 6b, Table S10). These candidate transcription factors possibly have upstream and downstream regulatory relationships with bHLH3. For example, *L484_026215*, *L484_003860*, and *L484_017595* are homologs of *Arabidopsis GL2*, *TTG2*, and *TRY*, respectively, and their transcript levels were positively correlated with the expression of *bHLH3* in the connection network. In *Arabidopsis*, *AtGL2* expression is activated by MBW activator complexes, and then, AtGL2 regulates mucilage synthesis and cell fate determination^40^. Additionally, *AtGL2* has a negative regulatory effect on anthocyanin synthesis by directly inhibiting the transcription of some MBW component regulators (e.g., *AtTT8*, *AtPAP1*, *AtPAP2*, *AtMYB113*, and *AtMYB114*)^41^. We propose that MBW complexes containing bHLH3 also regulate *L484_026215* in mulberry, and then, in turn, the product of *L484_026215* represses the expression of *MYB* and *bHLH*, which are involved in MBW complexes, to limit anthocyanin biosynthesis. Similarly, the MBW complex (AtTT2–AtTT8–AtTTG1) can directly activate *AtTTG2*^42^. In the regulation of trichome development, AtTTG2 regulates the trichome patterning R3 MYB gene *AtTRY* dependent on the MBW complex^42^. In petunia, *PH3*, the homolog of *Arabidopsis TTG2*, is activated by an MBW complex (PH4–AN1–AN11). Then, PH3 joins the MBW complex in regulating the transcription of *PH5* and *PH1* (encoding P-ATPase transmembrane transporters), which finally modify flower color by vacuole acidification^43^. Taken together, our results suggest that *L484_026215, L484_003860*, and *L484_017595* are downstream regulatory genes of *bHLH3*: the product of *L484_026215* acts as a negative feedback modulator to balance anthocyanin accumulation similarly to MYB4; *L484_017595* is regulated by *L484_003860*; and *L484_003860* is activated by the MBW complex containing bHLH3 and is involved in fruit color modification through vacuole acidification. Some of the transcription factors in the connection network may be involved in the abnormal expression of *bHLH3*, and these will be the focus of future research. Taken together, these findings imply that the active function of MYBA is dependent on bHLH3 and that bHLH3 plays a vital role in regulating the biosynthesis of flavonoids in mulberry fruits.

### MYB4 acts as a negative feedback modulator to balance anthocyanin and PA accumulation in mulberry

In this study, *bHLH3* was positively correlated with the expression of not only some structural genes but also some transcription factors. The transcription of *MYB4* was highly correlated with that of *ANS* and *bHLH3* and with the degree of fruit coloration (i.e., high transcript levels in purple fruits but low levels in pale fruits). Similar to previously reported MYB repressors such as poplar PtMYB182^44^, PtMYB165^45^, strawberry FaMYB1^46^, peach PpMYB18^47^, and grapevine VvMYBC2-L1^48^, MYB4 is a typical R2R3-MYB repressor with C1 and C2 repression motifs at its C-terminus and is clustered in the same anthocyanin/PA clade within subgroup 4 R2R3-MYBs. Our results show that MYB4 interacts with bHLH3 and GL3 and then forms a ternary complex with TTG1. In dual-luciferase reporter assays, the addition of MYB4 to MYBA/bHLH3 and TT2L2/GL3 complexes reduced the activities of the *ANS* and *LAR* promoters, respectively. Furthermore, heterologous overexpression of *MYB4* in *Arabidopsis thaliana* and tobacco demonstrated that it is a negative regulator of both PA and anthocyanin pathways. These findings suggest that MYB4 acts as a repressor of anthocyanin and PA biosynthesis by disrupting the interaction between the MYB activator and bHLH, similar to petunia PhMYB27 and peach PpMYB18^47,49^.

The MYB repressor CmMYB#7 functions as a negative regulator of anthocyanin biosynthesis in chrysanthemum, where it is highly expressed in white flowers but weakly expressed in red flowers^50^. In contrast, although MYB4 also functions as a repressor, its expression was positively correlated with the degree of mulberry fruit coloration, indicating that MYB4 cannot completely inhibit anthocyanin accumulation. Based on the strong positive correlation between *MYB4* and *bHLH3* at the transcriptional level, it is likely that *MYB4* is induced by anthocyanin activators, thus preventing the overaccumulation of anthocyanins. Here, in addition to being activated by MYBA/bHLH3, *MYB4* could also be activated by PA activation complexes, including TT2L1/bHLH3, TT2L1/GL3, TT2L2/bHLH3, and TT2L2/GL3. These results suggest that MYB4 participates in a negative feedback mechanism to balance anthocyanin and PA accumulation. First, MYBA/bHLH3 activates the biosynthesis of anthocyanins, and TT2L1 and TT2L2 positively regulate the synthesis of PAs with GL3. At the same time, these complexes as well as TT2L1/bHLH3 and TT2L2/bHLH3 activate the expression of *MYB4*. Finally, MYB4 competes with MYB to interact with bHLH and disrupts the formation of MBW complexes, thereby inhibiting the synthesis of anthocyanins and PAs. Anthocyanins can protect plants against a variety of biotic and abiotic stresses because of their powerful antioxidant properties^51^. However, excessive accumulation of anthocyanins is toxic to plant cells^52^. Therefore, plants have evolved detoxification mechanisms to avoid self-toxicity, such as vesicle transport, vacuolar sequestration, extracellular biosynthesis, extracellular excretion, and accumulation of these metabolites in nontoxic form^52,53^. The anthocyanin content is very high in mulberry fruits, higher than that in black bilberry and blackberry fruits^54^. Therefore, to avoid excessive accumulation of toxic flavonoids, mulberry may have developed a hierarchical and negative feedback mechanism using MYB4 as a negative feedback modulator to balance the synthesis of anthocyanins and PAs and maintain these compounds at appropriate concentrations.

### Abnormal *bHLH3* expression disrupts the homeostatic regulatory network, causing differences in pigment composition among mulberry fruits

**Fig. 8:**
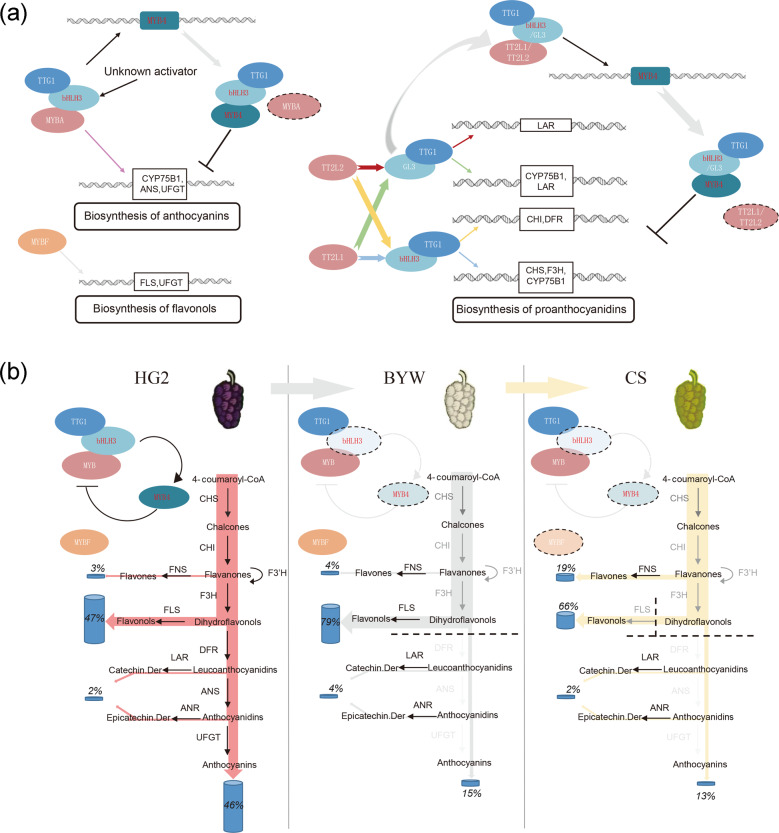
Proposed model for differences in fruit color among mulberry cultivars. **a** Constructed flavonoid homeostasis network of activation and feedback regulation mechanisms in mulberry fruits. MYBA-bHLH3-TTG1 regulates anthocyanin biosynthesis, and TT2L1/TT2L2-bHLH3/GL3-TTG1 regulate the biosynthesis of PAs. bHLH3 is the key component of the regulatory network and participates in the regulation of most structural genes. All these MBW complexes activate the expression of *MYB4*, and then, MYB4 inhibits the biosynthesis of anthocyanins and PAs in a competitive manner to prevent plants from producing excessive anthocyanins and PAs. MYBF regulates flavonol biosynthesis without any partner. An unknown activator may determine *bHLH3* expression. **b** Altered pigmentation in mulberry fruits caused by differences in *bHLH3* expression. In HG2 (purple fruits), flavonoid biosynthesis is controlled in an orderly manner by regulators in this regulatory network. In BYW (white fruits) and CS (yellow fruits), abnormal expression of *bHLH3* disrupts this dynamic equilibrium mechanism: *MYB4* cannot be effectively activated by bHLH3, causing the feedback regulation mechanism to fail during fruit pigmentation, and the MBW complexes dependent on bHLH3 cannot effectively activate their target genes, thereby reducing flavonoid accumulation. A decrease in *DFR* expression causes carbon flux to the upstream branch, resulting in higher proportions of flavonols in CS and BYW than in HG2. Because of the low expression of *MYBF* and the high expression of *FNS* in CS, blockage of the anthocyanin and flavonol pathways diverts carbon flow to the upstream flavone pathway, resulting in the highest flavone content in CS

Generally, the interaction between activators and MYB4 constitutes a homeostasis regulatory network that controls the pigmentation of mulberry fruits (Fig. 8). This network involves the MYBA, TT2L1, TT2L2, bHLH3, GL3, TTG1, MYBF, and MYB4 proteins. MYBA/bHLH3, TT2L1/bHLH3, TT2L1/GL3, TT2L2/bHLH3, and TT2L2/GL3 activate flavonoid structural genes. The regulation of most genes, such as *CHS2*, *CHI*, *F3H*, *CYP75B1*, *DFR*, *ANS*, and *UFGT*, requires MBW complexes containing bHLH3, suggesting that bHLH3 occupies a key position in the flavonoid biosynthesis pathway. However, MYB4 functions as an important modulator to balance the biosynthesis of anthocyanins and PAs in mulberry fruits. MYB4 is activated by the MBW complexes that regulate PA and anthocyanin pathways, and it inhibits the excessive biosynthesis of anthocyanins and PAs in a competitive manner. The results of our study reveal that flavonoid biosynthesis in mulberry is controlled in an orderly manner by regulators in the homeostasis regulatory network. This complex network is stable in pigment-rich fruits and may provide the means to match the activation response to stress or developmental cues via precise gene regulation. However, in pale fruits, there is abnormal expression of *bHLH3*, so this dynamic equilibrium mechanism is broken. That is, *MYB4* cannot be effectively activated by bHLH3, causing the feedback regulation mechanism to fail during fruit pigmentation. At the same time, the MBW complexes dependent on bHLH3 cannot effectively activate their target genes, thereby reducing flavonoid accumulation. DFR represents the entry point into the anthocyanin biosynthesis pathway, and its transcription requires the involvement of bHLH3. Therefore, a decrease in *DFR* expression causes carbon flux to the upstream branch, resulting in a higher proportion of flavonols in CS and BYW than in HG2. The expression level of *MYBF* is much lower in CS than in BYW, and accordingly, the carbon flux to the flavonol pathway is more limited in CS than in BYW. The high expression of *FNS* in CS promotes the flavone biosynthesis pathway to capture the carbon flux that is blocked downstream, resulting in the highest content of flavones in CS. Therefore, abnormal *bHLH3* expression disrupts the flavonoid homeostasis network, leading to differences in the levels and proportions of flavones, flavonols, and anthocyanins among the differently colored fruits of CS, HG2, and BYW.

Raspberry and blackberry, two berries with fruit morphology similar to that of mulberry, also have vibrant and diverse colors. The fruits of red and yellow raspberry cultivars can be clearly separated on a PCA plot based on their distinct metabolite profiles^55^. Compared with red raspberry fruits, yellow raspberry fruits lack anthocyanins, but they are rich in taxifolin and have similar compositions to red cultivars in terms of epicatechin and flavan-3-ols (except for procyanidin B1). The difference in raspberry fruit color is caused by mutation of *ANS*, which blocks the accumulation of anthocyanins and leads to the reconstruction of the flavonoid pathway in yellow raspberry cultivars^55,56^. The flavonoid components in blackberry fruits differ markedly among the three stages of fruit ripening^57^. The green fruits contain abundant flavonols and catechins but no anthocyanins. Red fruits have low levels of flavonols and catechins but a high proportion of anthocyanins in the total flavonoids. Black fruits contain higher levels of flavonols than red fruits, and more than 90% of the flavonoids are anthocyanins. All of these changes are attributed to the redirection of the flavonoid pathway caused by spatiotemporal differences in the expression of regulatory factors and structural genes^57,58^. Despite the high expression levels of genes involved in flavonol synthesis, the low transcription level of the gate gene (*RuFLS*) in the flavonol pathway and overexpression of *ANS* regulated by RuMYB10 lead to increased capture of carbon flux by the anthocyanin pathway^57,58^. Changes in the coloration of plant tissues attributed to the redirection of flavonoid biosynthesis have also been described for other horticultural crops. Mutations in *ScbHLH17* and *ScCHI1/2* block the biosynthesis of anthocyanins in white and yellow *Senecio cruentus* cultivars, respectively^59^. The large amount of chalcones accumulated as a result of the *ScCHI1/2* mutation cannot move downstream, so the flowers are yellow. The carmine, pink, and blue cultivars mainly accumulate cyanidin, pelargonidin, and delphinidin in different amounts^59^. The anthocyanins in pink flowers are 99% pelargonidin and 1% cyanidin, those in blue flowers are 83% delphinidin and 17% cyanidin, and those in carmine flowers are 8% delphinidin and 92% cyanidin. This is essentially because the competition among the gene products of *ScDFR1/2*, *ScF3*′*5*′*H*, and *ScF3*′*H1* for dihydrokaempferol results in differences in the metabolic flux into the pelargonidin, delphinidin, and cyanidin pathways^59^. Generally, anthocyanins are the main pigments in plant reproductive organs, but flavones and flavonols with pale-yellow colors are also copigments in plant tissues^2^. When copigmentation occurs, anthocyanins chemically interact with other flavonoids^60^, and then, their colors mix or blend, conferring different colors to mulberry fruits. Therefore, we suggest that the sophisticated regulation network in mulberry is similar to a spider web: pulling on one strand causes all the strands to move. The results of our research systematically reveal the reason for the different colors of mulberry fruits and provide a unique perspective for explaining the polymorphism of plant tissue color.

## Materials and methods

### Plant materials

All the plant materials used in this work are shown in Table S7. LJ109 (*Morus alba* L. cv. Lunjiao109, male parent) was crossed with ZZB (*Morus alba* L. cv. Zhenzhubai, female parent). The F1 population was segregated into four phenotypes: white fruits, alternating red and white fruits, red fruits, and male mulberry. The F2 population was derived from a cross between a male mulberry and a female parent with white fruits from the F1 population. The F2 population was divided into four phenotypes: white fruits, alternating red and white fruits, red fruits, and male mulberry.

### In vivo characterization of CYP75B1 and CY75B2 in yeast

*CYP75B1* and *CY75B2* cloned from *M. notabilis* C. K. Schneid (CS) were each recombined into the pYES2 vector with a Ura selection marker. The gene encoding mulberry P450 reductase (L484_012842) was recombined into the pYES2 vector in which the Ura selection marker was replaced with a Leu marker (Clontech, Palo Alto, CA, USA) as described by Shang et al.^61^. The combination of *CYP75B1*-pYES2 (Ura) and *L484_012842*-pYES2 (Leu) or the combination of *CYP75B2*-pYES2 (Ura) and *L484_012842*-pYES2 (Leu) was then transferred to the INVSc1 yeast strain. Functional assays of candidate genes were performed as described by Liu et al.^62^.

### Quantification of flavonols, PAs, and anthocyanins

The anthocyanin content (*Q*) was determined as *Q* = (*A*_530_ − 0.25 × *A*_657_)/*M*^63^. The concentration of PAs in *Arabidopsis* was determined using the DMACA assay as described by Abrahams et al.^64^. The flavonols and PAs in transgenic tobacco and the products of the cell feeding assay in yeast were identified and quantified by ultraperformance liquid chromatography (UPLC) using the protocols described in Methods S1.

### Metabolomics analysis and transcriptome sequencing

Metabolite profiling was performed using an LC–ESI–MS/MS system (UPLC, Shim-pack UFLC CBM20A, Shimadzu, Kyoto, Japan; MS/MS, AB Sciex QTRAP4500 System, Applied Biosystems, Foster City, CA, USA). The extraction, identification, and annotation of the metabolites in the sample fruits are described in detail in Methods S2. The data from nine samples (three cultivars × three biological replicates) were processed by OPLS-DA and PCA to detect differences in metabolic composition between the different colored fruits.

RNA-seq and *de novo* transcriptome assembly in mulberry were carried out as described by Shang et al.^65^. The DEGs between the samples were determined according to the following criteria: false discovery rate (FDR) threshold <0.05 and fold change ≥2. Transcripts were annotated by searches against the NCBI nonredundant (nr) database, the KEGG database^66^, and the UniProtKB/Swiss-Prot database using the BLASTx program (*E*-value ≤ 10^−5^). Cytoscape v3.4.0 was used to visualize the relationship between the transcriptome and metabolome. Pearson’s correlation coefficients and *P*-values for transcriptome and metabolome data were calculated as described by Cho et al.^67^.

### Isolation and phylogenetic analysis of MYB, bHLH, and TTG1 regulators

*Arabidopsis* MYB, bHLH, and TTG1 regulators participating in the regulation of flavonoid biosynthesis were used as queries in TBLASTN searches against our transcriptome database and the *M. notabilis* genome^68^. The identified genes were then cloned from *M. alba* L. cv. Hongguo2. Multiple protein sequence alignment and phylogenetic analyses were carried out as previously described^69^.

### Subcellular localization of MYB, bHLH, and TTG1 regulators

The ORFs of all candidate genes (without stop codons) were individually cloned into the pZYGC vector (GFP at the C-terminus) to generate a GFP-fused protein as described by Lv et al.^70^. The recombinant plasmids were transiently transformed into onion epidermal cells by *Agrobacterium*-mediated transfection. After 2 days of culture, the samples were scanned under an FV1200 laser scanning confocal microscope (Olympus, Tokyo, Japan).

### Real-time PCR analysis

Quantitative real-time PCR was conducted using an Applied Biosystems StepOnePlus™ real-time PCR machine with the method described previously^69^. A housekeeping gene (*MaACTIN*, *NtACTIN*, or *AtACTIN*) served as the internal standard. The primers for qRT-PCR and RT-PCR are shown in Supplementary Table S8.

### Y2H, BiFC, Y3H, and split luciferase complementation assays

For Y2H assays, *MYBA*, *TT2L1*, *TT2L2*, *TT2L3*, *MYBF*, *MYB4*, and *TTG1* were each recombined into pGADT7 and pGBKT7. For BiFC assays, the ORFs of *MYBA*, *TT2L1*, *TT2L2*, *TT2L3*, *MYB4*, *bHLH3*, *GL3*, and *TTG1* were each recombined into the vector pSPYNE. The CDS of *bHLH3*, *GL3*, or *TTG1* was recombined into the vector pSPYCE. Both Y2H and BiFC assays were carried out as described by Lv et al.^70^.

The Y3H assays were carried out in the Y2HGold yeast strain background. The ORF of *TTG1* was recombined into pBridge at MCS I and that of *bHLH3* or *GL3* was recombined into pBridge at MCS II. The interactions among fused proteins expressed from the recombinant pBridge and pGADT7 vectors were analyzed as described by Chakravorty et al.^71^.

Split luciferase complementation assays were conducted as described by Chen et al.^72^. The CDSs of *MYBA*, *TT2L1*, *TT2L2*, *TT2L3*, *MYB4*, *bHLH3*, *GL3*, and *TTG1* (without stop codons) were each recombined into the pCambia1300nLUC vector, and *bHLH3*, *GL3*, and *TTG1* were each recombined into the pCambia1300cLUC vector. The protocol of *Agrobacterium* cultivation and infiltration was described by Zhou et al.^73^. Three days after infiltration, the leaf disks adjacent to the infiltration site were collected by punching, and the firefly luciferase activity in the samples was detected using the Steady-Glo Luciferase Assay Kit (Promega, Madison, WI, USA).

### Y1H assays

The promoter fragments of *ANS*, *LAR*, and *FLS1* cloned from *M. notabilis* C. K. Schneid were inserted into the pBait-AbAi vector to construct bait yeast strains. The fusion proteins with GAL4 AD as constructed for Y2H assays were then transferred into recombinant bait yeasts. According to the manufacturer’s protocol, the assays were conducted using the Matchmaker® Gold Y1H System (Clontech, Japan).

### Dual-luciferase reporter assay

*MYBA*, *TT2L1*, *TT2L2*, *MYBF*, *bHLH3*, *GL3*, *TTG1*, and *MYB4* were each recombined into the effector vector pGreenII 62-SK. The promoter fragments of *CHS2*, *CHI*, *F3H*, *FNS*, *CYP75B1*, *CY75B2*, *FLS3*, *DFR*, *UFGT*, *ANR*, and *MYB4* were each cloned from *M. notabilis* CS and then inserted into the pGreenII 0800-LUC reporter vector. *Agrobacterium*-mediated cotransformation of the reporter and effector constructs into tobacco leaves was carried out as previously described^73^. After 3 days of infiltration, the ratio of LUC activity to Ren activity was detected using the Dual-Glo® Luciferase Assay System (Promega Madison, WI, USA) on a multimode microplate reader (Synergy™ H1, BioTek, USA).

### Transformation of tobacco and *Arabidopsis*

*MYBA*, *TT2L1*, *TT2L2*, *MYBF*, *MYB4*, *bHLH3*, and *GL3* were each cloned into the pLGNL vector and then transformed into *Agrobacterium tumefaciens* GV3101. The *Arabidopsis* mutant *tt8-4* is in the Col-0 background. Transformation of tobacco (K326) and *Arabidopsis* (Col-0 type) was performed as described by Pattanaik et al.^74^ and Clough and Bent^75^, respectively.

### Transient expression studies in tobacco

The promoter fragment of *MYB4* cloned from *M. notabilis* CS was used to replace the 35S promoter driving *GUS* in pCAMBIA1301 and then transformed into tobacco as described above. Transient GUS expression was induced in transgenic tobacco leaves after infiltration of the effectors (*A. tumefaciens* GV3101 containing recombinant pGreenII 62-SK plasmids for the dual-luciferase reporter assay) and Renilla luciferase (*A. tumefaciens* GV3101 containing the vector pGreenII 0800-LUC) at a ratio of 10:1, as described for the dual-luciferase reporter assay. Three days after infiltration, three quarters of the materials were used for GUS activity detection, and one quarter was used for Renilla luciferase activity detection using the Dual-Glo® Luciferase Assay System. The GUS activity analysis was carried out as described by Ma et al.^76^. The signal from Renilla luciferase was used as an internal control to normalize GUS activity.

The transient color assay was conducted in young tobacco plants (2-week-old seedlings). *A. tumefaciens* GV3101 transformed with pSoup-19 and recombinant pGreenII 62-SK plasmids as described above was used in the transient color assays. *Agrobacterium* cultivation and infiltration were carried out as described by Zhou et al.^73^. Photos were taken at 7 days after infiltration.

To investigate the activities of the *bHLH3* promoters from CS, BYW, and HG2, the promoter fragments of *bHLH3* were cloned from CS, BYW, and HG2, and then, each one was linked with *GUS* in the vector pCAMBIA1301. The recombinant plasmids (*A. tumefaciens* GV3101 containing recombinant pCAMBIA1301 vector) and Renilla luciferase (*A. tumefaciens* GV3101 containing vector pGreenII 0800-LUC) were transiently transformed into tobacco at a ratio of 10:1 as described for the dual-luciferase reporter assay. The GUS and Renilla luciferase activities were quantified as described above. The signal from Renilla luciferase was used as the internal control.

### Accession numbers

The RNA-Seq data in this work have been uploaded to NCBI under BioProject PRJNA561457. The accession numbers of all sequences used in this study are listed in Table S9.

## Supplementary information

Supplementary Figures

Supplementary Methods

Supplementary Tables
