# Supplementary material for: Abnormal expression of bHLH3 disrupts a flavonoid homeostasis network, causing differences in pigment composition among mulberry fruits
Source: Hortic Res. 2020 Jun 1;7:83. doi: 10.1038/s41438-020-0302-8 (PMC7261776; doi:10.1038/s41438-020-0302-8)
Supplement: Supplementary file 1 — Supplementary Figures [file 41438_2020_302_MOESM1_ESM.docx]

**
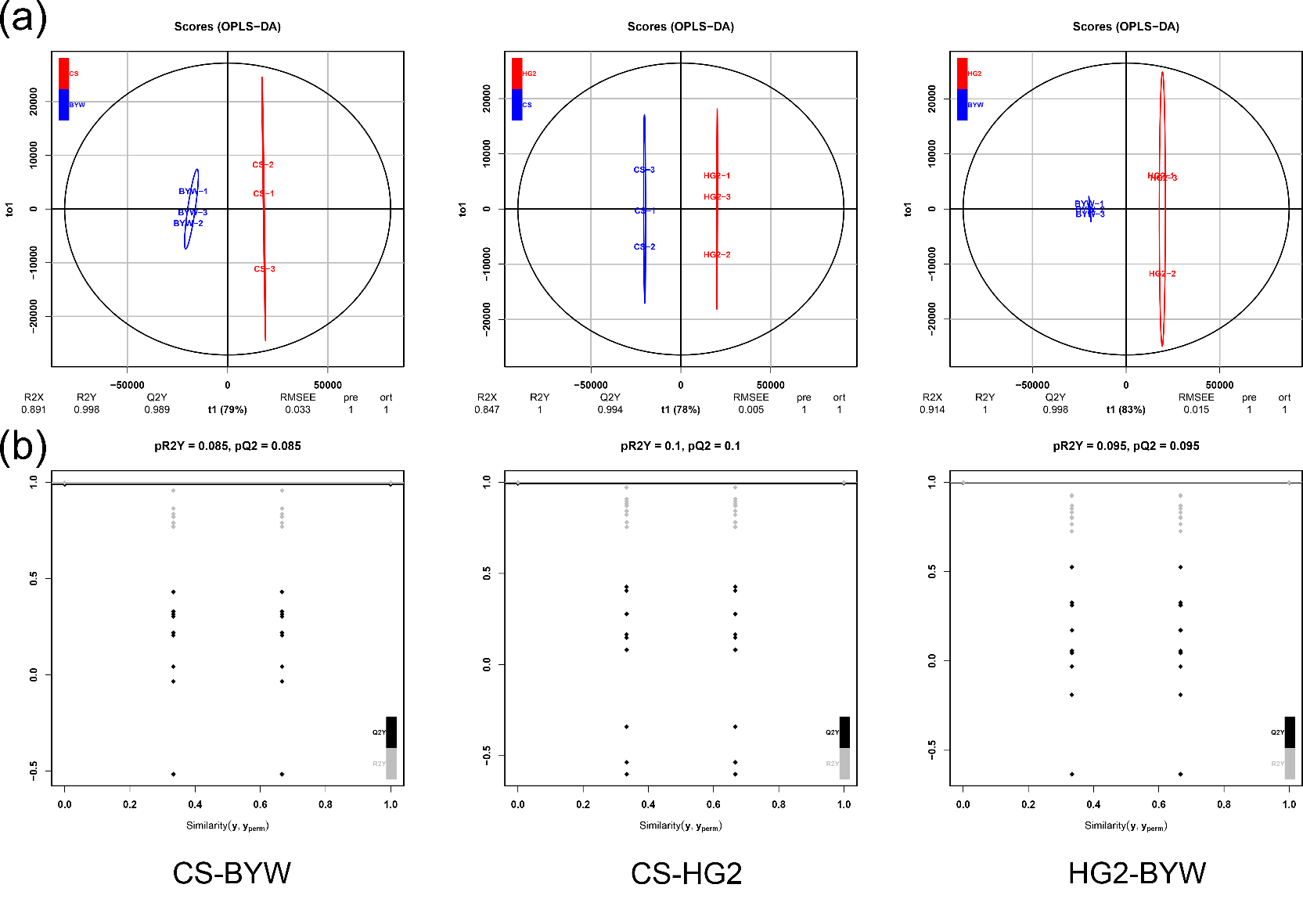
**

**Supplemental Fig. S1.** **OPLS-DA score plots for different mulberry cultivars.** **(a)** Model verification: cross-validation. R2X and R2Y respectively represent the interpretation rate of the model for the X and Y matrices, and Q2 represents the predictive ability of the model. The closer the three indicators are to 1, the more stable and reliable the model is. When Q2>0.5, the model prediction ability is good, and Q2>0.9 is an excellent model. **(b)** Model verification: Permutation test. The two rightmost points (x=1.0) are R2 and Q2 of the original model, and all the points on the left are R2' and Q2' of the model after Y replacement. If these R2' and Q2' are smaller than the original R2 and Q2, then the model makes sense.


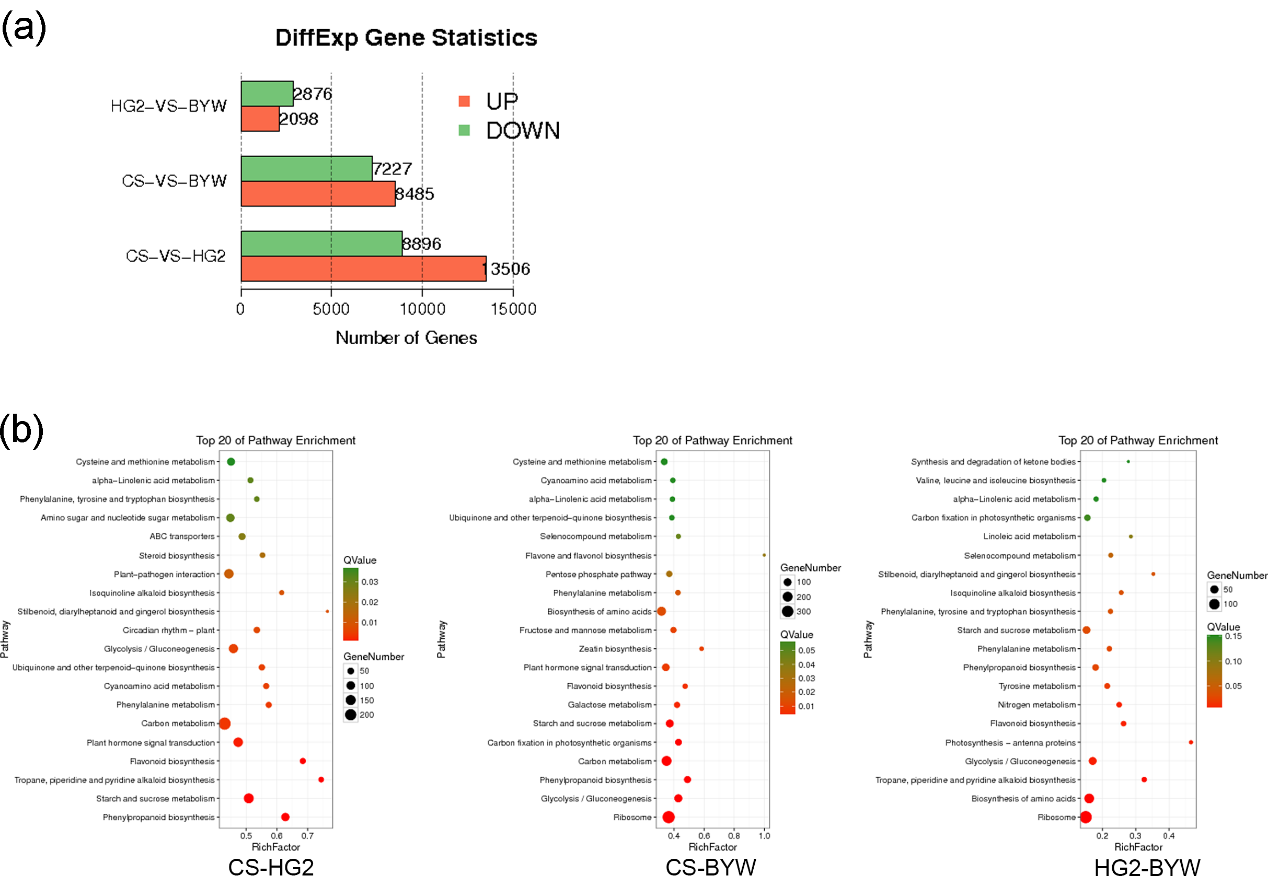


**Supplemental Fig. S2.** **KEGG pathway enrichment of DEGs between different mulberry cultivars.** **(a)** Quantitative statistics of the differentially expressed genes among CS, HG2 and BYW. **(b)** Top 20 KEGG pathway enrichment of DEGs between different mulberry cultivars. Number of DEGs is represented by the size of the circle.


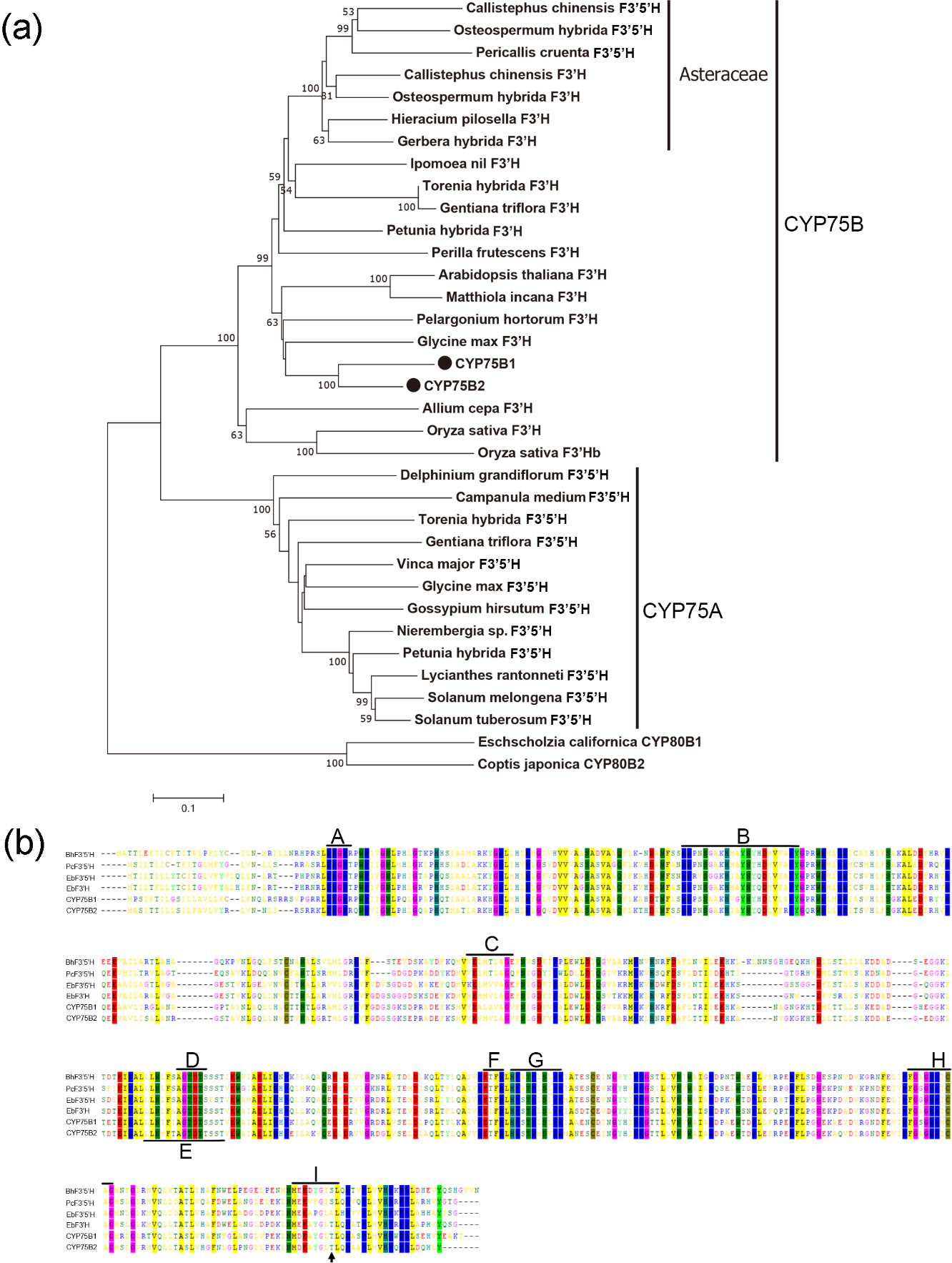


**Supplemental Fig. S3.** **CYP75B subfamily genes in mulberry.** **(a)** Phylogenetic tree derived from amino acid sequences of *CYP75A* and *CYP75B* genes in mulberry and other species. **(b)** Alignment of the amino acid sequences of mulberry CYP75Bs with other CYP75A and CYP75B proteins. A: hinge region, B: substrate recognition site 1 (SRS1), C: SRS2, D: oxygen binding pocket, E: SRS4, F: ExxR motif, G: SRS5, H: heme binding domain, and I: SRS6. The position corresponding to the previously described functional determinant for F3’H activity is indicated by an arrow **^1^**.


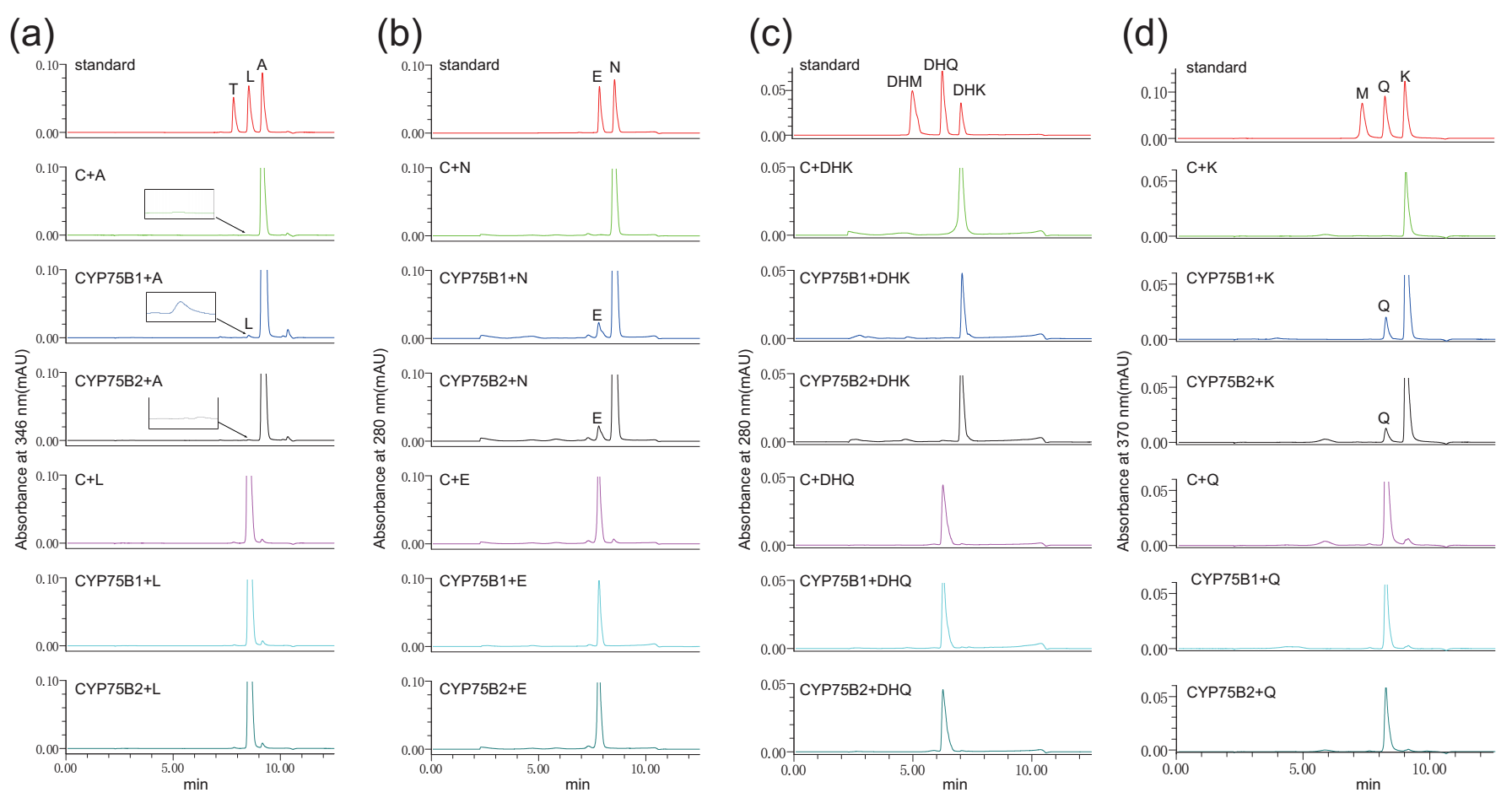


**Supplemental Fig. S4.** **Functional characterization of CYP75B1 and CYP75B2.** *In vivo* characterization of CYP75B1 and CYP75B2 by coexpression with mulberry P450 reductase (L484_012842) in INVSc1 yeast strain. C: yeast-expressed P450 reductase, CYP75B1: yeast-coexpressed CYP75B1 and P450 reductase, CYP75B2: yeast-coexpressed CYP75B2 and P450 reductase. **(a)** L: Luteolin, A: Apigenin, T: Tricetin. **(b)** N: Naringenin, E: Eriodictyol. **(c)** DHQ: Dihydroquercetin, DHK: Dihydrokaempferol, DHM: Dihydromyricetin. **(d)** Q: Quercetin, K: Kaempferol, M: Myricetin.


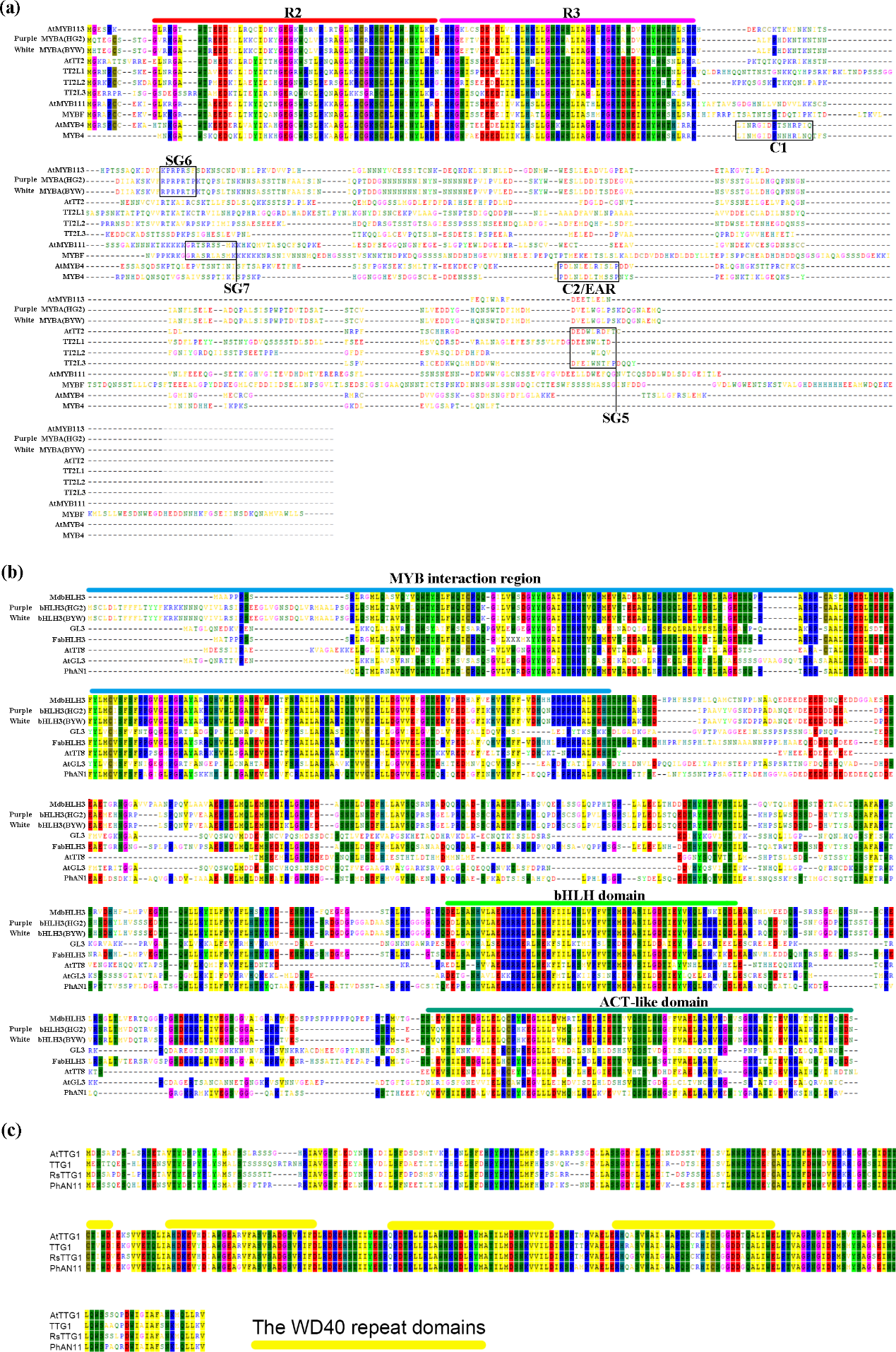


**Supplemental Fig. S5.** **Multiple sequence alignment of candidate transcription factors and other flavonoid-related regulatory factors from different plants.** **(a)** “Purple” represents the transcription factor in purple fruit (HG2). “White” represents the transcription factor in white fruit (BYW). The R2 and R3 domains are indicated with red and purple boxes, respectively, whereas conserved motifs in the C-terminus were boxed and numbered. **(b)** The MYB interaction region, bHLH and ACT-like domains are indicated with blue, cyan-green and green boxes, respectively. **(c)** The WD40 repeat domains are indicated with yellow boxes.


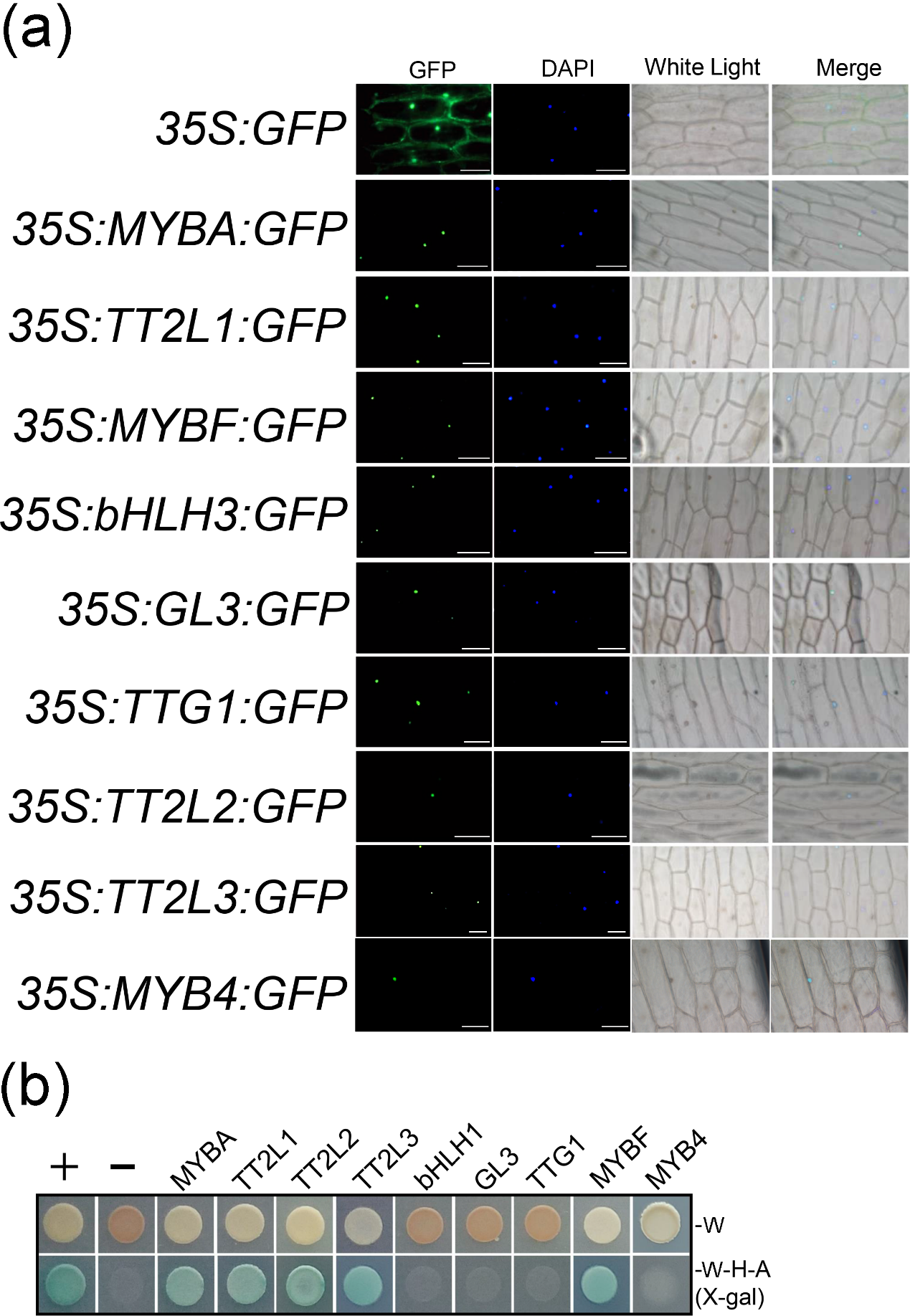


**Supplemental Fig. S6.** **Subcellular localization and transcriptional activity analysis of candidate genes. (a)** Subcellular localization of candidate transcription factors in onion epidermal cells. The position of nuclei was confirmed by DAPI staining and bright-filed images were compared. Scale bars are 100 μm. **(b)** Transactivation activity analysis of candidate transcription factors in yeast. -W, SD -Trp medium; -W-H-A(X-gal), SD -Trp-His-Ade+X-a-Gal medium.


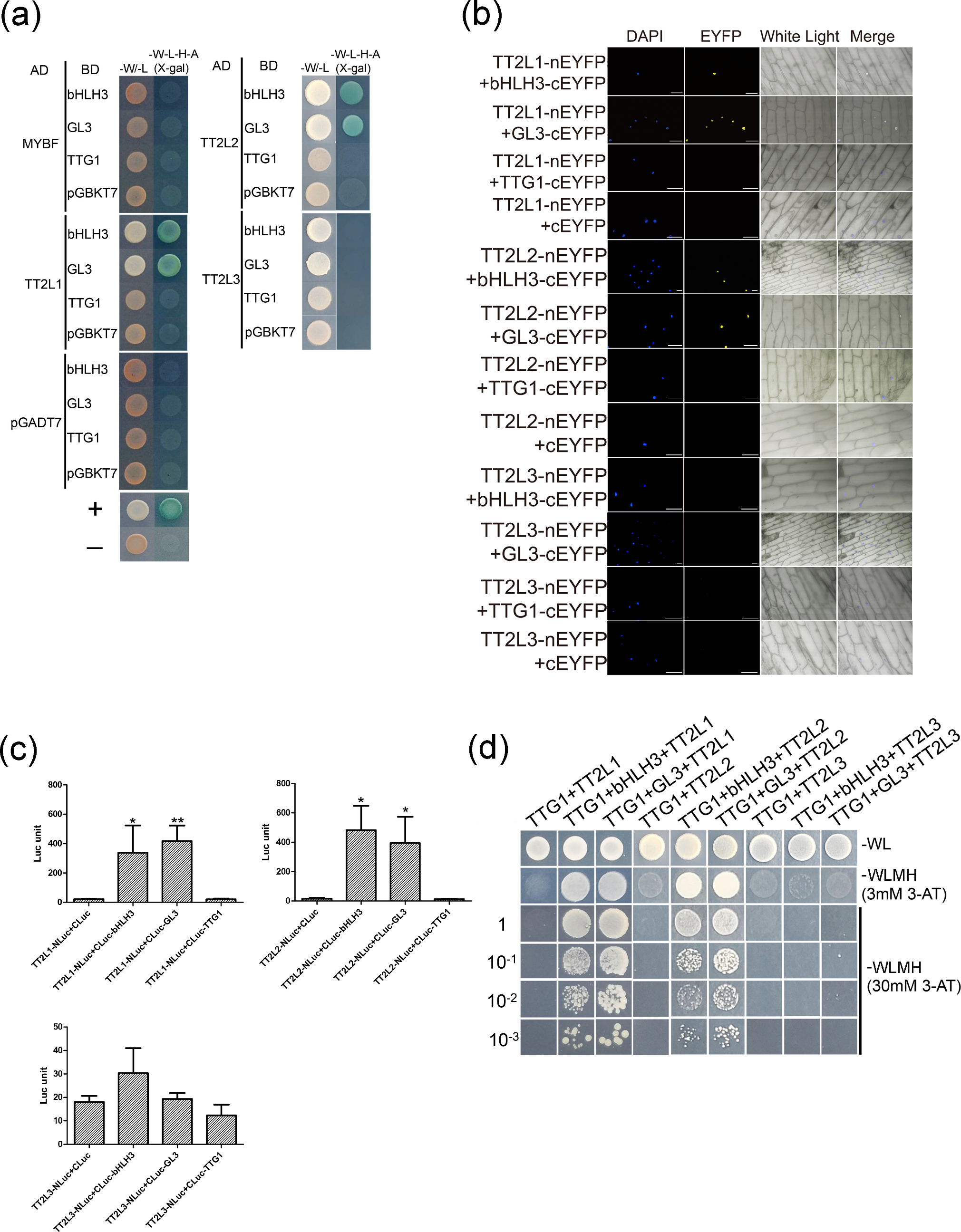


**Supplemental Fig. S7. Interaction between different regulators in MYB–bHLH–WD40 complexes. (a)** Yeast-two-hybrid assays. –W/-L, SD-Trp-Leu medium; -W-L-H-A(X-gal), SD-Trp-Leu-His-Ade+X-a-Gal medium. **(b)** Bimolecular fluorescence complementation assays. Scale bars are 100 μm. **(c)** Split luciferase complementation assays in *N. benthamiana* leaves. Data represent averages of 4 experiments. Asterisk (*) indicates statistical significance of difference between the experimental and the control groups. (Student’s *t* test, * *P* < 0.05; ** *P* < 0.01). **(d)** Yeast three-hybrid assays. The CDSs of TT2L1, TT2L2 and TT2L3 were recombined into pGADT7, respectively. The ORF of TTG1 was recombined into pBridge at MCS I, and that of bHLH3 or GL3 was recombined into pBridge at MCS II. Interactions were assayed on SC-Trp-Leu-Met-His supplemented with the indicated concentrations of 3-AT (0 and 30 mM). Transformed yeast cells were diluted 10-, 100-, and 1000- times on medium.


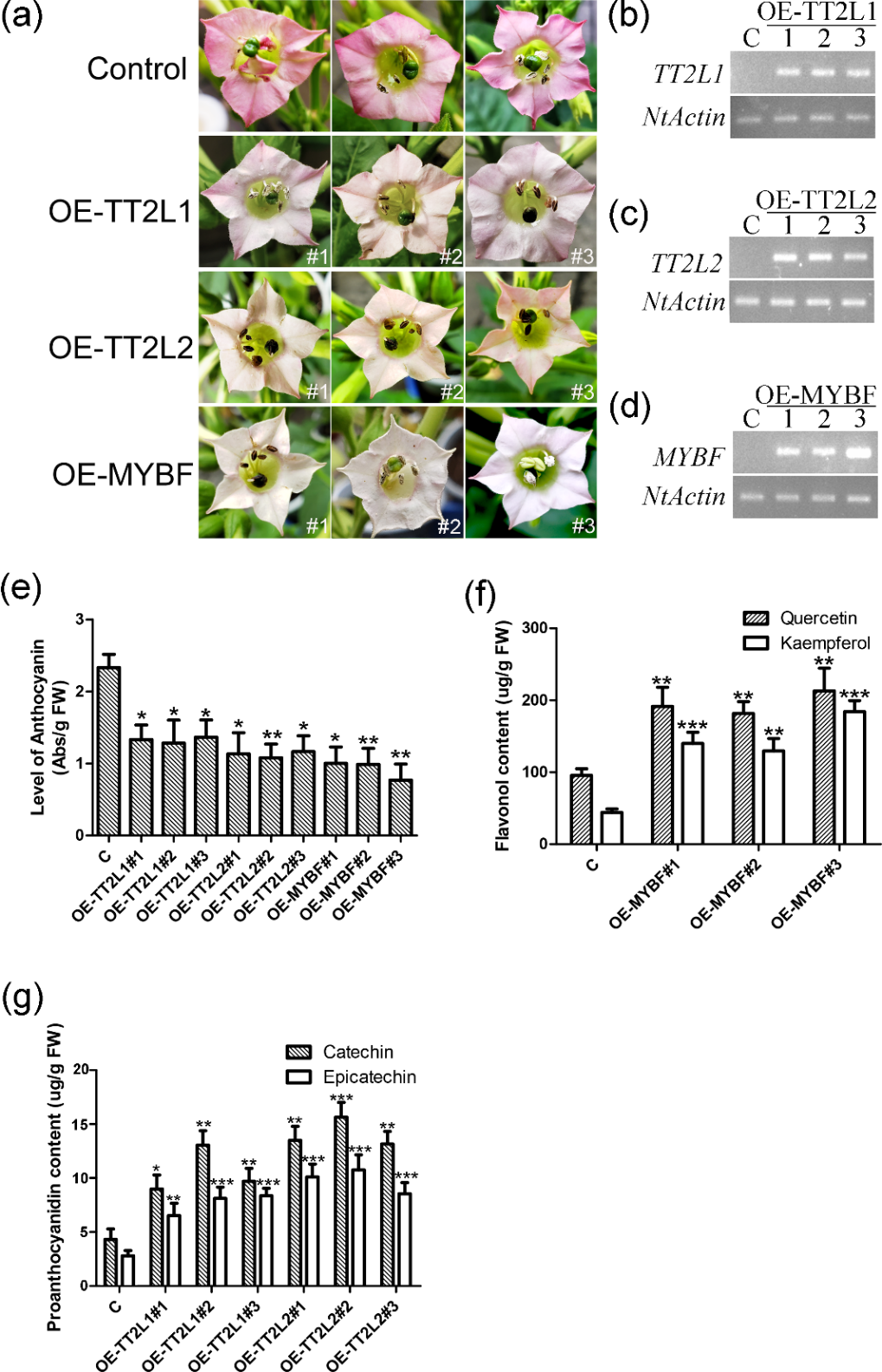


**Supplemental Fig. S8. Phenotypes of transgenic tobacco harboring *TT2L1*, *TT2L2*, and *MYBF* genes. (a)**OE-TT2L1, OE-TT2L2 and OE-MYBF represent transgenic tobacco harboring *TT2L1*, *TT2L2* and *MYBF*, respectively. Characters #1, #2 and #3 represent transgenic lines 1, 2 and 3, respectively. **(b-d)** The positive transgenic lines were determined by semi-quantitative RT-PCR. *NtActin* was used as internal control gene. **(e)** Total anthocyanin levels in control and transgenic tobacco flowers. **(f)** Flavonol contents in control and transgenic tobacco flowers. **(g)** Proanthocyanidin contents in control and transgenic tobacco flowers. Asterisk (*) indicates statistical significance of difference between the experimental and the control groups. (Student’s *t* test, * *P* < 0.05, ** *P* < 0.01, *** *P* < 0.001).


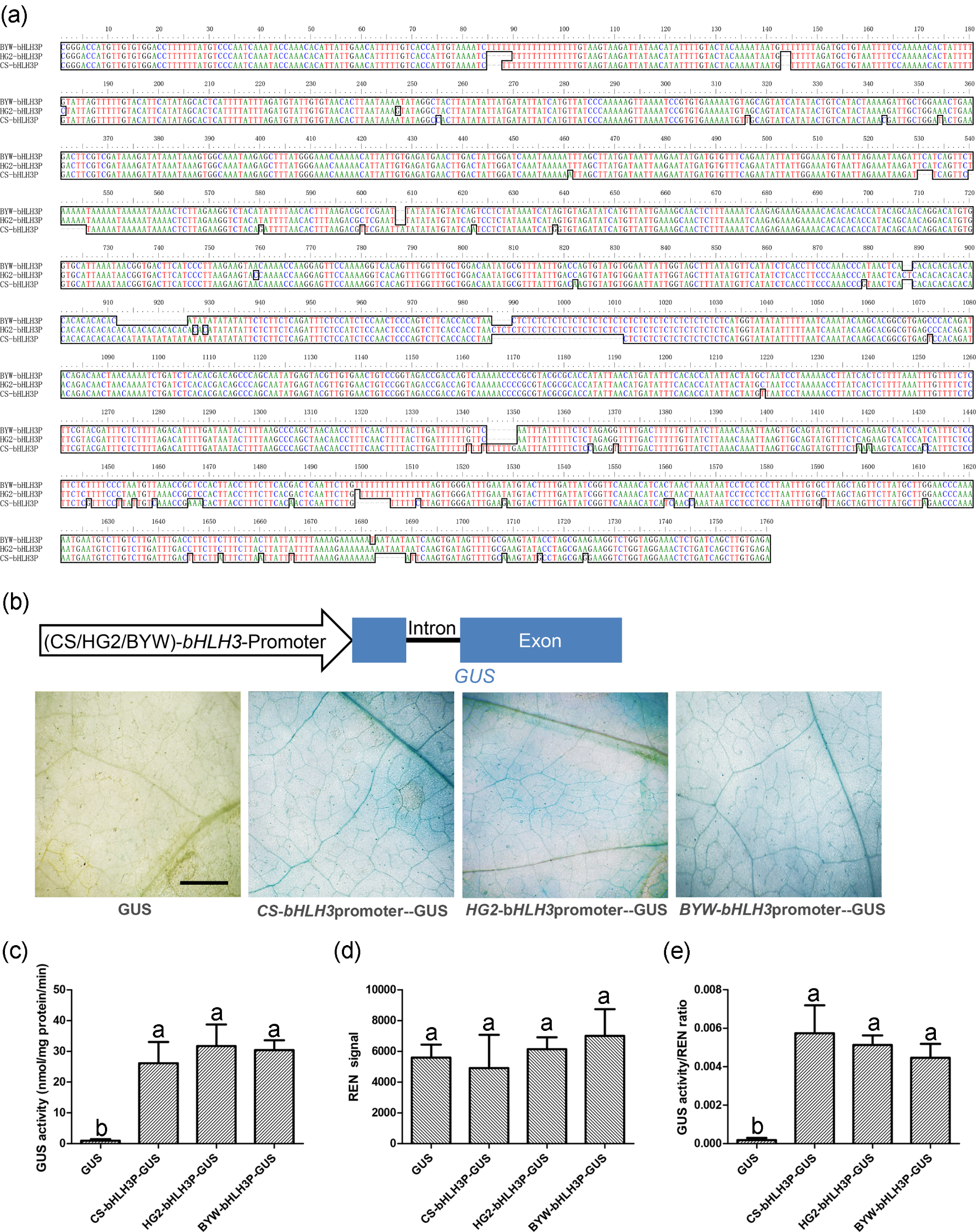


**Supplemental Fig. S9. Comparison of *bHLH3* promoter sequences and activities among HG2, BYW, and CS.** **(a)** Alignment of *bHLH3* promoter sequences of BYW, HG2 and CS. **(b)** GUS was transiently expressed in tobacco leaves transiently transformed with recombinant plasmids and Renilla luciferase at a ratio of 10:1. GUS: GUS without promoter. Bar = 1mm. **(c)** Measurement of GUS activities in tobacco leaves transiently transformed with recombinant plasmids and Renilla luciferase. GUS: GUS without promoter. Data represent averages of 3 experiments. **(d)** Measurement of Renilla luciferase activities in tobacco leaves transiently transformed with recombinant plasmids and Renilla luciferase. GUS: GUS without promoter. Data represent averages of 3 experiments. **(e)** The promoters of *bHLH3* in HG2, BYW, and CS exhibited the same transcriptional activities in tobacco leaves. The signals from the Renilla luciferase was used as internal control to normalise the GUS activity. Significant differences between treatments were determined using one-way ANOVA (*P* < 0.05).


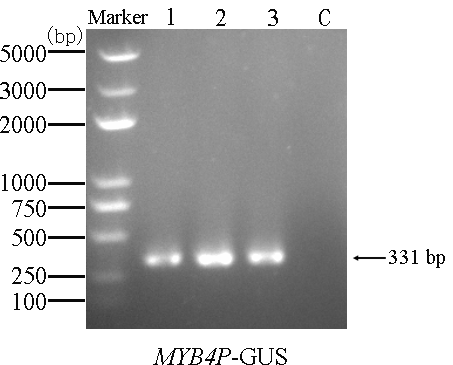


**Supplemental Fig. S10. PCR analyses to detect transgenes in transgenic lines.** PCR analysis of transgene detection in transgenic tobacco expressing *GUS* driven by *MYB4* promoter. C: control. The numbers represent different transgenic lines.

**Reference**

1. Seitz, C., Ameres, S., Schlangen, K., Forkmann, G., & Halbwirth, H. Multiple evolution of flavonoid 3′, 5′-hydroxylase. *Planta*. **242**, 561-573 (2015).
