# Supplementary material for: Abnormal expression of bHLH3 disrupts a flavonoid homeostasis network, causing differences in pigment composition among mulberry fruits
Source: Hortic Res. 2020 Jun 1;7:83. doi: 10.1038/s41438-020-0302-8 (PMC7261776; doi:10.1038/s41438-020-0302-8)
Supplement: Supplementary file 2 — Supplementary Methods [file 41438_2020_302_MOESM2_ESM.docx]

**Methods S1: Measurement of proanthocyanidin and flavonol levels in transgenic tobacco and identification of the products of a cell feeding assay in yeast.**

For PAs quantification, flower powders were soaked in 1 mL of 70% (v/v) acetone containing 0.1% (w/v) ascorbate, and incubated for 24 h in darkness. The extract was centrifuged and the supernatant was transferred to a new 1.5 ml microfuge tube. The extract was subsequently purified by adding equal amount of chloroform, and the supernatant was collected. The solvent was evaporated and the extract was resuspended in 100 µL of 60% (v/v) methanol containing 0.1% (w/v) ascorbic acid. For flavonols quantification, flower powders were immersed into 80% methanol, and sonicated for 30 min and then kept at 4℃ overnight to extract flavonols. Flavonol content in tobacco was calculated as aglycones by preparing acid-hydrolyzed extracts. An aliquot of 400 µL of the supernatant was transferred to a new tube, hydrolyzed by the addition of 120 µL of 3N HCl, incubated at 90 °C for 1 hour, and then extracted with ethyl acetate. The extraction was evaporated to dry powder and then resuspended in 100 µL acetonitrile. The products were filtered through 0.22 µm filter (Millipore, USA) before UPLC analysis. For identification of the products of cell-feeding assay in yeast, the cells were collected by centrifugation at 12,000 g for 2 min after feeding. 2 ml of ethyl acetate was added to the supernatant for extraction. Then the cell pellet was suspended in 500 µL of ethyl acetate, an equal volume of acid-washed glass beads was added, and the cells were vortexed for 30 minutes. Cell extraction was clarified via centrifugation at 12,000 g for 1 min and was mixed with the extraction of the first separated supernatant. The mixed extraction was evaporated and then resuspended in 100 µL of acetonitrile.

An Acquity UPLC system (Waters, Milford, MA, USA) was used to analyze PAs and flavonols contents. Separations were performed on an Acquity UPLC BEH C18 column (1.7 μm, 2.1 × 100 mm). For PAs quantification, acetonitrile and 0.2% (v/v) phosphoric acid were used as mobile phases A and B, respectively, with the following elution proﬁle: 0–5 min, 8–15% B; 5–6 min, 15-20% B; 6-7min, 20-80% B; 7-8min, 80-8% B. Fractions were monitored at 280 nm and samples were eluted at a flow rate of 0.3 ml min^-1^. For flavonols quantification, 40% (v/v) acetonitrile and 0.2% (v/v) phosphoric acid were used as mobile phases A and B, respectively, with the following elution proﬁle: 0–3 min, 20–27% A; 3–6.5 min, 27-84% A; 6.5-7min, 84-20% A. Fractions were monitored at 370 nm and samples were eluted at a flow rate of 0.17 ml min^-1^. Components were identified by comparing the retention times of the eluting peaks with those of commercial standards under the same conditions. Dose-dependent calibration curves of the standards were used to determine the component concentrations.

The products of cell-feeding assay in yeast were separated using acetonitrile and 0.5% (v/v) acetic acid as mobile phases A and B, respectively. The gradient elution conditions were as follows: 0–1 min, 30% A; 1–7 min, 30–80% A; 7–7.5 min, 80%-100% A; 8 min, 30% A. All samples were eluted at a flow rate of 0.1 ml min^-1^. Fractions were monitored at 280 nm, 346 nm, and 370 nm.

**Methods S2: Detailed procedures of LC-MS/MS-based metabolite profiling.**

1. **Chemicals and reagents**

All chemicals and reagents were of analytical grade. Methyl alcohol, acetonitrile, and ethyl alcohol were purchased from Merck Company, Germany (www.merckchemicals.com). Milli-Q system (Millipore Corp., Bedford, MA, USA) ultrapure water was used in this study. Authentic standards were purchasedfrom BioBioPha Co., Ltd (www.biobiopha.com/) and Sigma-Aldrich, USA ([www.sigmaaldrich.com/united-states.html](http://www.sigmaaldrich.com/united-states.html)).

1. **Sample preparation and extraction**

The freeze-dried samples were crushed using a mixer mill (MM 400, Retsch) with a zirconia bead for 1.5min at 30 Hz. The 100 mg powder was weighed and extracted overnight at 4°C with 1.0 ml 70% aqueous methanol containing 0.1 mg/L lidocaine as an internal standard. After being centrifugated at 10000 g for 10 min, the supernatant was filtrated (SCAA-104, 0.22-μm pore size; ANPEL, Shanghai, China, www.anpel.com.cn/) before LC–MS/MS analysis. Quality Control (QC) is a mixer which contains all of the above samples prepared by mixing 20µL of each individual sample. The purpose of constructing QC samples was to test the reproducibility of the whole experiment.

1. **AB Sciex QTRAP4500 (UPLC) analysis**

The compounds extracted were analyzed using an LC-ESI-MS/MS system (UPLC, Shim-pack UFLCSHIMADZU CBM20A, http://www.shimadzu.com.cn/; MS/MS (Applied Biosystems 4500 QTRAP, <http://www.appliedbiosystems.com.cn/>) **^1^**. 5 μl of samples were injected onto a Waters ACQUITY UPLC HSS T3 C18 column (2.1 mm*100 mm, 1.8µm) operating at 40°C and a flow rate of 0.4 mL/min. The mobile phases used were acidified water (0.04% acetic acid) (Phase A) and acidified acetonitrile (0.04% acetic acid) (Phase B). Compounds were separated using the following gradient: 95:5 Phase A/Phase B at 0 min; 5:95 Phase A/Phase B at 11.0 min;5:95 Phase A/Phase B at 12.0 min; 95:5 Phase A/Phase B at 12.1 min; 95:5 Phase A/Phase B at 15.0 min. The effluent was connected to an ESI-triple quadrupole-linear ion trap (QTRAP)–MS.LIT and triple quadrupole (QQQ) scans were acquired on a triple quadrupole-linear ion trap mass spectrometer (QTRAP), AB Sciex QTRAP 4500 System, equipped with an ESI-Turbo Ion-Spray interface, operating in a positive ion mode and controlled by Analyst 1.6.1 software (AB Sciex). The operation parameters were as follows: ESI source temperature 550°C; ion spray voltage (IS) 5500 V; curtain gas(CUR) 25psi; the collision-activated dissociation (CAD) was set highest. QQQ scans were acquired as MRM experiments with optimized declustering potential (DP) and collision energy (CE) for each individual MRM transitions. The m/z range was set between 50 and 1000.

1. **Data pre-processing and metabolites identification**

Data filtering, peak detection, alignment, and calculations were performed using Analyst 1.6.1 software.

- 1. **Data filtering**

To produce a matrix containing fewer biased and redundant data, peaks were checked manually for signal/noise (s/n) > 10 and in-house software written in Perl was used to remove the redundant signals caused by different isotopes, in-source fragmentation, K+, Na+, and NH4+ adduct, and dimerization.

- 1. **Peak detection**

To facilitate the identification/annotation of metabolites, accurate m/z for each Q1 was obtained. Total ion chromatograms (TICs) and Extracted Ion Chromatogram (EICs or XICs) of QC samples were exported to give an overview of metabolite profiles of all samples. The area of each chromatographic peak was calculated.

- 1. **Peak alignment**

Peaks were aligned across the different samples based on spectral pattern and retention time.

- 1. **Metabolites identification**

Metabolites were identified by searching internal database and public databases (MassBank, KNApSAcK, HMDB **^2^**, MoTo DB, and METLIN **^3^**) and comparing the m/z values, the RT, and the fragmentation patterns with the standards.

**5. Multivariate statistical analysis**

**5.1 PCA analysis**

For a preliminary visualization of differences between different groups of samples, the unsupervised dimensionality reduction method principal component analysis (PCA) was applied in all samples using Rpackage models ropls(<http://bioconductor.org/packages/release/bioc/html/ropls.html>). PCA is a statistical procedure that converts hundreds of thousands of correlated metabolites variables into a set of values of linearly uncorrelated variables called principal components.

**5.2 OPLS-DA**

Orthogonal projection to latent structures-discriminant analysis (OPLS-DA). OPLS-DA is an extension of PLS-DA which incorporates an Orthogonal Signal Correction (OSC) filter into a PLS model. The basic concept in OPLS is to separate the systematic variation in X into two parts, one that is correlated to Y and one that is not correlated (orthogonal) with Y. Only the Y‐predictive variation is used to model the data. OPLS-DA was applied in comparison groups using R package models ropls. The OPLS-DA model was further validated by cross-validation and 200 permutation test **^4^**. For cross-validation, the data was partitioned into seven subsets, where each of the subsets was then used as a validation set. R2 indicated the total variation in the data matrix that was explained by the model. Predictive ability (Q2) values represented the most recognized diagnostic statistical parameter to validate the OPLS-DA model in metabolomics. Acceptable predictive model is considered for Q2 value greater than 0.4. Good predictive model is considered for Q2 value greater than 0.9. Permutation test randomly permutes class labels for200 times and then produces a distribution of R2’ values and Q2’ values. In essence, a reliable model should yield significantly larger R2 and Q2 value compared to R2’ and Q2’ values generated from random models using the same data set.

**5.4 Loadings plot**

Loadings from (O)PLS are the directions of projection with respect to the original variables. Variables whose loadings were far-away from the origin in a loadings plot might be inferred to have the greatest contribution to class separation.

1. **Differential metabolites analysis**

A variable importance in projection (VIP) score of (O)PLS model was applied to rank the metabolites that best distinguished between two groups. The threshold of VIP was set to 1. In addition, T-test was also used as a univariate analysis for screening differential metabolites. Those with a P value of T test < 0.05 and VIP ≥ 1 were considered differential metabolites between two groups.

**Reference**

1. **Chen, W. et al.** A novel integrated method for large-scale detection, identification, and quantification of widely targeted metabolites: application in the study of rice metabolomics. *Molecular Plant*. **6**, 1769-1780 (2013).
2. **Wishart, D. S. et al.** HMDB 3.0—the human metabolome database in 2013. *Nucleic Acids Research.* **41**, D801-D807 (2013).
3. **Zhu, ZJ. et al.** Liquid chromatography quadrupole time-of-flight mass spectrometry characterization of metabolites guided by the METLIN database. *Nature Protocols.* **8**, 451-460 (2013).
4. **Westerhuis, JA. et al.** Assessment of PLSDA cross validation. *Metabolomics*. **4**, 81-89 (2008).
