# Supplementary material for: Abnormal expression of bHLH3 disrupts a flavonoid homeostasis network, causing differences in pigment composition among mulberry fruits
Source: Hortic Res. 2020 Jun 1;7:83. doi: 10.1038/s41438-020-0302-8 (PMC7261776; doi:10.1038/s41438-020-0302-8)
Supplement: Supplementary file 3 — Supplementary Tables [file 41438_2020_302_MOESM3_ESM.pdf]

Supplementary Table S1. Qualitative and quantitative results for metabolites of CS, HG2, and BYW.

Index	Ion mode	Q1 (Da)	Q3 (Da)	Rt (min)	Molecular Weight (Da)	Ionization model	Compounds	Class	CS-1	CS-2	CS-3	HG2-1	HG2-2	HG2-3	BYW-1	BYW-2	BYW-3	QC1	QC2	QC3
Mul0052	Positive	162.1	98.3	0.81	161.1	[M+H]+	2-Aminoadipic acid (L-Homoglutamic acid)	Amino acids	377000	594000	846000	381000	614000	421000	274000	225000	328000	325000	384000	397000
Mul0057	Positive	136	89.9	0.81	135.035	[M+H]+	DL-homocysteine	Amino acids	9	9	3300	8220	10500	9050	5900	7240	9300	3400	9150	6450
Mul0064	Positive	118	88	0.85	117.079	[M+H]+	Dl-Norvaline	Amino acids	2830000	2710000	6510000	5340000	7460000	4350000	16300000	16300000	18500000	8060000	9120000	10200000
Mul0412	Negative	180.1	163	1.21	181.074	[M-H]-	L-(-)-Tyrosine	Amino acids	37100	16000	93100	60700	244000	67200	1320000	1400000	1300000	453000	399000	407000
Mul0018	Positive	175	69.9	0.69	174.1117	[M+H]+	L-(+)-Arginine	Amino acids	692000	481000	1690000	1100000	1420000	1060000	2630000	1770000	1690000	1130000	1300000	1300000
Mul0013	Positive	147	83.2	0.67	146.1055	[M+H]+	L-(+)-Lysine	Amino acids	3150000	4600000	8360000	3760000	7290000	2060000	6730000	5310000	6660000	5150000	6330000	4910000
Mul0011	Positive	133	70	0.65	132.16	[M+H]+	L(+)-Ornithine	Amino acids	156000	146000	174000	141000	141000	72800	472000	273000	292000	254000	304000	323000
Mul0029	Positive	90.048	71.8	0.76	89.048	[M+H]+	L-Alanine	Amino acids	420000	308000	666000	296000	518000	156000	636000	619000	702000	527000	490000	520000
Mul0367	Negative	131.054	71.8	0.75	132.054	[M-H]-	L-Asparagine	Amino acids	6860000	3090000	9880000	3240000	4250000	1690000	12800000	12000000	12600000	11000000	12400000	9560000
Mul0374	Negative	132	99.9	0.77	133.0375	[M-H]-	L-Aspartic acid	Amino acids	721000	346000	1680000	1710000	2800000	1410000	5540000	6240000	5690000	4130000	3600000	3920000
Mul0028	Positive	176.1	98.9	0.76	175.0957	[M+H]+	L-Citrulline	Amino acids	223000	251000	256000	239000	326000	246000	377000	295000	349000	290000	312000	312000
Mul0375	Negative	146.1	102.1	0.77	147.0532	[M-H]-	L-Glutamic acid	Amino acids	10300000	8470000	14600000	5220000	8030000	4720000	5290000	5900000	6090000	7080000	6090000	7160000
Mul0027	Positive	147.069	83.7	0.75	146.069	[M+H]+	L-Glutamine	Amino acids	5470000	6640000	13500000	5340000	11400000	2870000	13900000	10300000	12900000	9200000	10500000	11000000
Mul0360	Negative	154.1	92.9	0.67	155.0695	[M-H]-	L-Histidine	Amino acids	74600	24400	122000	137000	155000	90400	145000	176000	201000	126000	118000	118000
Mul0040	Positive	190.1	84	0.78	189.1113	[M+H]+	L-Homocitrulline	Amino acids	34300	32600	32900	70000	85700	73500	116000	88600	83400	75900	64700	81100
Mul0060	Positive	269	134	0.81	268.055	[M+H]+	L-Homocystine	Amino acids	170000	65100	186000	43700	47100	59200	45800	47800	84400	48300	53600	60300
Mul0366	Negative	118.1	101	0.75	119.0582	[M-H]-	L-Homoserine	Amino acids	21800	8700	32600	9110	15200	9860	20100	18100	17500	14400	23900	25100
Mul0092	Positive	132	85.9	1.23	131.0946	[M+H]+	L-Isoleucine	Amino acids	15800000	15800000	30300000	32500000	36300000	31600000	45200000	45600000	43500000	38700000	41000000	39800000
Mul0097	Positive	132.1	86	1.27	131.0946	[M+H]+	L-Leucine	Amino acids	16400000	15900000	30300000	35300000	37700000	33200000	45900000	45800000	44800000	39800000	39600000	39200000
Mul0078	Positive	150	72.8	1.18	149.051	[M+H]+	L-Methionine	Amino acids	505000	514000	819000	1900000	4790000	1630000	4430000	5900000	5570000	2900000	3400000	3440000
Mul0121	Positive	166	120.1	1.96	165.079	[M+H]+	L-Phenylalanine	Amino acids	8090000	6590000	8240000	5420000	5620000	5070000	4300000	4350000	3880000	5270000	4630000	4720000
Mul0043	Positive	116.1	70	0.79	115.0633	[M+H]+	L-Proline	Amino acids	21100000	19400000	17400000	1960000	3740000	1190000	4540000	13500000	16800000	9550000	10300000	11200000
Mul0363	Negative	104	73.9	0.75	105.0426	[M-H]-	L-Serine	Amino acids	964000	754000	1100000	366000	589000	202000	390000	463000	590000	614000	568000	492000
Mul0087	Positive	175	158.1	1.21	174.1	[M+H]+	L-Theanine	Amino acids	16000	16600	8350	9	6420	9710	12200	10200	12100	11300	11200	11700
Mul0364	Negative	118	100.9	0.75	119.0582	[M-H]-	L-Threonine	Amino acids	221000	136000	549000	223000	396000	158000	240000	299000	285000	296000	282000	230000
Mul0138	Positive	205.1	146.1	2.36	204.09	[M+H]+	L-Tryptophan	Amino acids	16600000	7170000	24500000	21600000	21600000	20700000	21800000	20600000	21700000	23500000	23300000	22700000
Mul0095	Positive	138.084	103	1.25	137.084	[M+H]+	L-Tyramine	Amino acids	2750	7220	9	117000	71200	186000	11300	9	9	29800	43600	40900
Mul0056	Positive	118.1	71.8	0.81	117.079	[M+H]+	L-Valine	Amino acids	2710000	2820000	6770000	5530000	8200000	4870000	17400000	17900000	19600000	9020000	9580000	10900000
Mul0068	Positive	219	156.1	1.11	218.09	[M+H]+	(5-L-Glutamyl)-L-amino acid	Amino acid derivatives	217000	184000	231000	203000	246000	190000	157000	150000	182000	204000	189000	211000
Mul0015	Positive	170.1	124.1	0.68	169.0851	[M+H]+	1-Methylhistidine	Amino acid derivatives	68500	41300	69400	33400	40900	45500	44900	47100	42000	43700	34100	39200
Mul0466	Negative	145.1	101	2.46	146.0579	[M-H]-	2,3-dimethylsuccinic acid	Amino acid derivatives	396000	318000	345000	203000	192000	184000	519000	526000	496000	294000	279000	255000
Mul0024	Positive	191.095	127.7	0.73	190.095	[M+H]+	2,6-Diaminooimelic acid	Amino acid derivatives	114000	103000	141000	88400	126000	76900	84700	70600	114000	103000	107000	101000
Mul0031	Positive	104	85.9	0.76	103.063	[M+H]+	2-Aminoisobutyric acid	Amino acid derivatives	1220000	1290000	983000	438000	474000	415000	1730000	1280000	1470000	1070000	926000	1020000
Mul0090	Positive	198.2	114.9	1.21	197.069	[M+H]+	3,4-Dihydroxy-DL-phenylalanine	Amino acid derivatives	32500	31500	9	40100	57100	54700	27800	57100	46900	31300	39600	36800
Mul0433	Negative	161	56.8	1.44	162.053	[M-H]-	3-Hydroxy-3-methylpentane-1,5-dioic acid	Amino acid derivatives	5190000	2610000	1780000	802000	861000	850000	1130000	992000	1150000	2040000	1830000	1720000
Mul0014	Positive	170	96	0.67	169.1811	[M+H]+	3-N-Methyl-L-histidine	Amino acid derivatives	4110	3840	9080	6780	5070	6570	21500	15900	22000	9230	8880	14000
Mul0392	Negative	162	144.1	0.83	163.1287	[M-H]-	4-Hydroxy-L-glutamic acid	Amino acid derivatives	24100	15600	14400	12600	12100	6790	16500	20000	20100	18600	16800	12800
Mul0026	Positive	118.2	101	0.75	117.079	[M+H]+	5-Aminovaleric acid	Amino acid derivatives	9	7710	6900	2450	9	7200	4790	5680	10600	3250	5560	6010
Mul0109	Positive	221.1	114.8	1.63	220.085	[M+H]+	5-Hydroxy-L-tryptophan	Amino acid derivatives	24200	19200	34200	65500	40500	55200	66900	81200	60800	46300	49200	43100
Mul0094	Positive	130	83.9	1.24	129.043	[M+H]+	5-oxoproline	Amino acid derivatives	9230000	16000000	11200000	3830000	2920000	3620000	26900000	23400000	22900000	14100000	14400000	17200000
Mul0526	Negative	245	203.2	3.81	246	[M-H]-	Acetyl tryptophan	Amino acid derivatives	16400	16400	28400	61100	80400	110000	112000	113000	99700	56300	61600	69500
Mul0393	Negative	144.1	126.1	0.83	145.074	[M-H]-	Allysine(6-Oxo DL-Norleucine)	Amino acid derivatives	52400	26900	32600	85100	60800	141000	66900	69100	69600	81600	79200	65500
Mul0053	Positive	458.1	116.4	0.81	457.1	[M+H]+	Aspartic acid di-O-glucoside	Amino acid derivatives	386000	392000	1630000	92100	173000	59400	348000	1090000	908000	635000	585000	604000
Mul0142	Positive	281.3	120	2.4	280.1059	[M+H]+	Asp-phe	Amino acid derivatives	203000	132000	209000	52800	44000	49800	81600	64100	53600	85200	70400	77300
Mul0042	Positive	179	75.9	0.78	178.041	[M+H]+	CYS-GLY	Amino acid derivatives	21300	26100	12000	12500	22800	17800	9	9	8220	9840	15300	18400
Mul0361	Negative	159.2	88	0.74	160.0848	[M-H]-	D-Alanyl-D-Alanine	Amino acid derivatives	15800	8520	10400	4300	3800	4630	2750	3050	5840	6090	4450	4510
Mul0081	Positive	613.152	355.5	1.2	612.152	[M+H]+	Glutathione oxidized	Amino acid derivatives	23500	15500	10100	16800	21900	14500	14700	21900	20900	24500	25400	24900
Mul0077	Positive	308	162	1.18	307.084	[M+H]+	Glutathione reduced form	Amino acid derivatives	6010	9	9	3510000	2480000	3130000	4310	9590	73100	338000	370000	342000
Mul0044	Positive	173.2	116.1	0.79	172.0848	[M+H]+	Glycyl-L-proline	Amino acid derivatives	53500	42800	31500	10100	13200	6590	13500	23300	39500	21800	21200	25700
Mul0017	Positive	189	144.2	0.68	188.127	[M+H]+	H-HomoArg-OH	Amino acid derivatives	46000	42600	37200	177000	196000	150000	90600	69100	80900	99000	96900	86800
Mul0005	Positive	112.08	94.6	0.59	111.08	[M+H]+	Histamine	Amino acid derivatives	136000	135000	301000	47300	42100	45700	25200	27800	32400	51600	71000	80800
Mul0383	Negative	308.1	128.2	0.8	309.1	[M-H]-	L-Glutamic acid O-glucoside	Amino acid derivatives	157000	110000	101000	39700	78800	28400	12500	20300	43000	47000	46500	44100
Mul0382	Negative	307	145.2	0.79	308	[M-H]-	L-Glutamine O-hexside	Amino acid derivatives	315000	202000	157000	59800	126000	23200	48700	140000	226000	156000	121000	105000
Mul0232	Positive	448.1	215.7	3.99	447.1	[M+H]+	L-Glutaminyl-L-valyl-L-valyl-L-cysteine	Amino acid derivatives	9	9	9	14000	12500	19900	28600	35800	12900	21100	16100	14000
Mul0122	Positive	209.1	146.1	1.96	208.0848	[M+H]+	L-Kynurenine	Amino acid derivatives	63900	57300	52700	259000	214000	267000	174000	163000	114000	162000	189000	181000
Mul0114	Positive	164	61	1.77	163	[M+H]+	L-Methionine methyl ester	Amino acid derivatives	14400	15500	25400	14400	23300	14300	35000	74200	72100	27000	31800	33800
Mul0401	Negative	128	126	1.15	129.079	[M-H]-	L-Pipecolic acid	Amino acid derivatives	9460	9	8390	9	9	9	13700	24000	21000	9080	10400	9860
Mul0037	Positive	277	84	0.77	276.132	[M+H]+	L-Saccharopine	Amino acid derivatives	238000	322000	299000	309000	297000	313000	229000	219000	202000	313000	288000	276000
Mul0055	Positive	235.1	118.4	0.81	234.1	[M+H]+	Lysine butyrate	Amino acid derivatives	11200	32600	5360	3550	3570	9	16100	14900	17900	3150	2490	6020
Mul0046	Positive	166	149.1	0.79	165.046	[M+H]+	Methionine sulfoxide	Amino acid derivatives	453000	644000	665000	428000	437000	469000	430000	362000	423000	386000	449000	485000

Mul0236	Positive	247.1	130.1	4.02	246.1	[M+H]+	N-(3-Indolylacetyl)-L-alanine	Amino acid derivatives	26300	19700	18000	11100	25500	15700	33600	31200	36000	22300	18100	27400
Mul0041	Positive	104	58	0.78	103.063	[M+H]+	N,N-Dimethylglycine	Amino acid derivatives	338000	336000	403000	93100	123000	49700	374000	404000	465000	275000	231000	256000
Mul0085	Positive	189.2	84	1.21	188.1161	[M+H]+	N6-Acetyl-L-lysine	Amino acid derivatives	62000	72900	21100	180000	273000	200000	337000	252000	221000	137000	152000	158000
Mul0082	Positive	176	88	1.2	175.048	[M+H]+	N-Acetylaspartate	Amino acid derivatives	9	9	9	576000	173000	187000	23400	13000	26900	85200	82600	79800
Mul0417	Negative	188.1	102	1.23	189.0637	[M-H]-	N-Acetyl-L-glutamic acid	Amino acid derivatives	140000	74900	11200	9	13700	6520	24400	23800	18000	34200	39700	38900
Mul0199	Positive	174.105	86	3.49	173.105	[M+H]+	N-Acetyl-l-leucine	Amino acid derivatives	28700	23300	5810	9	6450	9	9	9	8520	9870	11000	10700
Mul0469	Negative	222.1	163	2.51	223.085	[M-H]-	N-Acetyl-L-tyrosine	Amino acid derivatives	82200	73000	9	2950	6290	9	9	9	11300	16700	17600	17200
Mul0465	Negative	190.1	84	2.46	191.1	[M-H]-	N-Acetylmethionine	Amino acid derivatives	27600	15400	18600	2420	2700	9	7220	9500	12800	8100	10200	7210
Mul0089	Positive	162	74	1.21	161.069	[M+H]+	N-Acetylthreonine	Amino acid derivatives	21200	29100	18900	58300	24800	58700	11400	13400	9	19900	21300	19400
Mul0126	Positive	237.1	146.1	2.01	236.08	[M+H]+	N'-Formylkynurenine	Amino acid derivatives	6920	12400	21900	13000	21300	13600	75900	84400	68900	39200	36000	39600
Mul0441	Negative	187.1	130.1	1.91	188.1161	[M-H]-	N-Glycyl-L-leucine	Amino acid derivatives	45000	12800	23500	4840	7250	2930	18300	32400	54800	20200	17100	20000
Mul0100	Positive	132.1	72.8	1.29	131.0582	[M+H]+	N-Propionylglycine	Amino acid derivatives	21200	13600	39800	40800	57700	43900	147000	129000	147000	74400	79600	89900
Mul0069	Positive	217.1	158.1	1.16	216.1222	[M+H]+	Nα-Acetyl-L-arginine	Amino acid derivatives	157000	97600	43600	32100	23500	27100	37800	23500	31500	57600	52700	47700
Mul0086	Positive	189.2	130	1.21	188.0797	[M+H]+	Nα-Acetyl-L-glutamine	Amino acid derivatives	240000	155000	39700	240000	258000	196000	298000	329000	233000	213000	199000	180000
Mul0525	Negative	263.1	189	3.78	264.111	[M-H]-	N-γ-Acetyl-N-2-Formyl-5-methoxykynurenamine	Amino acid derivatives	5280	6030	8820	21400	23200	21800	64400	39300	40200	23500	24300	26600
Mul0397	Negative	184.1	78.8	0.99	185.009	[M-H]-	O-Phospho-L-serine	Amino acid derivatives	9	9	4780	48800	32100	35800	19100	14000	19800	20100	18200	15500
Mul0174	Positive	265.1	130.1	3.02	264.111	[M+H]+	Phenylacetyl-L-glutamine	Amino acid derivatives	8050	5370	13600	8060	9540	13200	25900	17100	20300	14700	14100	11500
Mul0505	Negative	311.4	147.1	3.34	312.147	[M-H]-	Phe-Phe	Amino acid derivatives	409000	312000	313000	7300	5590	6410	5190	4170	6460	124000	112000	125000
Mul0426	Negative	383	134	1.31	384.122	[M-H]-	S-(5'-Adenosy)-L-homocysteine	Amino acid derivatives	77000	68700	90200	11500	37300	12100	30200	29200	39500	39900	35600	30200
Mul0020	Positive	399.2	250.1	0.71	398.137	[M+H]+	S-(5'-Adenosyl)-L-methionine	Amino acid derivatives	67400	205000	126000	187000	266000	192000	300000	293000	330000	163000	243000	239000
Mul0093	Positive	322.4	176.1	1.24	321.1	[M+H]+	S-(methyl)glutathione	Amino acid derivatives	71600	62700	65800	99700	40300	61600	18800	19300	27300	124000	101000	127000
Mul0491	Negative	153	109	2.98	154.027	[M-H]-	2,4-Dihydroxybenzoic acid	Benzoic acid derivatives	99100	104000	85500	137000	145000	145000	223000	138000	319000	72100	60700	45900
Mul0443	Negative	315.1	152.1	1.93	316.1	[M-H]-	2,5-dihydroxy benzoic acid O-hexside	Benzoic acid derivatives	13600000	9480000	19600000	6150000	8770000	6080000	4660000	2520000	2840000	11100000	9140000	9730000
Mul0481	Negative	153	108.1	2.79	154.027	[M-H]-	2,5-dihydroxybenzoic acid (Gentisic acid)	Benzoic acid derivatives	51900	44200	24500	40900	130000	44700	74800	79500	99300	244000	84200	161000
Mul0511	Negative	121.04	91.9	3.58	122.037	[M-H]-	4-Hydroxybenzaldehyde	Benzoic acid derivatives	5860000	2850000	4590000	397000	277000	379000	407000	337000	322000	1930000	2060000	2020000
Mul0251	Positive	457.2	191.5	4.44	456.2	[M+H]+	8-Methyl-2-oxo-4-phenyl-2H-chromen-7-yl 4-(hexyloxy)benzoate	Benzoic acid derivatives	219000	171000	140000	86400	76900	75100	139000	111000	85000	105000	119000	136000
Mul0378	Negative	460.1	118.2	0.78	461.1	[M-H]-	Anthranilate O-hexosyl-O-hexoside	Benzoic acid derivatives	76700	28100	310000	74600	97600	60300	159000	174000	171000	145000	127000	128000
Mul0551	Negative	121.0368	77	4.37	122.0368	[M-H]-	Benzoic acid	Benzoic acid derivatives	6900000	7960000	793000	1020000	570000	836000	1020000	1120000	2660000	3130000	2940000	2830000
Mul0558	Negative	211.07	137.1	4.44	212.069	[M-H]-	Eudesmic acid (3,4,5-trimethoxybenzoic acid)	Benzoic acid derivatives	21400	19100	16900	4330	9	9	9	9	9	9130	8520	8960
Mul0449	Negative	331	313.7	2.14	332	[M-H]-	Gallic acid O-Hexoside	Benzoic acid derivatives	823000	690000	627000	253000	228000	251000	499000	381000	455000	522000	493000	494000
Mul0177	Positive	137.3	94.3	3.09	136.3	[M+H]+	p-Aminobenzoate	Benzoic acid derivatives	51600	93600	92400	129000	140000	205000	148000	160000	144000	105000	89000	117000
Mul0458	Negative	359.1	197.1	2.31	360.1	[M-H]-	Syringic acid O-glucoside	Benzoic acid derivatives	26000	18900	16100	48400	34800	48400	62700	42900	35400	41300	53800	37900
Mul0527	Negative	151.047	136.1	3.85	152.0473	[M-H]-	Vanillin	Benzoic acid derivatives	312000	214000	279000	700000	380000	720000	508000	554000	429000	494000	480000	456000
Mul0106	Positive	153.06	136.1	1.5	152.059	[M+H]+	1,4-dihydro-1-Methyl-4-oxo-3-pyridinecarboxamide	Pyridine derivatives	122000	219000	109000	44200	40200	45400	41800	31600	43500	55600	68000	67200
Mul0111	Positive	184.05	148.1	1.66	183.053	[M+H]+	4-Pyridoxic acid	Pyridine derivatives	70400	71600	89000	88500	78300	80700	61800	62500	57100	73000	73700	77000
Mul0391	Negative	163.2	162.9	0.83	164.069	[M-H]-	1,5-Anhydro-D-glucitol	Alcohols and polyols	43400	41400	25100	18700	24800	19900	17300	11500	28000	27200	24300	20700
Mul0038	Positive	183	59.3	0.77	182.079	[M+H]+	D-Sorbitol	Alcohols and polyols	1670000	1920000	2370000	1570000	1500000	1950000	1630000	1570000	2020000	1680000	1760000	1870000
Mul0369	Negative	181.08	70.8	0.76	182.079	[M-H]-	Dulcitol	Alcohols and polyols	25600	22000	25200	14700	15800	18100	14500	11400	45400	26100	25400	22400
Mul0131	Positive	206.13	188.2	2.22	205.131	[M+H]+	Pantothenol	Alcohols and polyols	17900	13700	15600	36600	28700	40700	28700	45400	33100	29100	26700	29500
Mul0036	Positive	147.12	88	0.77	146.1181	[M+H]+	Acetylcholine	Cholines	249000	509000	289000	1150000	516000	1140000	54700	35600	68100	296000	362000	387000
Mul0039	Positive	104.1	60.2	0.78	103.1	[M+H]+	Choline	Cholines	10200000	10500000	7300000	3860000	4000000	4100000	13400000	10500000	11100000	8550000	8010000	9250000
Mul0158	Positive	280	178.4	2.79	280	Protonated	Feruloylcholine	Cholines	15700	6480	10600	9	9	9	15300	66000	50700	8040	6040	6550
Mul0033	Positive	184	83.2	0.77	183	[M+H]+	O-Phosphocholine	Cholines	178000	171000	104000	488000	680000	476000	487000	493000	506000	461000	424000	428000
Mul0048	Positive	258.2	125.2	0.8	258.2	Protonated	sn-Glycero-3-phosphocholine	Cholines	1810000	1720000	3060000	347000	650000	291000	325000	271000	456000	839000	760000	792000
Mul0459	Negative	305	125	2.36	306.074	[M-H]-	(+)-Gallocatechin (GC)	Catechin derivatives	9	9	9	100000	192000	88300	9	9	7680	77600	72800	76100
Mul0483	Negative	289.079	125	2.81	290.079	[M-H]-	Catechin	Catechin derivatives	5590	5230	9	311000	322000	364000	336000	801000	484000	163000	160000	159000
Mul0521	Negative	441.3	289.1	3.73	442.3	[M-H]-	Epicatechin gallate (ECG)	Catechin derivatives	77000	44800	38700	71100	96400	117000	67100	61300	70000	33400	67800	49500
Mul0500	Negative	457.2	169.1	3.18	458.085	[M-H]-	Epigallate catechin gallate (EGCG)	Catechin derivatives	591000	242000	424000	460000	420000	616000	361000	333000	401000	204000	447000	318000
Mul0151	Positive	307	139.1	2.64	306	[M+H]+	Epigallocatechin (EGC)	Catechin derivatives	62300	38200	53600	75600	88400	95700	36900	35700	33500	54400	53700	62300
Mul0512	Negative	593.1	549.4	3.59	594.1	[M-H]-	Gallocatechin-catechin	Catechin derivatives	9	9	9	280000	248000	305000	21700	42200	39000	50000	68000	62100
Mul0140	Positive	611.2	611.1	2.39	610.2	[M+H]+	Gallocatechin-gallocatechin	Catechin derivatives	9	9	9	8590000	15500000	16200000	63600	77400	134000	4150000	5210000	5580000
Mul0497	Negative	289	78.8	3.17	290.3	[M-H]-	L-Epicatechin	Catechin derivatives	9	9	9	156000	178000	121000	27600	39100	41100	46100	57400	56100
Mul0472	Negative	153.1	109.1	2.55	154.027	[M-H]-	Protocatechuic acid	Catechin derivatives	196000	212000	90700	246000	388000	250000	228000	304000	362000	924000	429000	565000
Mul0471	Negative	315.1	153.2	2.55	316.1	[M-H]-	Protocatechuic acid O-glucoside	Catechin derivatives	741000	592000	773000	1630000	2000000	1790000	2470000	2470000	1860000	1620000	1460000	1540000
Mul0172	Positive	139.1	93	2.99	138.032	[M+H]+	Protocatechuic aldehyde	Catechin derivatives	194000	185000	106000	41400	57300	70600	64700	18100	29900	77100	90900	94800
Mul0010	Positive	131.1	72.1	0.64	130.1	[M+H]+	Agmatine	Phenolamides	27200	55000	14200	53500	52200	50700	32300	28600	16400	29300	32300	30200
Mul0192	Positive	438.239	147.5	3.37	437.239	[M+H]+	N', N''-di-p-coumaroylspermidine	Phenolamides	53100	38200	11600	9	9	9	9	9	9	11700	10200	14800

Mul0200	Positive	558.282	264.8	3.5	557.282	[M+H]+	N', N"-disinapoylspermidine	Phenolamides	3600000	3670000	5330000	372000	336000	601000	279000	316000	161000	2330000	1900000	1320000
Mul0143	Positive	584.2	325.8	2.41	583.2	[M+H]+	N',N",N"'-p-coumaroyl-cinnamoyl-caffeoyl spermidine	Phenolamides	50900	50100	60900	1860	4350	2490	16100	15800	15500	6830	8830	9430
Mul0059	Positive	131	71.9	0.81	130.11061	[M+H]+	N-Acetylputrescine	Phenolamides	25700	53100	8570	42700	53200	51100	27100	22800	19500	22200	24800	40800
Mul0161	Positive	309	147.5	2.83	308	[M+H]+	N-sinapoyl cadaverine	Phenolamides	16500	5360	12300	9	9	9	9	9	9	3900	5780	5690
Mul0006	Positive	89	71.9	0.6	88.1	[M+H]+	Putrescine	Phenolamides	48400	29400	152000	800000	569000	600000	939000	757000	550000	499000	416000	504000
Mul0002	Positive	146.2	72.1	0.56	145.2	[M+H]+	Spermidine	Phenolamides	5580	6300	4640	6530	5140	9410	16300	12700	10400	7470	10400	11500
Mul0001	Positive	203	112	0.54	202	[M+H]+	Spermine	Phenolamides	12000000	9970000	14100000	9930000	11200000	10600000	8940000	8540000	8400000	9850000	9660000	10800000
Mul0032	Positive	150	133.1	0.76	149.07	[M+H]+	1-Methyladenine	Nucleotide and its derivates	7910	10400	12600	41500	88000	40800	98900	86800	104000	44500	69100	62500
Mul0136	Positive	282.1	150.1	2.31	281.112	[M+H]+	1-Methyladenosine	Nucleotide and its derivates	58500	39200	22900	9	9	9	3930	4090	9	14000	11400	15800
Mul0021	Positive	74	56.9	0.71	73.0971	[M+H]+	1-methylguanidine	Nucleotide and its derivates	10700	4110	3760	11200	12600	8280	30100	15100	4650	10500	10600	10300
Mul0134	Positive	312	180.1	2.23	311.123	[M+H]+	2-(dimethylamino)guanosine	Nucleotide and its derivates	164000	96500	101000	45900	39800	31200	165000	162000	116000	90900	95900	106000
Mul0098	Positive	332	136.1	1.27	331.068	[M+H]+	2'-Deoxyadenosine-5'-monophosphate	Nucleotide and its derivates	157000	145000	163000	195000	140000	129000	90500	90300	73500	124000	94500	132000
Mul0022	Positive	333.052	137	0.72	332.052	[M+H]+	2'-Deoxyinosine-5'-monophosphate	Nucleotide and its derivates	680000	786000	130000	288000	432000	625000	101000	164000	167000	271000	221000	212000
Mul0034	Positive	152.1	135.1	0.77	151.0494	[M+H]+	2-Hydroxy-6-aminopurine	Nucleotide and its derivates	171000	258000	188000	58600	146000	62500	143000	157000	151000	130000	129000	133000
Mul0155	Positive	298	135.1	2.73	297.09	[M+H]+	5'-Deoxy-5'-(methylthio)adenosine	Nucleotide and its derivates	480000	376000	1140000	1020000	1230000	870000	2840000	1850000	2860000	1560000	1150000	1290000
Mul0115	Positive	259.085	126.7	1.8	258.085	[M+H]+	5-Methyluridine	Nucleotide and its derivates	112000	82100	58900	8800	9	6330	12900	10700	14900	36400	38000	35200
Mul0120	Positive	167.1	121	1.95	166.1	[M+H]+	6-Methylmercaptopurine	Nucleotide and its derivates	7600000	9550000	8170000	15100000	16600000	14800000	24400000	25900000	26300000	17800000	18800000	18400000
Mul0119	Positive	284	168.1	1.91	283.092	[M+H]+	8-Hydroxy-2-deoxyguanosine	Nucleotide and its derivates	3560	9	9	5000	4930	2990	8080	7950	8730	5790	5670	5600
Mul0105	Positive	300.1	168.1	1.48	299.087	[M+H]+	8-Hydroxyguanosine	Nucleotide and its derivates	75000	33800	41100	27000	24300	28300	23700	25400	23000	33600	29000	31900
Mul0084	Positive	136.1	136.1	1.21	135.0545	[M+H]+	Adenine	Nucleotide and its derivates	14500000	17200000	13300000	704000	1210000	815000	3740000	4540000	4740000	5900000	6560000	6780000
Mul0117	Positive	268.097	136.1	1.89	267.097	[M+H]+	Adenosine	Nucleotide and its derivates	24600000	24100000	25200000	22200000	26100000	20200000	20300000	21500000	21700000	25800000	26100000	26900000
Mul0065	Positive	348.1	136.4	0.88	347.1	[M+H]+	Adenosine 3'-monophosphate	Nucleotide and its derivates	1400000	1520000	1750000	1730000	1730000	1520000	1130000	1200000	1320000	1280000	1290000	1210000
Mul0403	Negative	346	78.8	1.17	347.063	[M-H]-	Adenosine 5'-monophosphate	Nucleotide and its derivates	239000	181000	187000	42800	93800	23400	484000	465000	457000	208000	201000	194000
Mul0124	Positive	400.2	136.1	1.97	399.2	[M+H]+	Adenosine O-ribose	Nucleotide and its derivates	49900	122000	114000	54200	56400	62400	71600	48600	52800	55200	67000	66900
Mul0438	Negative	328.1	134.2	1.84	329.1	[M-H]-	Cyclic AMP	Nucleotide and its derivates	250000	60600	55400	9260	8240	4430	21900	49800	40700	56900	45800	32100
Mul0051	Positive	244.086	111.4	0.8	243.086	[M+H]+	Cytidine	Nucleotide and its derivates	3430000	2950000	7040000	1260000	951000	1130000	553000	720000	697000	1670000	1700000	1690000
Mul0388	Negative	322	78.8	0.82	323.052	[M-H]-	Cytidine 5'-monophosphate (Cytidylic acid)	Nucleotide and its derivates	9790	9270	17700	14300	18200	10200	23600	19000	24100	16500	11500	16100
Mul0023	Positive	112	95	0.73	111.0433	[M+H]+	Cytosine	Nucleotide and its derivates	157000	183000	378000	71200	43600	60500	25700	36100	50100	80500	91000	101000
Mul0123	Positive	252	136.1	1.96	251.102	[M+H]+	Deoxyadenosine	Nucleotide and its derivates	202000	133000	292000	128000	149000	225000	236000	193000	261000	196000	205000	211000
Mul0075	Positive	228	112	1.17	227.091	[M+H]+	Deoxycytidine	Nucleotide and its derivates	19200	10600	35100	9	6600	9290	19400	9	14300	18600	12500	10800
Mul0112	Positive	268.097	151.7	1.73	267.097	[M+H]+	Deoxyguanosine	Nucleotide and its derivates	97900	52400	359000	39700	54200	59000	63800	55500	51300	72400	81200	79400
Mul0419	Negative	213	78.9	1.23	214.016	[M-H]-	Deoxyribose 5-phosphate	Nucleotide and its derivates	9	9	9	9	9	9	54300	56100	66100	31200	34200	30900
Mul0479	Negative	784.2	437.2	2.69	785.157	[M-H]-	Flavin adenine dinucleotide (FAD)	Nucleotide and its derivates	421000	313000	374000	92700	62300	81000	506000	626000	474000	108000	159000	126000
Mul0406	Negative	150	132.9	1.18	151.049	[M-H]-	Guanine	Nucleotide and its derivates	40900	32800	34500	534	9	9	11700	8480	13200	6330	6480	6680
Mul0432	Negative	282.092	150.1	1.44	283.092	[M-H]-	Guanosine	Nucleotide and its derivates	3780000	2740000	2090000	256000	427000	226000	920000	925000	826000	1250000	1180000	1250000
Mul0436	Negative	344.2	344	1.68	345.047	[M-H]-	Guanosine 3',5'-cyclic monophosphate	Nucleotide and its derivates	29800	16100	11000	10400	4840	4990	8630	9790	6360	7950	7980	9050
Mul0405	Negative	362.1	211.1	1.18	363.1	[M-H]-	Guanosine 5'-monophosphate	Nucleotide and its derivates	110000	82100	44300	48900	69700	38900	140000	129000	128000	91000	81300	92800
Mul0407	Negative	362	78.9	1.18	363.058	[M-H]-	Guanosine monophosphate	Nucleotide and its derivates	183000	130000	44900	26200	31100	33600	79300	82800	78500	72400	68100	68700
Mul0080	Positive	137	110	1.2	136.0385	[M+H]+	Hypoxanthine	Nucleotide and its derivates	78800	146000	71000	7870	18000	15200	19200	17100	25600	31300	38200	36300
Mul0430	Negative	267.08	135.1	1.41	268.081	[M-H]-	Hypoxanthine-9-β-D-arabinofuranoside	Nucleotide and its derivates	381000	221000	282000	242000	458000	220000	285000	131000	191000	264000	250000	241000
Mul0049	Positive	269.1	137	0.8	268.081	[M+H]+	Inosine	Nucleotide and its derivates	10700	7780	9150	85900	67300	70900	76900	69800	59300	50600	52800	61500
Mul0073	Positive	349.1	137.4	1.17	348.1	[M+H]+	Inosine 5'-monophosphate	Nucleotide and its derivates	39800	64100	47600	44800	38700	39100	76400	76000	61100	43000	49100	43300
Mul0061	Positive	366.2	203.5	0.82	365.2	[M+H]+	iP7G	Nucleotide and its derivates	95300	143000	103000	74200	104000	78400	42900	73300	77200	92700	83200	75400
Mul0070	Positive	298.1	166.5	1.17	297.1	[M+H]+	N2-methylguanosine	Nucleotide and its derivates	201000	105000	39500	20100	25700	18200	14900	20000	25200	52400	43100	42600
Mul0451	Negative	382	134.1	2.17	383.108	[M-H]-	N6-Succinyl Adenosine	Nucleotide and its derivates	264000	174000	203000	316000	258000	272000	632000	456000	368000	326000	325000	330000
Mul0072	Positive	664.2	524.1	1.17	663.2	[M+H]+	Nicotinic acid adenine dinucleotide	Nucleotide and its derivates	711000	537000	592000	827000	1090000	666000	1600000	1530000	1600000	1080000	1060000	933000
Mul0127	Positive	384.115	252.2	2.13	383.115	[M+H]+	Succinyladenosine	Nucleotide and its derivates	322000	654000	556000	377000	299000	465000	936000	483000	477000	425000	517000	542000
Mul0125	Positive	243.09	127.1	2.01	242.09	[M+H]+	Thymidine	Nucleotide and its derivates	31100	32300	60700	9	9	9	9	9	9	14600	24000	18100
Mul0079	Positive	113.027	95.9	1.19	112.027	[M+H]+	Uracil	Nucleotide and its derivates	154000	112000	117000	24000	13700	20900	39400	38500	26300	85600	62400	41600
Mul0431	Negative	243.1	110	1.42	244.07	[M-H]-	Uridine	Nucleotide and its derivates	714000	495000	715000	13600	50800	13700	335000	410000	354000	194000	209000	254000
Mul0381	Negative	403	159	0.78	404.161	[M-H]-	Uridine 5’-diphosphate	Nucleotide and its derivates	120000	123000	101000	101000	109000	100000	104000	114000	122000	117000	114000	124000
Mul0404	Negative	565.1	323.3	1.18	566.1	[M-H]-	Uridine 5'-diphospho-D-glucose	Nucleotide and its derivates	308000	165000	627000	9	4440000	9	3350000	2780000	2170000	2550000	2350000	2400000
Mul0420	Negative	323	78.8	1.23	324.036	[M-H]-	Uridine 5'-monophosphate	Nucleotide and its derivates	181000	92400	52000	43600	47600	34900	160000	149000	116000	102000	92200	97000
Mul0071	Positive	153	136.4	1.17	152	[M+H]+	Xanthine	Nucleotide and its derivates	114000	203000	82200	42500	26400	47000	11900	12000	15400	40700	40300	35800
Mul0113	Positive	285.1	153.1	1.74	284.076	[M+H]+	Xanthosine	Nucleotide and its derivates	2430000	1790000	1740000	376000	427000	327000	680000	675000	606000	1090000	998000	1040000
Mul0045	Positive	335.1	122.8	0.79	334.0566	[M+H]+	β-Nicotinamide mononucleotide	Nucleotide and its derivates	427000	439000	407000	738000	588000	676000	281000	200000	275000	400000	339000	315000
Mul0411	Negative	243	153.1	1.2	244.07	[M-H]-	β-Pseudouridine	Nucleotide and its derivates	95500	66400	121000	9	12300	9	93400	100000	78400	36500	27700	50500

Mul0145	Positive	449.1	287.3	2.45	449.1	Protonated	Cyanidin 3-O-glucoside (Kuromanin)	Anthocyanins	9	9	9	35400000	36500000	31700000	9	9	9	40700000	40600000	40300000
Mul0159	Positive	535.1	287.4	2.81	535.1	Protonated	Cyanidin 3-O-malonylhexoside	Anthocyanins	9	9	9	259000	227000	238000	9	9	9	88300	103000	85500
Mul0150	Positive	595	287.9	2.6	595	Protonated	Cyanidin 3-O-rutinoside (Keracyanin)	Anthocyanins	1860000	256000	51800	29000000	28000000	28400000	13500000	20700000	18800000	42200000	38400000	41300000
Mul0484	Negative	489.1	285.3	2.82	490.1	[M-H]-	Cyanidin O-acetylhexoside	Anthocyanins	243000	215000	292000	9	9	9	205000	194000	231000	162000	170000	148000
Mul0460	Negative	465.1	285.3	2.41	466.1	[M-H]-	Cyanidin O-syringic acid	Anthocyanins	9	9	9	91500000	1.36E+08	1.38E+08	4150000	6540000	7230000	1.17E+08	1.18E+08	79500000
Mul0165	Positive	303	149.3	2.9	303.24	Protonated	Delphinidin	Anthocyanins	96800	67700	97400	14400	9960	13700	33100	41800	36600	37100	33500	27700
Mul0135	Positive	465.1	303.1	2.3	465.1	Protonated	Delphinidin 3-O-glucoside (Mirtillin)	Anthocyanins	7950000	6120000	9300000	9	9	9	115000	141000	157000	290000	255000	442000
Mul0180	Positive	637.1	303.5	3.16	637.1	Protonated	Delphinidin O-malonyl-malonylhexoside	Anthocyanins	9	9	9	11800	10500	8350	15300	10600	9650	6800	6280	4660
Mul0160	Positive	493	331.7	2.81	493	Protonated	Malvidin 3-O-galactoside	Anthocyanins	9	9	9	60600	157000	52700	26300	37900	44100	339000	136000	196000
Mul0163	Positive	493.2	331.6	2.86	493.2	Protonated	Malvidin 3-O-glucoside (Oenin)	Anthocyanins	9	5030	24800	74900	172000	61900	32600	34100	38500	395000	161000	212000
Mul0148	Positive	433.1	271	2.58	433.1	Protonated	Pelargonidin 3-O-beta-D-glucoside（Callistephin chloride)	Anthocyanins	9	9	9	9	9	9	1910000	2920000	4630000	45100000	47400000	55300000
Mul0146	Positive	595	271.8	2.45	595	Protonated	Pelargonin	Anthocyanins	12600	7790	104000	142000	160000	135000	10600	16800	20900	86300	68600	71700
Mul0173	Positive	463.123	301.4	3	463.123	Protonated	Peonidin O-hexoside	Anthocyanins	9	9	9	8790000	7250000	6860000	225000	232000	269000	3280000	3370000	3160000
Mul0561	Negative	253.06	253.1	4.57	254.058	[M-H]-	7,4'-Dihydroxyflavone	Flavone	4280	8270	11400	6270	2580	2660	17100	12900	16500	8560	6360	9160
Mul0580	Negative	283.069	268	6.77	284.069	[M-H]-	Acacetin	Flavone	7820	5910	17900	9	9	9	9	9	9	2230	3990	2960
Mul0488	Negative	487.1	283.2	2.93	488.1	[M-H]-	Acacetin O-acetyl hexoside	Flavone	92200	49800	100000	9	9	9	9	9	9	26900	21800	19400
Mul0569	Negative	491.1	287.3	5.18	492.1	[M-H]-	Acetyl-eriodictyol O-hexoside	Flavone	9	9	9	9470	11500	12600	12000	17600	13300	6920	9280	11300
Mul0272	Positive	271.053	215.1	5.48	270.0528	[M+H]+	Apigenin	Flavone	112000	140000	382000	27000	35500	34300	122000	97700	337000	142000	151000	147000
Mul0220	Positive	433.106	313.1	3.73	432.1056	[M+H]+	Apigenin 5-O-glucoside	Flavone	28100	23000	102000	784000	909000	692000	46200	70600	159000	440000	484000	497000
Mul0542	Negative	431.1	269.2	4.07	432.1	[M-H]-	Apigenin 7-O-glucoside (Cosmosiin)	Flavone	25100	14800	41000	13800	9	9	61300	50000	66700	44000	33900	40600
Mul0248	Positive	519	271.7	4.36	518	[M+H]+	Apigenin O-malonylhexoside	Flavone	47100	25300	57200	35700	34600	44400	567000	766000	687000	268000	267000	254000
Mul0550	Negative	445.085	269	4.33	446.085	[M-H]-	Baicalein-7-O-glucuronide (Baicalin)	Flavone	9	9	9	18200	24500	15600	9	9	9	5060	5230	5110
Mul0271	Positive	273.07	153.1	5.45	272.069	[M+H]+	Butin	Flavone	426000	426000	136000	1190000	1140000	852000	240000	294000	307000	509000	503000	493000
Mul0291	Positive	255.058	69.7	6.73	254.0579	[M+H]+	Chrysin	Flavone	36200	36500	62400	38100	39400	40300	23500	21800	35000	37000	38300	33700
Mul0259	Positive	417.1	255.7	4.84	416.1	[M+H]+	Chrysin 5-O-glucoside (Toringin)	Flavone	9790	7990	38100	27400	18300	25400	12800	12700	18300	18000	16800	14200
Mul0256	Positive	417.2	255.8	4.67	416.2	[M+H]+	Chrysin O-hexoside	Flavone	9	9	9	14500	14500	11000	8100	7720	24700	9280	10100	10400
Mul0266	Positive	503	255.6	5.24	502	[M+H]+	Chrysin O-malonylhexoside	Flavone	45300	26100	54900	98700	88200	84100	110000	139000	296000	103000	98600	88500
Mul0274	Positive	301.063	286.1	5.56	300.0634	[M+H]+	Chrysoeriol	Flavone	31700	30300	89700	151000	233000	183000	64400	56600	114000	88100	91900	89500
Mul0231	Positive	463.123	313.9	3.96	462.123	[M+H]+	Chrysoeriol 5-O-hexoside	Flavone	9	15000	27800	168000	220000	136000	61500	87000	87600	89000	65900	75800
Mul0241	Positive	463.2	287.7	4.14	462.2	[M+H]+	Chrysoeriol 7-O-hexoside	Flavone	19300	10500	21000	171000	243000	161000	59900	77700	90500	75300	81800	78100
Mul0535	Negative	607.1	299.1	3.93	608.1	[M-H]-	Chrysoeriol 7-O-rutinoside	Flavone	9	9	9	219000	201000	212000	230000	254000	198000	337000	189000	282000
Mul0530	Negative	503.1	341.3	3.88	504.1	[M-H]-	Chrysoeriol O-acetylhexoside	Flavone	263000	384000	406000	22300	9	17400	2860000	2740000	2710000	1430000	859000	1280000
Mul0237	Positive	625.1	331.7	4.03	624.1	[M+H]+	Chrysoeriol O-hexosyl-O-hexoside	Flavone	30900	22900	44100	9	9	9	9	9	9	16500	19000	18100
Mul0198	Positive	509.3	347.9	3.47	508.3	[M+H]+	Limocitrin O-hexoside	Flavone	9	9	9	1030000	1010000	1050000	1010000	855000	985000	714000	665000	614000
Mul0262	Positive	287.1	287.1	4.91	286.1	[M+H]+	Luteolin	Flavone	1630000	1890000	4210000	219000	470000	252000	156000	121000	1060000	1110000	1010000	1030000
Mul0193	Positive	611.1	449.4	3.38	610.1	[M+H]+	Luteolin 3',7-di-O-glucoside	Flavone	597000	492000	1020000	93100	99600	113000	191000	192000	210000	266000	290000	287000
Mul0221	Positive	449	287.1	3.76	448	[M+H]+	Luteolin 7-O-glucoside	Flavone	1020000	1230000	1750000	5190000	9	9	9	9	9	3380000	2580000	2850000
Mul0517	Negative	447.3	285.1	3.7	448.101	[M-H]-	Luteolin 7-O-glucoside (Cynaroside)	Flavone	384000	413000	525000	1250000	985000	646000	317000	333000	362000	631000	588000	578000
Mul0467	Negative	771.1	609.5	2.47	772.1	[M-H]-	Luteolin O-hexosyl-O-hexosyl-O-hexoside	Flavone	3580000	2810000	4040000	6160	9	7310	22400	41800	57200	113000	149000	159000
Mul0294	Positive	403.132	373.1	6.84	402.132	[M+H]+	Nobiletin	Flavone	65800	88500	94900	80900	81400	118000	69700	78200	68600	66300	75900	83800
Mul0267	Positive	477.2	328	5.28	476.2	[M+H]+	O-methylChrysoeriol 7-O-hexoside	Flavone	9	9	9	3400	9	4600	15600	5760	27800	7610	7040	7020
Mul0292	Positive	287.084	287.1	6.73	286.084	[M+H]+	sakuranetin	Flavone	9	9	9	9	9	4780	21600	9	9	4870	4440	4140
Mul0203	Positive	479.118	302.8	3.54	478.118	[M+H]+	Selgin 5-O-hexoside	Flavone	46200	30700	22700	17600	11500	9670	13700	9	9	24100	13900	18600
Mul0243	Positive	565	317.8	4.17	564	[M+H]+	Selgin O-malonylhexoside	Flavone	35400	41500	32700	11900	10400	9490	120000	175000	165000	60900	62700	57300
Mul0223	Positive	509.2	347.6	3.82	508.2	[M+H]+	Syringetin 5-O-hexoside	Flavone	9	9	9	68900	95500	94100	107000	104000	87900	95200	101000	98700
Mul0235	Positive	509.3	283.6	4.02	508.3	[M+H]+	Syringetin 7-O-hexoside	Flavone	9	9	9	20300	19800	24700	9	9	9	6470	5590	8580
Mul0303	Positive	373.121	373.1	7.32	372.121	[M+H]+	Tangeretin	Flavone	60600	96900	81600	86300	75700	122000	69300	71200	67900	71300	78500	87000
Mul0553	Negative	301	149.1	4.38	302.043	[M-H]-	Tricetin	Flavone	89100	83800	78700	19500	30300	20200	15800	18400	22900	37200	38000	43000
Mul0533	Negative	491.1	329.3	3.9	492.1	[M-H]-	Tricin 5-O-hexoside	Flavone	648000	483000	1060000	768000	664000	762000	76600	67700	53400	444000	440000	427000
Mul0536	Negative	637.1	269.3	3.93	638.1	[M-H]-	Tricin 5-O-rutinoside	Flavone	9	9	9	23800	16200	24000	6630	5530	5160	6340	5740	3180
Mul0547	Negative	491.1	431.2	4.14	492.1	[M-H]-	Tricin 7-O-hexoside	Flavone	26200	25500	26400	9	9	9	9	9	9	14300	11100	9120
Mul0189	Positive	655.2	331.7	3.35	654.2	[M+H]+	Tricin 7-O-hexosyl-O-hexoside	Flavone	4560	9	9	51600	52100	44500	31500	33700	39900	42000	29300	29800
Mul0515	Negative	521.1	329.3	3.68	522.1	[M-H]-	Tricin O-saccharic acid	Flavone	2720000	2400000	1720000	2150000	1800000	2570000	613000	617000	496000	1960000	1860000	1850000
Mul0514	Negative	535	329.4	3.68	536	[M-H]-	Tricin O-sinapic acid	Flavone	67800	29900	75700	630000	512000	524000	80900	97600	69100	211000	214000	203000

Mul0556	Negative	287.06	125.1	4.43	288.063	[M-H]-	Aromadedrin (Dihydrokaempferol)	Flavonol	1940000	2220000	480000	608000	944000	935000	746000	486000	751000	1100000	962000	1010000
Mul0191	Positive	321.05	153.1	3.36	320.053	[M+H]+	Dihydromyricetin	Flavonol	9	9	9	83200	42100	75600	96400	72200	39400	35600	34600	30900
Mul0538	Negative	303.058	125.1	3.94	304.058	[M-H]-	Dihydroquercetin (Taxifolin)	Flavonol	9570000	9690000	1680000	3970000	8010000	4790000	5240000	3630000	6870000	6130000	5800000	5750000
Mul0577	Negative	329.1	314.3	5.76	330.1	[M-H]-	Di-O-methylquercetin	Flavonol	1820000	397000	1660000	273000	289000	270000	2150000	1250000	2550000	1230000	1010000	1090000
Mul0211	Positive	289	215.1	3.66	288.063	[M+H]+	Fustin	Flavonol	7610	7560	10200	18100	28900	28500	29200	26400	27300	15800	13000	13500
Mul0565	Negative	675.2	513.2	4.83	676.237	[M-H]-	Icariin (kaempferol 3,7-O-diglucoside 8-prenyl derivative)	Flavonol	29500	40100	27200	9850	9	9	9	9	9	18500	19400	16100
Mul0210	Positive	625.169	317.1	3.64	624.169	[M+H]+	Isorhamnetin 3-O-neohesperidoside	Flavonol	9	9	9	122000	140000	120000	82400	86300	76700	55100	36200	68200
Mul0227	Positive	479.2	317.8	3.86	478.2	[M+H]+	Isorhamnetin 5-O-hexoside	Flavonol	103000	110000	84600	65900	72700	64000	201000	119000	365000	134000	147000	126000
Mul0548	Negative	519.1	315.3	4.22	520.1	[M-H]-	Isorhamnetin O-acetyl-hexoside	Flavonol	26200	23000	21900	2330	9	2160	81600	106000	106000	39000	44400	38400
Mul0244	Positive	479.2	318	4.25	478.2	[M+H]+	Isorhamnetin O-hexoside	Flavonol	98600	105000	94200	59400	57700	59400	196000	146000	380000	141000	117000	117000
Mul0273	Positive	287.048	287	5.52	286.048	[M+H]+	Kaempferol	Flavonol	32900	41400	15200	17400	36500	34900	4660	7840	39300	25900	22400	24800
Mul0213	Positive	579.2	433.1	3.67	578.164	[M+H]+	Kaempferol 3,7-dirhamnoside (Kaempferitrin)	Flavonol	9	9	9	40600	33500	48400	9	17500	21700	13000	13800	13500
Mul0528	Negative	447	284	3.86	448.101	[M-H]-	Kaempferol 3-O-galactoside (Trifolin)	Flavonol	3090000	2350000	3810000	2600000	2120000	2930000	1230000	2180000	1690000	2520000	2420000	2590000
Mul0238	Positive	449.1	287.6	4.03	448.1	[M+H]+	Kaempferol 3-O-glucoside (Astragalin)	Flavonol	343000	196000	356000	242000	232000	195000	85800	138000	108000	208000	193000	187000
Mul0217	Positive	595.159	287	3.7	594.159	[M+H]+	Kaempferol 3-O-robinobioside (Biorobin)	Flavonol	9	9	9	17500000	17500000	15200000	2940000	4480000	2770000	6980000	7000000	6890000
Mul0524	Negative	593.159	285.1	3.77	594.1585	[M-H]-	Kaempferol 3-O-rutinoside (Nicotiflorin)	Flavonol	9	9	9	26800000	23500000	26100000	3010000	4190000	3560000	6980000	6990000	7390000
Mul0503	Negative	739.22	593.3	3.28	740.216	[M-H]-	Kaempferol-3-O-robinoside-7-O-rhamnoside (Robinin)	Flavonol	9	9	9	37200	23500	29200	5820	8600	5220	7790	9270	7970
Mul0264	Positive	333	305.2	4.96	332.053	[M+H]+	Laricitrin	Flavonol	9	6090	9	7490	11000	9710	8030	6100	11000	6450	5460	6220
Mul0225	Positive	479	317.3	3.84	478	[M+H]+	methylQuercetin O-hexoside	Flavonol	75600	52600	69100	44200	52900	41200	164000	134000	290000	95400	98800	94100
Mul0567	Negative	301	151.1	4.97	302.04265	[M-H]-	Morin	Flavonol	52300	136000	18800	560000	1470000	722000	47600	50600	202000	519000	273000	378000
Mul0554	Negative	317	179	4.39	318.038	[M-H]-	Myricetin	Flavonol	11900	25500	9530	13500	26700	14600	8850	12300	35500	20400	16800	17600
Mul0506	Negative	479.09	317.3	3.43	480.09	[M-H]-	Myricetin 3-O-galactoside	Flavonol	6220	9	4930	24300	15700	26800	9	3350	3630	8520	9740	7190
Mul0263	Positive	303.043	303	4.92	302.043	[M+H]+	Quercetin	Flavonol	159000	194000	31600	238000	204000	279000	103000	50900	438000	167000	156000	156000
Mul0229	Positive	435.085	303	3.91	434.0849	[M+H]+	Quercetin 3-alpha-L-arabinofuranoside (Avicularin)	Flavonol	7700	8680	9790	300000	322000	255000	26400	42800	55000	95800	132000	91800
Mul0516	Negative	463	301.1	3.68	464.096	[M-H]-	Quercetin 3-O-glucoside (Isotrifoliin)	Flavonol	12500000	10300000	12900000	44800000	43200000	45000000	18400000	25700000	24200000	25900000	26600000	24900000
Mul0201	Positive	611.153	303	3.51	610.153	[M+H]+	Quercetin 3-O-rutinoside (Rutin)	Flavonol	9	9	9	36900000	38300000	31300000	22900000	27300000	22600000	26900000	24900000	26100000
Mul0239	Positive	465	302.9	4.04	464.096	[M+H]+	Quercetin 4'-O-glucoside (Spiraeoside)	Flavonol	17700000	12600000	16600000	29500000	31200000	31600000	21900000	25700000	24100000	26200000	25000000	24900000
Mul0162	Positive	713.1	713.1	2.83	712.1	[M+H]+	Quercetin 5-O-malonylhexosyl-hexoside	Flavonol	403000	420000	453000	9	9	9	1770000	1610000	2090000	331000	385000	407000
Mul0195	Positive	713.1	303.6	3.41	712.1	[M+H]+	Quercetin 7-O-malonylhexosyl-hexoside	Flavonol	169000	134000	162000	9	9	9	229000	199000	151000	105000	163000	112000
Mul0219	Positive	611.2	303.7	3.73	610.2	[M+H]+	Quercetin 7-O-rutinoside	Flavonol	9	9	9	37300000	33000000	34600000	21800000	23800000	22300000	24700000	24300000	23200000
Mul0529	Negative	505.1	301.2	3.87	506.1	[M-H]-	Quercetin O-acetylhexoside	Flavonol	5310000	4110000	6100000	157000	142000	129000	27000000	30400000	20700000	16900000	14600000	15800000
Mul0578	Negative	315	165.1	6.19	316.058	[M-H]-	Rhamnetin (7-O-methxyl quercetin)	Flavonol	5100	4890	7350	2190	9	9	3230	3000	3140	4140	3340	3480
Mul0184	Positive	509.1	347.7	3.27	508.1	[M+H]+	Syringetin 3-O-hexoside	Flavonol	9	9	9	181000	174000	222000	243000	195000	250000	127000	128000	98600
Mul0555	Negative	657.1	495.3	4.43	658.1	[M-H]-	Tricin 4'-O-(syringyl alcohol) ether 5-O-hexoside	Flavonolignan	28000	38600	22300	21300	18000	25100	24900	23700	14500	17500	20200	19200
Mul0187	Positive	627.1	447.3	3.33	626.1	[M+H]+	6-C-hexosyl-hesperetin O-hexoside	Flavone C-glycosides	1170000	1090000	1430000	208000	160000	259000	99200	115000	105000	524000	531000	451000
Mul0164	Positive	611.1	329	2.9	610.1	[M+H]+	6-C-hexosyl-luteolin O-hexoside	Flavone C-glycosides	146000	144000	156000	9	9	9	27500	33800	17200	35900	24600	24200
Mul0181	Positive	757.2	757	3.19	756.2	[M+H]+	8-C-hexosyl-apigenin O-hexosyl-O-hexoside	Flavone C-glycosides	9	9	9	35500	25000	42800	15500	12400	11700	14600	12000	17800
Mul0152	Positive	627.1	430	2.65	626.1	[M+H]+	8-C-hexosyl-hesperetin O-hexoside	Flavone C-glycosides	15800000	14200000	19400000	2420000	1580000	2390000	7890000	8160000	10000000	5660000	5450000	5340000
Mul0194	Positive	581.1	449.2	3.38	580.1	[M+H]+	8-C-hexosyl-luteolin O-pentoside	Flavone C-glycosides	20100	20400	9	45800	24700	28100	21300	18300	18800	16900	25700	23700
Mul0228	Positive	433.113	271.7	3.88	432.113	[M+H]+	Apigenin C-glucoside	Flavone C-glycosides	26900	39700	66600	3080	9	2830	9	9	9	14400	15800	11000
Mul0207	Positive	757.3	757.2	3.61	756.3	[M+H]+	C-hexosyl-apigenin O-caffeoylhexoside	Flavone C-glycosides	9	9	9	5800	11900	16000	5490	7790	4690	6360	8790	12200
Mul0206	Positive	757.2	757	3.6	756.2	[M+H]+	C-hexosyl-luteolin O-p-coumaroylhexoside	Flavone C-glycosides	9	9	9	17500	28500	26700	12000	7290	6060	5090	10800	8340
Mul0153	Positive	775.1	774.9	2.68	774.1	[M+H]+	Eriodictiol 6-C-hexoside 8-C-hexoside-O-hexoside	Flavone C-glycosides	9	9	9	28600	25100	27100	19500	30000	21600	11700	18500	15000
Mul0169	Positive	613.1	300.3	2.97	612.1	[M+H]+	Eriodictiol C-hexosyl-O-hexoside	Flavone C-glycosides	863000	773000	1120000	99300	84000	121000	81000	120000	108000	278000	307000	305000

Mul0501	Negative	449.1	329.3	3.21	450.1	[M-H]-	Eriodictyol C-hexoside	Flavone C-glycosides	2700000	1630000	1550000	5260000	7030000	4800000	1510000	1470000	1680000	3080000	3010000	2560000
Mul0208	Positive	433.106	313.1	3.61	432.106	[M+H]+	Isovitexin	Flavone C-glycosides	37600	35800	73200	3140	9	9	9	2150	9	14800	17400	16200
Mul0212	Positive	611.2	299.8	3.67	610.2	[M+H]+	Luteolin 8-C-hexosyl-O-hexoside	Flavone C-glycosides	9	9	9	4870000	4560000	4030000	1230000	1550000	865000	1360000	1570000	1570000
Mul0218	Positive	435.1	339.7	3.73	434.1	[M+H]+	Naringenin C-hexoside	Flavone C-glycosides	21900	13700	19300	9	9	6680	4970	7550	9	4860	6570	11000
Mul0240	Positive	477.1	298	4.06	476.1	[M+H]+	O-methylChrysoeriol 8-C-hexoside	Flavone C-glycosides	55400	44000	44800	9	9	9	9	9	9	16400	14000	12800
Mul0222	Positive	419.1	383.9	3.79	418.1	[M+H]+	O-methylnaringenin C-pentoside	Flavone C-glycosides	9	12100	7410	26400	39500	38300	9	9	9	11900	8000	15100
Mul0186	Positive	275	139.1	3.29	274.084	[M+H]+	Afzelechin (3,5,7,4'-Tetrahydroxyflavan)	Flavanone	16100	14300	2380	29200	47600	56400	35000	18000	54700	24900	24000	34900
Mul0269	Positive	273.1	137.1	5.3	272.069	[M+H]+	Butein	Flavanone	7220000	12100000	8890000	2190000	3000000	2370000	668000	826000	1960000	4610000	4580000	4930000
Mul0260	Positive	289.063	153.1	4.85	288.063	[M+H]+	Eriodictyol	Flavanone	1420000	1730000	453000	12500000	7950000	6440000	710000	1320000	1380000	3460000	3310000	3450000
Mul0250	Positive	537	289.7	4.39	536	[M+H]+	Eriodictyol O-malonylhexoside	Flavanone	14800	9910	12700	481000	661000	335000	25200	76100	65000	117000	120000	104000
Mul0276	Positive	303.079	303.1	5.63	302.079	[M+H]+	Hesperetin	Flavanone	9	9	9	27900	18000	17900	6640	8560	5980	6470	9960	7390
Mul0531	Negative	463.132	301.1	3.88	464.132	[M-H]-	Hesperetin 5-O-glucoside	Flavanone	12600000	11100000	13000000	45000000	42700000	43500000	19600000	27000000	25100000	27100000	26700000	25000000
Mul0546	Negative	609.2	301.3	4.09	610.19	[M-H]-	Hesperetin 7-O-neohesperidoside (Neohesperidin)	Flavanone	9	9	9	7120000	6010000	5540000	747000	596000	628000	1930000	1550000	1430000
Mul0233	Positive	611.19	303	3.99	610.19	[M+H]+	Hesperetin 7-rutinoside (Hesperidin)	Flavanone	9	9	9	17600000	16900000	19800000	3140000	3510000	4390000	7080000	7500000	6800000
Mul0457	Negative	625.2	463.3	2.26	626.2	[M-H]-	Hesperetin O-hexosyl-O-hexoside	Flavanone	9	9	9	5960	9	9	13500	14600	11600	7750	6680	4930
Mul0534	Negative	549.2	387.3	3.93	550.2	[M-H]-	Hesperetin O-malonylhexoside	Flavanone	217000	209000	226000	53200	47600	63400	60800	49100	34700	128000	114000	131000
Mul0575	Negative	301.1	151	5.55	302.27876	[M-H]-	Homoeriodictyol	Flavanone	3270	5360	3780	11700	8070	8960	9	2090	9100	5130	4400	4630
Mul0282	Positive	257	137.1	5.91	256.074	[M+H]+	Isoliquiritigenin	Flavanone	204000	357000	172000	904000	792000	651000	147000	142000	165000	342000	343000	367000
Mul0261	Positive	257	137.1	4.87	256.074	[M+H]+	Liquiritigenin	Flavanone	9	6660	5780	47700	20300	19900	9	5960	9	9750	10500	11300
Mul0573	Negative	271.069	151	5.41	272.0685	[M-H]-	Naringenin	Flavanone	283000	301000	83100	787000	745000	544000	179000	215000	195000	344000	320000	333000
Mul0541	Negative	433.121	122.9	4.05	434.1213	[M-H]-	Naringenin 7-O-glucoside (Prunin)	Flavanone	520000	365000	631000	2230000	2220000	1730000	449000	890000	512000	928000	1060000	975000
Mul0268	Positive	273.07	153.1	5.3	272.069	[M+H]+	Naringenin chalcone	Flavanone	330000	340000	112000	1100000	935000	672000	188000	212000	241000	424000	406000	415000
Mul0253	Positive	521	317.6	4.49	520	[M+H]+	Naringenin O-malonylhexoside	Flavanone	8610	7670	14100	36800	49500	40800	74300	140000	113000	49700	40300	44400
Mul0270	Positive	275.084	107	5.34	274.0841	[M+H]+	Phloretin	Flavanone	11500	6990	9	134000	129000	113000	9	4480	5960	37900	38800	35300
Mul0295	Positive	257.1	153	6.85	256.074	[M+H]+	Pinocembrin (Dihydrochrysin)	Flavanone	11600	5990	9	13600	23400	9420	10200	9540	62300	14800	16400	13300
Mul0324	Positive	355.15	178.9	8.24	354.147	[M+H]+	Xanthohumol	Flavanone	9	9	9	9	9	9	6690	4460	4120	2470	2300	3210
Mul0456	Negative	353.1	191.1	2.21	354.095	[M-H]-	1-O-Caffeoyl quinic acid	Quinate and its derivatives	36200000	42500000	37000000	29700000	31900000	34200000	39900000	37200000	36000000	30300000	38100000	38400000
Mul0197	Positive	369.1	207.5	3.47	368.1	[M+H]+	1-O-Feruloyl quinic acid	Quinate and its derivatives	1000000	1570000	1460000	85100	87000	115000	173000	115000	94600	517000	619000	719000
Mul0167	Positive	369.1	177.5	2.94	368.1	[M+H]+	3-O-Feruloyl quinic acid	Quinate and its derivatives	33700	42500	36500	9	9	9	20300	8890	14200	8320	9540	11200
Mul0508	Negative	337.1	190.9	3.44	338.1	[M-H]-	3-O-p-Coumaroyl quinic acid	Quinate and its derivatives	8510000	7600000	7750000	11300000	10400000	11900000	14300000	11900000	10200000	11100000	10800000	9180000
Mul0463	Negative	499.2	173.2	2.43	500.2	[M-H]-	3-O-p-coumaroyl quinic acid O-hexoside	Quinate and its derivatives	9	9	4260	4990	9	9	29800	24200	15500	10400	4370	10600
Mul0494	Negative	481.1	319.3	3.09	482.1	[M-H]-	3-O-p-coumaroyl shikimic acid O-hexoside	Quinate and its derivatives	9	9	9	44500	74900	36800	9	9	9	11900	15300	11600
Mul0489	Negative	353.1	191.2	2.95	354.1	[M-H]-	4-O-Caffeoyl quinic acid (criptochlorogenic acid)	Quinate and its derivatives	69200	58900	68000	78100	66600	80100	64400	59000	44600	70600	62800	55800
Mul0453	Negative	499.1	163.2	2.18	500.1	[M-H]-	5-O-p-coumaroyl quinic acid O-hexoside	Quinate and its derivatives	15200	7760	11500	22900	17800	22600	31000	22200	18100	15300	14700	21400
Mul0215	Positive	321.1	147.5	3.68	320.1	[M+H]+	5-O-p-Coumaroyl shikimic acid	Quinate and its derivatives	7020	4220	9	9	9	8530	1800000	1980000	974000	304000	288000	259000
Mul0475	Negative	481.1	445.4	2.6	482.1	[M-H]-	5-O-p-coumaroyl shikimic acid O-hexoside	Quinate and its derivatives	9270	7410	11600	143000	162000	173000	15000	17000	15400	50100	46500	43700
Mul0499	Negative	337	275.8	3.18	338	[M-H]-	5-O-p-Coumaroylquinic acid	Quinate and its derivatives	83000	82600	79400	61700	69400	78800	60700	43600	29900	65700	64200	61500
Mul0478	Negative	353.095	191.1	2.69	354.0951	[M-H]-	Chlorogenic acid (3-O-Caffeoylquinic acid)	Quinate and its derivatives	12100000	15200000	14600000	8760000	12200000	7060000	14800000	14500000	20500000	11500000	11400000	11200000
Mul0202	Positive	369.111	163	3.52	368.111	[M+H]+	Chlorogenic acid methyl ester	Quinate and its derivatives	9	9670	4240	29300	8500	28900	11000	6660	4830	10600	9870	10100
Mul0445	Negative	355.1	181.2	1.97	356.1	[M-H]-	Homovanilloyl quinic acid	Quinate and its derivatives	5070000	3320000	4800000	9	9	9	9	9	9	1590000	1440000	1540000
Mul0470	Negative	353	191.1	2.53	354.095	[M-H]-	Neochlorogenic acid (5-O-Caffeoylquinic acid)	Quinate and its derivatives	62500000	56000000	42800000	53800000	55300000	54600000	58400000	57200000	54500000	54700000	51200000	51200000
Mul0507	Negative	709.1	401.3	3.44	710.1	[M-H]-	O-p-Coumaroyl quinic acid O-rutinoside derivative	Quinate and its derivatives	3700	3630	2550	3800	9	9	2510	3250	9	4120	3970	5200
Mul0439	Negative	513.1	191.2	1.86	514.1	[M-H]-	p-Coumaroyl quinic acid O-glucuronic acid	Quinate and its derivatives	87800	34700	78900	9	9	9	22800	12900	5300	21000	29400	22900
Mul0442	Negative	371.1	179.2	1.92	372.1	[M-H]-	Quinacyl syringic acid	Quinate and its derivatives	42900	38400	41800	174000	149000	188000	26800	61800	48700	100000	94500	99800
Mul0413	Negative	191	85	1.22	192.063	[M-H]-	Quinic acid	Quinate and its derivatives	37500000	29200000	25700000	1.04E+08	57700000	82500000	11800000	10600000	11000000	37300000	42400000	43400000
Mul0446	Negative	369	191.1	1.98	370	[M-H]-	Quinic acid O-glucuronic acid	Quinate and its derivatives	54300	94900	68400	189000	136000	137000	81100	106000	77500	178000	80700	152000
Mul0137	Positive	474.2	327.8	2.34	473.2	[M+H]+	10-Formyl-THF	Others	940000	700000	888000	121000	108000	144000	677000	572000	472000	304000	295000	312000
Mul0083	Positive	96.04	80.2	1.21	95.04	[M+H]+	3-Hydroxypyridine	Others	6790	12000	5440	4420	6290	5570	6040	12100	8180	4840	4280	2600
Mul0012	Positive	169.09	134.1	0.66	168.09	[M+H]+	4-(Aminomethyl)-5-(hydroxymethyl)-2-methylpyridin-3-ol	Others	18700	36800	15100	6330	9900	8300	9	9	9	12200	9230	13400
Mul0147	Positive	144.1	142.9	2.55	143.1	[M+H]+	4-Methyl-5-thiazoleethanol	Others	9	5340	5540	7330	4080	6390	4390	4440	9	5940	5170	9150

Mul0009	Positive	140	98.9	0.63	139.027	[M+H]+	4-Nitrophenol	Others	21000000	37200000	21500000	25500000	23200000	29800000	22700000	19200000	19100000	22000000	24200000	27100000
Mul0101	Positive	137	110	1.37	136.039	[M+H]+	Allopurinol	Others	67100	129000	60100	9300	17100	14200	18000	17100	15200	19700	30100	32600
Mul0116	Positive	136.1	119.3	1.83	135.1	[M+H]+	Aminopurine	Others	224000	325000	346000	54100	95700	50500	64100	57100	78200	156000	171000	165000
Mul0166	Positive	491.1	329.7	2.92	490.1	[M+H]+	Anisic acid O-feruloyl hexoside	Others	8370	9300	12700	35500	29800	33900	9	4870	10100	23800	22100	20400
Mul0563	Negative	533.3	371.2	4.71	534.21	[M-H]-	Arctiin	Others	17900	18900	39300	6090	13700	9340	16200	18500	12000	17300	15200	16300
Mul0293	Positive	390.4	284.9	6.74	389.4	[M+H]+	Azoxystrobin acid	Others	131000	326000	201000	269000	259000	346000	155000	158000	182000	192000	240000	225000
Mul0129	Positive	122	105.3	2.19	121	[M+H]+	Benzamide	Others	4500	6270	5690	9360	9820	10300	21900	18400	24000	11900	13200	13200
Mul0308	Positive	387.1	331.8	7.48	386.1	[M+H]+	Cholesterol	Others	106000	138000	129000	152000	139000	196000	129000	124000	132000	110000	127000	151000
Mul0285	Positive	343.1	240.9	6.39	342.1	[M+H]+	Cocamidopropyl betaine	Others	17200	21600	20200	20400	14500	17200	16600	15100	18500	18100	15300	19800
Mul0298	Positive	302.4	284.9	7.15	301.4	[M+H]+	D-erythro-Dihydrosphingosine	Others	8460	8320	11900	137000	34700	107000	9290	9170	8130	18000	22600	18600
Mul0016	Positive	106	88.1	0.68	105.079	[M+H]+	Diethanolamine	Others	44200	44800	54400	18300	40800	20100	26400	29200	40700	33000	37800	26700
Mul0425	Negative	169	122.8	1.25	169.998	[M-H]-	Dl-Glyceraldehyde3-phosphate	Others	24600	27500	24200	9	86900	9	41900	51400	48800	52900	56300	56900
Mul0560	Negative	405.13	242.2	4.48	406.13	[M-H]-	E-3,4,5'-Trihydroxy-3'-glucopyranosylstilbene	Others	97100	95800	17300	9	9	9	9	9	9	36700	32100	39200
Mul0003	Positive	142.1	82.9	0.57	141.1	[M+H]+	Histidinol	Others	13100000	24400000	23100000	12100000	14300000	14700000	15400000	12800000	14300000	11700000	14400000	15900000
Mul0099	Positive	138.091	77.2	1.28	137.091	[M+H]+	Hydroxyphenethylamine	Others	38900	54300	32600	33800	26100	62900	12200	10200	10800	12600	16400	12900
Mul0362	Negative	179.157	87	0.74	180.0634	[M-H]-	Inositol	Others	1430000	1090000	1700000	253000	240000	254000	230000	234000	204000	510000	490000	456000
Mul0182	Positive	595.2	367.2	3.19	594.159	[M+H]+	Isovitexin 7-O-glucoside (Saponarin)	Others	9	9	9	19700	11800	35700	30100	39400	26600	21500	14200	11400
Mul0030	Positive	163	103	0.76	162.113	[M+H]+	L-Carnitine	Others	25300	58700	31900	23000	19000	32500	44000	32300	29600	17300	35900	37200
Mul0327	Positive	400.33	341.4	8.31	399.335	[M+H]+	L-Palmitoylcarnitine	Others	9	9	9	22600	10800	23600	11900	8510	4930	6170	8830	7710
Mul0415	Negative	742.1	620.2	1.22	743.075	[M-H]-	NADP	Others	74200	65000	80500	129000	135000	154000	108000	106000	97200	158000	110000	130000
Mul0281	Positive	275.1	258.1	5.9	274.1	[M+H]+	N-Lauryldiethanolamine	Others	55100	46700	53100	96800	86100	101000	82200	92600	74200	65100	73300	72900
Mul0370	Negative	140	78.5	0.76	141.019	[M-H]-	O-Phosphorylethanolamine	Others	21900	12900	20300	19200	29200	14000	33600	35800	37100	34300	24100	21700
Mul0410	Negative	287.06	153.1	1.19	288.059	[M-H]-	Orotidine	Others	66700	50500	39200	9500	13300	10800	9790	8780	5830	16100	16400	16800
Mul0209	Positive	503.2	311.8	3.64	502.2	[M+H]+	Phellodensin F	Others	1360000	1080000	1960000	301000	169000	294000	254000	203000	123000	590000	674000	434000
Mul0141	Positive	122.1	105	2.39	121.089	[M+H]+	Phenethylamine	Others	28400	68400	17900	28000	15000	39400	16900	10800	15300	21900	20800	20100
Mul0348	Positive	149	121.3	9.57	148	[M+H]+	Phthalic anhydride	Others	42500	36100	40000	83700	50400	58900	74800	71900	60900	55500	60600	60200
Mul0496	Negative	385.1	223.2	3.1	386.1	[M-H]-	1-O-beta-D-Glucopyranosyl sinapate	Hydroxycinnamoyl derivatives	116000	94900	260000	73500	60600	78700	584000	486000	447000	236000	210000	234000
Mul0545	Negative	151.047	136.1	4.08	152.0473	[M-H]-	2-Methoxybenzoic acid	Hydroxycinnamoyl derivatives	49200	35500	45100	12800	17300	21000	11300	4840	11000	27200	26000	29000
Mul0204	Positive	167.063	149.1	3.57	166.063	[M+H]+	3-(4-Hydroxyphenyl)propionic acid	Hydroxycinnamoyl derivatives	43400	50700	39100	19500	24600	19900	19300	9	35200	30900	33000	18200
Mul0568	Negative	237.084	103.1	5	238.0841	[M-H]-	3,4,5-Trimethoxycinnamic acid	Hydroxycinnamoyl derivatives	19000	9	9	5330	7620	6490	7170	3540	3870	2860	3510	3210
Mul0574	Negative	207.1	192.1	5.53	208.1	[M-H]-	3,4-Dimethoxycinnamic acid	Hydroxycinnamoyl derivatives	9120	7830	10600	9	9	9	1650	9	9	6040	4460	3760
Mul0540	Negative	193.058	134.1	4.01	194.0579	[M-H]-	3-Hydroxy-4-methoxycinnamic acid	Hydroxycinnamoyl derivatives	324000	351000	220000	111000	184000	175000	193000	210000	172000	228000	224000	229000
Mul0572	Negative	177	145.2	5.36	178	[M-H]-	4-Methoxycinnamic acid	Hydroxycinnamoyl derivatives	264000	261000	132000	2580	4350	3340	20600	12500	13200	95500	92100	91400
Mul0178	Positive	207.1	147.4	3.1	206.1	[M+H]+	6-Hydroxymethylherniarin	Hydroxycinnamoyl derivatives	195000	180000	264000	137000	97500	111000	221000	189000	174000	150000	182000	153000
Mul0310	Positive	317.2	281.3	7.53	316.204	[M+H]+	Cafestol	Hydroxycinnamoyl derivatives	17800	16200	8780	34600	22100	25800	29700	9010	18500	14700	12600	23500
Mul0179	Positive	181.042	135.1	3.14	180.042	[M+H]+	Caffeic acid	Hydroxycinnamoyl derivatives	23800	25300	20700	59600	57600	65900	78000	76700	54300	45000	40800	38600
Mul0492	Negative	341	179.2	3.03	342	[M-H]-	Caffeic acid O-glucoside	Hydroxycinnamoyl derivatives	487000	479000	392000	280000	293000	340000	677000	651000	532000	478000	404000	430000
Mul0279	Positive	165.1	95.5	5.88	164.1	[M+H]+	Caffeic aldehyde	Hydroxycinnamoyl derivatives	9040	4990	7390	7430	9	4150	10500	9680	6010	6620	7770	7140
Mul0452	Negative	311.1	149.2	2.18	312.1	[M-H]-	Caftaric acid	Hydroxycinnamoyl derivatives	9240	6520	10600	51300	43700	51700	9640	9	8800	20200	17900	15400
Mul0571	Negative	147.052	61.8	5.22	148.0524	[M-H]-	Cinnamic acid	Hydroxycinnamoyl derivatives	1240000	1200000	196000	43700	41300	30000	20500	18600	129000	410000	378000	366000
Mul0477	Negative	341	179.1	2.62	342.132	[M-H]-	Coniferin	Hydroxycinnamoyl derivatives	5730000	5180000	5850000	127000	115000	127000	2370000	1990000	1590000	929000	772000	795000
Mul0519	Negative	179.079	146.1	3.71	180.079	[M-H]-	Coniferyl alcohol	Hydroxycinnamoyl derivatives	7970	5440	11000	12800	10500	9730	8230	10000	9	8050	9140	7740
Mul0559	Negative	177.063	177.1	4.46	178.063	[M-H]-	Coniferylaldehyde	Hydroxycinnamoyl derivatives	381000	244000	714000	556000	416000	553000	525000	440000	304000	495000	451000	468000
Mul0230	Positive	195.058	149.1	3.92	194.0579	[M+H]+	Ferulic acid	Hydroxycinnamoyl derivatives	72600	44600	34000	18900	30800	33600	28000	35500	35100	40300	33200	35000
Mul0188	Positive	375.2	137.6	3.34	374.2	[M+H]+	Feruloyl syringic acid	Hydroxycinnamoyl derivatives	14100	26800	28700	21300	18500	19700	9190	9	4780	8210	12400	14700

Mul0185	Positive	183.058	137.1	3.28	182.0579	[M+H]+	Homovanillic acid	Hydroxycinnamoyl derivatives	1120000	793000	1170000	127000	95700	107000	107000	80300	47800	486000	461000	465000
Mul0265	Positive	151.068	105	5.17	150.068	[M+H]+	Hydrocinnamic acid	Hydroxycinnamoyl derivatives	19400	26200	9	9	9	9	9	9	9	9840	9520	10100
Mul0176	Positive	195.1	177.5	3.08	194.1	[M+H]+	Hydroxy-methoxycinnamate	Hydroxycinnamoyl derivatives	84700	71500	55200	13300	8530	12700	27300	15900	20600	42500	44100	41000
Mul0290	Positive	271	137.1	6.67	270.089	[M+H]+	Medicarpin	Hydroxycinnamoyl derivatives	4740	2780	14600	38800	17400	18100	22200	17400	18100	13800	14900	13700
Mul0302	Positive	179	138	7.3	178.099	[M+H]+	Methyleugenol	Hydroxycinnamoyl derivatives	23600	21900	19600	9	9	9	9	9	9	8670	7750	6490
Mul0454	Negative	665.1	323.4	2.19	666.1	[M-H]-	O-Caffeoyl maltotriose	Hydroxycinnamoyl derivatives	30000	18000	24900	9	9	9	9	771	1820	12700	12100	13500
Mul0245	Positive	149.1	131	4.25	148.052	[M+H]+	p-Coumaraldehyde	Hydroxycinnamoyl derivatives	159000	252000	18100	13500	21800	13100	13200	15100	43000	73400	71000	83400
Mul0518	Negative	163.047	119	3.71	164.047	[M-H]-	p-Coumaric acid	Hydroxycinnamoyl derivatives	2640000	3110000	920000	488000	844000	548000	491000	439000	402000	1310000	1240000	1220000
Mul0510	Negative	149	130.1	3.5	150.068	[M-H]-	p-Coumaryl alcohol	Hydroxycinnamoyl derivatives	10100	9	4490	18600	22200	18300	9290	10500	9130	8840	9100	6430
Mul0570	Negative	357.142	136.1	5.18	358.142	[M-H]-	Pinoresinol	Hydroxycinnamoyl derivatives	68400	31600	61700	50200	33800	44200	19100	15700	15600	38700	29400	35500
Mul0562	Negative	227.079	143.1	4.59	228.0786	[M-H]-	Resveratrol	Hydroxycinnamoyl derivatives	82300	100000	52400	614000	330000	440000	22700	24000	33700	168000	161000	151000
Mul0532	Negative	223.069	149.1	3.89	224.069	[M-H]-	Sinapic acid	Hydroxycinnamoyl derivatives	9	9	9	151000	172000	146000	9	26500	30900	49300	43500	48400
Mul0557	Negative	207.1	177.1	4.43	208.074	[M-H]-	Sinapinaldehyde	Hydroxycinnamoyl derivatives	19900	20700	32700	173000	119000	143000	47300	56100	34800	67000	59300	60400
Mul0522	Negative	209	179.1	3.73	210.089	[M-H]-	Sinapyl alcohol	Hydroxycinnamoyl derivatives	9	9	9	148000	126000	144000	9	9	9	34600	41900	28100
Mul0539	Negative	181.1	151.1	3.99	182.1	[M-H]-	Syringaldehyde	Hydroxycinnamoyl derivatives	7780	8390	14900	12300	6250	15100	13800	7550	6210	9090	12600	11000
Mul0482	Negative	371.1	209.2	2.8	372.142	[M-H]-	Syringin	Hydroxycinnamoyl derivatives	154000	146000	161000	183000	183000	173000	55600	127000	141000	155000	150000	132000
Mul0278	Positive	133.058	115	5.83	132.0575	[M+H]+	trans-cinnamaldehyde	Hydroxycinnamoyl derivatives	5670	6480	6790	11400	6950	15100	15200	12800	6630	9840	13400	8560
Mul0502	Negative	167	152.1	3.26	168	[M-H]-	Vanillic acid	Hydroxycinnamoyl derivatives	29900	28400	39700	23100	19400	28000	25900	21300	82600	31900	34800	29900
Mul0214	Positive	219.1	189.5	3.68	218.1	[M+H]+	5-Methoxy-N,N-dimethyltryptamine	Tryptamine derivatives	169000	145000	111000	132000	109000	122000	28200	30400	21600	86400	83800	85800
Mul0156	Positive	161.1	144.2	2.75	160.1	[M+H]+	L-Tryptamine	Tryptamine derivatives	77500	99600	227000	647000	338000	661000	60800	34900	36200	207000	193000	225000
Mul0175	Positive	219	160.1	3.06	218.106	[M+H]+	N-Acetyl-5-hydroxytryptamine	Tryptamine derivatives	28100	21400	33400	37200	35200	33900	54300	69100	71200	53000	39300	31800
Mul0216	Positive	485.1	147.5	3.68	484.1	[M+H]+	N-hexosyl-p-coumaroyl serotonin	Tryptamine derivatives	41500	35600	44500	8180	7890	4900	16700	14900	5740	53600	34100	54100
Mul0103	Positive	177.2	160.5	1.44	176.2	[M+H]+	N-hydroxy tryptamine	Tryptamine derivatives	69300	26100	30200	490000	429000	742000	37300	106000	48700	113000	96300	109000
Mul0110	Positive	177	160.2	1.66	176.095	[M+H]+	serotonin	Tryptamine derivatives	5680000	7960000	6010000	22500000	20700000	24200000	4940000	10400000	9090000	12700000	12900000	13300000
Mul0468	Negative	359.2	179.1	2.5	360.129	[M-H]-	Aminophylline	Alkaloids	17700	17300	18300	9	9	9	35500	32500	20800	16700	16800	11400
Mul0058	Positive	118.1	118.1	0.81	117.079	[M+H]+	Betaine	Alkaloids	165000	435000	165000	84300	90900	57800	74000	62300	122000	97100	87700	137000
Mul0513	Negative	433.121	433	3.6	434.121	[M-H]-	Isohemiphloin	Alkaloids	112000	64500	66400	20900	14900	16300	22400	22000	20500	53300	48900	43500
Mul0074	Positive	86.1	56	1.17	85.1	[M+H]+	Piperidine	Alkaloids	304000	126000	588000	636000	773000	533000	1990000	1850000	1940000	1200000	1120000	1120000
Mul0050	Positive	138	137.9	0.8	137.048	[M+H]+	Trigonelline	Alkaloids	6860000	11200000	8480000	6750000	6430000	7500000	3380000	2900000	2980000	4320000	5090000	5250000
Mul0396	Negative	213.1	78.9	0.97	214.1	[M-H]-	2-Deoxyribose 1-phosphate	Carbohydrates	75800	47400	28300	113000	198000	111000	732000	1010000	983000	428000	450000	435000
Mul0387	Negative	177.14	70.8	0.82	178.048	[M-H]-	D-(+)-Glucono-1,5-lactone	Carbohydrates	167000	155000	69100	83100	97300	108000	28700	37800	36000	54000	71800	53400
Mul0376	Negative	179	70.9	0.77	180.063	[M-H]-	D(+)-Glucose	Carbohydrates	8170000	7860000	3430000	3210000	4750000	3730000	2310000	1900000	2080000	3320000	4100000	3340000
Mul0035	Positive	505.169	127	0.77	504.169	[M+H]+	D(+)-Melezitose	Carbohydrates	64300	49200	106000	54300	90100	74000	97800	73400	75300	60700	74500	66800
Mul0372	Negative	649.1	503.3	0.77	650.1	[M-H]-	D(+)-Melezitose O-rhamnoside	Carbohydrates	37700	34000	63300	30000	64900	21100	120000	108000	161000	104000	72800	105000
Mul0379	Negative	341	58.8	0.78	342.11621	[M-H]-	D-(+)-Sucrose	Carbohydrates	7370000	5320000	7710000	4720000	4270000	5190000	7280000	9030000	7240000	8070000	5070000	5110000
Mul0421	Negative	259	78.8	1.23	260.03	[M-H]-	D-Fructose 6-phosphate	Carbohydrates	242000	241000	263000	469000	688000	462000	1330000	1220000	1220000	845000	762000	746000
Mul0384	Negative	193.141	72.9	0.8	194.043	[M-H]-	D-glucoronic acid	Carbohydrates	217000	224000	129000	446000	769000	430000	988000	1490000	1790000	887000	812000	907000
Mul0398	Negative	259	96.9	0.99	260.03	[M-H]-	D-Glucose 6-phosphate	Carbohydrates	1000000	751000	1120000	5600000	5050000	4910000	5680000	4440000	4300000	4410000	4550000	4030000
Mul0385	Negative	149	58.9	0.81	150.053	[M-H]-	DL-Arabinose	Carbohydrates	1410000	1140000	976000	409000	475000	497000	243000	290000	269000	444000	451000	450000
Mul0424	Negative	289	78.9	1.25	290.04	[M-H]-	D-Sedoheptuiose 7-phosphate	Carbohydrates	99600	69900	42400	272000	1080000	9	155000	132000	144000	443000	262000	480000
Mul0402	Negative	289.1	97	1.16	290.1	[M-H]-	Glucarate O-Phosphoric acid	Carbohydrates	235000	170000	79800	9	2850000	9	315000	192000	233000	713000	683000	749000
Mul0004	Positive	197.058	153	0.59	196.0583	[M+H]+	Gluconic acid	Carbohydrates	42700	115000	50400	27400	164000	39000	5120	4260	37800	57000	58500	59900
Mul0007	Positive	180	84.2	0.61	179	[M+H]+	Glucosamine	Carbohydrates	124000	571000	354000	334000	337000	337000	194000	188000	201000	205000	248000	354000
Mul0395	Negative	163	70.8	0.85	164.069	[M-H]-	L-Fucose	Carbohydrates	20000	17000	22600	14200	18600	17400	11000	19800	9	26900	19300	9340
Mul0380	Negative	177.14	74.8	0.78	178.048	[M-H]-	L-Gulonic-γ-lactone	Carbohydrates	42200	54100	9	9	24000	9	9	13500	15600	16300	20400	26000
Mul0373	Negative	665.1	485.3	0.77	666.1	[M-H]-	Maltotetraose	Carbohydrates	15100	24200	18700	14200	18300	19700	19600	13900	20100	19200	15500	15400
Mul0063	Positive	222	138.1	0.83	221.09	[M+H]+	N-Acetyl-D-glucosamine	Carbohydrates	87800	71300	74400	183000	179000	162000	59400	56400	52600	69000	78300	87000

Mul0390	Negative	421.1	241.2	0.83	422.1	[M-H]-	Trehalose 6-phosphate	Carbohydrates	60200	50300	80500	75300	87000	77000	76100	72500	81800	85700	93500	82300
Mul0288	Positive	319.1	301.9	6.63	318.1	[M+H]+	Phytocassane C	Terpenoids	9	9	9	213000	50500	159000	9	9	9	23100	25000	24800
Mul0321	Positive	301.2	133.1	8.15	300.209	[M+H]+	4-Oxoretinol	Vitamins	2880	4560	9	8510	6740	6430	13200	3880	4270	5010	5720	6560
Mul0358	Positive	287.2	173.2	11.2	286.23	[M+H]+	All-trans-13,14-dihydroretinol	Vitamins	4410	1530	3610	5750	5010	6160	3620	4210	3530	4980	4190	4190
Mul0183	Positive	245.09	227.1	3.21	244.088	[M+H]+	Biotin	Vitamins	65200	60200	89100	132000	119000	144000	444000	250000	631000	167000	205000	175000
Mul0130	Positive	220.2	202.7	2.19	219.2	[M+H]+	D-Pantothenic acid	Vitamins	8590000	7200000	11300000	8670000	8520000	9830000	12000000	10800000	9190000	9810000	9660000	9520000
Mul0394	Negative	175	87	0.84	176.12	[M-H]-	L-ascorbate	Vitamins	593000	388000	386000	348000	156000	323000	144000	136000	475000	184000	200000	186000
Mul0104	Positive	123.1	80.3	1.47	122.1	[M+H]+	Niacinamide	Vitamins	50500	82100	61300	53400	33400	32100	63100	59100	45000	43500	45600	48100
Mul0205	Positive	138.05	78	3.58	137.048	[M+H]+	Nicotinic Acid Methyl Ester (Methyl Nicotinate)	Vitamins	9	7370	9	9	9	9	8850	10900	15800	7570	8430	9250
Mul0422	Negative	155	111	1.24	156.017	[M-H]-	Orotic acid	Vitamins	70800	44800	99000	9	9	9	419000	364000	247000	141000	161000	147000
Mul0157	Positive	279.13	149.2	2.78	278.13	[M+H]+	Pantetheine	Vitamins	9	9	9	389000	655000	496000	9	9	9	207000	232000	208000
Mul0091	Positive	170.1	134.1	1.22	169.074	[M+H]+	Pyridoxine	Vitamins	404000	689000	308000	328000	365000	354000	98000	79900	95800	285000	294000	307000
Mul0096	Positive	250	134	1.25	249.04	[M+H]+	Pyridoxine 5'-phosphate	Vitamins	9	3010	9	9	9	9	6290	4420	10100	4760	3110	6500
Mul0102	Positive	494.1	332.8	1.39	493.1	[M+H]+	Pyridoxine di-O-hexoside	Vitamins	10200	13100	11200	4560	5370	9	9	4480	4030	6680	6550	6640
Mul0171	Positive	508.1	152.5	2.98	507.1	[M+H]+	Pyridoxine O-feruloyl hexoside	Vitamins	9	9	9	28500	18300	25800	18700	14400	9510	11700	12600	9450
Mul0076	Positive	332.1	314.7	1.18	331.1	[M+H]+	Pyridoxine O-glucoside	Vitamins	1200000	1150000	1400000	686000	1110000	706000	873000	688000	460000	849000	939000	961000
Mul0495	Negative	375	255.2	3.09	376.138	[M-H]-	Riboflavin	Vitamins	42200	47900	57800	25900	32000	22200	41800	30300	37600	46400	40700	43100
Mul0054	Positive	265.1	140.4	0.81	264.1	[M+H]+	Thiamine	Vitamins	13800	8670	13500	362000	227000	340000	12900	13200	8690	26200	28400	28900
Mul0275	Positive	149.2	107	5.58	148.052	[M+H]+	3,4-Dihydrocoumarin	Coumarins	29400	38200	6470	9	9	9	9	9	8250	11400	13300	13400
Mul0504	Negative	485.1	323.3	3.3	486.1	[M-H]-	4-hydroxycoumarin di-glucoside	Coumarins	9	5010	9	5920	9	6790	6630	7240	6990	5140	6790	3600
Mul0252	Positive	177.047	121	4.46	176.0473	[M+H]+	4-Methylumbelliferone	Coumarins	65000	50700	49200	97900	93100	78300	106000	108000	88700	88300	67500	71700
Mul0154	Positive	353.1	272.8	2.73	352.1	[M+H]+	6,7-dihydroxycoumarin 6-O-quinic acid	Coumarins	62400	142000	109000	60900	39500	49400	166000	128000	128000	86100	67900	95500
Mul0461	Negative	351.1	132.6	2.41	352.1	[M-H]-	6,7-dihydroxycoumarin 7-O-quinic acid	Coumarins	972000	1470000	1280000	300000	280000	264000	1550000	1230000	1060000	962000	621000	787000
Mul0242	Positive	177	121	4.15	176.047	[M+H]+	6-Hydroxy-4-methylcoumarin	Coumarins	39800	13300	22900	52700	52000	50800	61800	77700	62500	45000	37600	37700
Mul0196	Positive	209.2	194.1	3.45	208.037	[M+H]+	6-Methoxy-7,8-DihydroxyCoumarin	Coumarins	146000	129000	195000	9	9	9	251000	256000	193000	124000	137000	107000
Mul0190	Positive	179.03	179	3.35	178.027	[M+H]+	Daphnetin	Coumarins	29200	43000	13700	14700	32200	32500	30000	67900	71700	40800	38000	32100
Mul0498	Negative	177	133.1	3.17	178.027	[M-H]-	Esculetin (6,7-dihydroxycoumarin)	Coumarins	132000	154000	83300	94100	147000	98900	132000	252000	215000	153000	144000	132000
Mul0462	Negative	703.4	351.3	2.42	704.4	[M-H]-	Esculetin O-quinacyl esculetin O-quinic acid	Coumarins	22100	63100	34100	2590	9	4400	67200	50700	45000	23400	13100	13300
Mul0149	Positive	341	179.1	2.58	340.079	[M+H]+	Esculin (6,7-Dihydroxycoumarin-6-glucoside)	Coumarins	1450000	1630000	3300000	409000	318000	450000	5740000	3880000	4290000	917000	954000	924000
Mul0234	Positive	193.1	178.1	4	192.042	[M+H]+	Scopoletin (7-Hydroxy-5-methoxycoumarin)	Coumarins	160000	79100	92900	82000	76400	76300	235000	265000	223000	145000	127000	128000
Mul0047	Positive	256.2	124	0.8	255.2	[M+H]+	Nicotinate ribonucleoside	Nicotinic acid derivatives	8370000	8690000	4880000	4820000	5460000	5100000	3340000	3180000	3260000	4570000	4590000	4620000
Mul0409	Negative	122	77.9	1.19	123	[M-H]-	Nicotinic acid	Nicotinic acid derivatives	224000	170000	82800	9	97200	25100	404000	378000	420000	194000	200000	191000
Mul0118	Positive	286	124.3	1.9	285	[M+H]+	Nicotinic acid-hexoside	Nicotinic acid derivatives	30600	28400	30600	19600	28700	23400	70200	92900	95200	58800	45700	50000
Mul0552	Negative	269	225	4.37	270.053	[M-H]-	2'-Hydroxydaidzein	Isoflavone	4840	5290	4040	9	9	9	10500	13300	14500	7260	6320	7280
Mul0258	Positive	287	217.1	4.79	286.048	[M+H]+	2'-Hydroxygenistein	Isoflavone	1790000	1960000	4500000	248000	516000	280000	163000	123000	1210000	1110000	1140000	1160000
Mul0247	Positive	271	215.2	4.32	270.053	[M+H]+	6-Hydroxydaidzein	Isoflavone	12300	14900	9	6710	8270	5270	3730	5060	3510	6580	10900	4630
Mul0296	Positive	285	270.2	7	284.069	[M+H]+	Biochanin A	Isoflavone	13200	6800	76000	9	9	9	9	9	9	10000	9050	12300
Mul0257	Positive	255.058	199.1	4.75	254.0579	[M+H]+	Daidzein	Isoflavone	9	8930	16800	5730	9	4060	12900	13600	18500	8860	8410	7840
Mul0254	Positive	431.13	267.7	4.54	430.126	[M+H]+	Formononetin 7-O-glucoside (Ononin)	Isoflavone	9	9	9	6760	6010	6660	19000	14600	19600	7920	11000	10100
Mul0226	Positive	433	270.9	3.84	432.106	[M+H]+	Genistein 7-O-Glucoside (Genistin)	Isoflavone	79600	68400	164000	9	9	9	241000	240000	326000	116000	133000	114000
Mul0564	Negative	285.048	257.1	4.83	286.048	[M-H]-	Orobol (5,7,3',4'-tetrahydroxyisoflavone)	Isoflavone	8930	10500	14100	160000	130000	93500	4640	9360	15700	70900	50900	72300
Mul0168	Positive	192.06	114.9	2.95	191.058	[M+H]+	5-Hydroxyindole-3-acetic acid	Indole derivatives	11300	9130	10700	9790	8550	12500	9	9970	3160	12500	9110	9080
Mul0144	Positive	118	58.2	2.41	117	[M+H]+	Indole	Indole derivatives	18800	10200	48900	30500	34900	20500	136000	98800	114000	32200	46800	53500
Mul0249	Positive	146.1	118.1	4.36	145.053	[M+H]+	Indole-3-carboxaldehyde	Indole derivatives	23800	27500	45300	35700	32400	34800	76900	37900	40900	33700	39100	36200
Mul0543	Negative	160.05	116	4.07	161.0477	[M-H]-	Indole-5-carboxylic acid	Indole derivatives	38000	25100	32500	28000	26400	31800	67400	32100	51300	42900	40400	39300
Mul0139	Positive	206.1	147.5	2.39	205.1	[M+H]+	Methoxyindoleacetic acid	Indole derivatives	207000	76000	502000	405000	417000	359000	1920000	1680000	1500000	730000	683000	716000
Mul0428	Negative	147.07	58.8	1.36	148.074	[M-H]-	(Rs)-Mevalonic acid	Organic acids	70800	71600	9	9	215000	9	759000	784000	1120000	428000	401000	338000
Mul0509	Negative	131.08	68.9	3.45	132.079	[M-H]-	(S)-(-)-2-Hydroxyisocaproic acid	Organic acids	57000	40600	34100	23900	32000	44800	53100	49400	124000	62100	48400	39800
Mul0523	Negative	164.04	92	3.76	165.043	[M-H]-	2-(Formylamino)benzoic acid	Organic acids	256000	329000	91800	62600	77500	64000	69400	44300	49400	147000	125000	125000
Mul0486	Negative	153.2	109	2.88	154.027	[M-H]-	2,3-Dihydroxybenzoic acid	Organic acids	215000	169000	146000	569000	552000	518000	217000	229000	245000	838000	499000	489000
Mul0365	Negative	124	79.7	0.75	125.015	[M-H]-	2-Aminoethanesulfonic acid	Organic acids	4920	4860	5950	10100	11700	11400	5290	5600	7770	9800	7410	9870
Mul0480	Negative	175.1	131.3	2.73	176.1	[M-H]-	2-Isopropylmalate	Organic acids	3460000	2280000	3310000	3500000	4190000	3590000	7940000	8450000	8450000	5630000	4970000	4670000
Mul0474	Negative	145.06	101.1	2.58	146.058	[M-H]-	2-Methylglutaric acid	Organic acids	437000	310000	395000	210000	220000	228000	576000	577000	532000	341000	316000	301000
Mul0450	Negative	131.042	86.9	2.15	132.0423	[M-H]-	2-Methylsuccinic acid	Organic acids	2880000	2220000	2270000	2650000	2120000	2550000	3940000	4210000	4310000	3100000	2880000	2920000
Mul0088	Positive	124.03	77.8	1.21	123.032	[M+H]+	2-Picolinic acid	Organic acids	29200	51100	18600	14100	28500	24900	22800	24000	27300	29900	17100	25700

Mul0473	Negative	167.1	139	2.56	168.042	[M-H]-	3,4-Dihydroxybenzeneacetic acid	Organic acids	9	28700	25000	124000	109000	114000	9	31500	29300	72700	60400	42200
Mul0448	Negative	117.1	99.9	2.06	118.063	[M-H]-	3-Hydroxy-3-methyl butyric acid	Organic acids	98100	96500	130000	139000	116000	71200	246000	285000	220000	124000	184000	137000
Mul0429	Negative	103.0473	103	1.39	104.0473	[M-H]-	3-Hydroxybutyrate	Organic acids	259000	206000	128000	9	104000	9	67400	84400	78300	110000	93000	88700
Mul0107	Positive	146.074	85.8	1.58	145.0739	[M+H]+	4-Acetamidobutyric acid	Organic acids	26300	9	28100	81900	152000	67700	39100	45100	54400	53600	58900	58200
Mul0280	Positive	151.068	78.8	5.88	150.0681	[M+H]+	4-Ethylbenzoic acid	Organic acids	7170	8520	4470	13300	8540	8380	10700	12600	9280	9970	8130	9180
Mul0062	Positive	146.085	85.9	0.82	145.0851	[M+H]+	4-Guanidinobutyric acid	Organic acids	171000	221000	650000	255000	246000	212000	316000	278000	253000	208000	250000	256000
Mul0490	Negative	137.03	92.5	2.98	138.032	[M-H]-	4-Hydroxybenzoic acid	Organic acids	2430000	1620000	1070000	390000	381000	341000	166000	299000	296000	873000	814000	767000
Mul0025	Positive	132.1	86.2	0.75	131.1	[M+H]+	5-Aminolevulinate	Organic acids	761000	564000	1750000	2250000	2680000	1750000	6210000	6560000	6470000	3180000	3490000	4350000
Mul0067	Positive	132.095	76.9	0.93	131.0946	[M+H]+	6-Aminocaproic acid	Organic acids	181000	128000	381000	483000	590000	469000	1810000	1670000	1560000	783000	758000	835000
Mul0464	Negative	145.0579	80.9	2.43	146.0579	[M-H]-	Adipic acid	Organic acids	419000	315000	413000	138000	125000	146000	543000	540000	517000	300000	304000	225000
Mul0414	Negative	145	80.8	1.22	146.022	[M-H]-	A-Ketoglutaric acid	Organic acids	879000	847000	971000	390000	303000	382000	379000	262000	203000	667000	663000	632000
Mul0416	Negative	118	73.9	1.22	119.022	[M-H]-	Aminomalonic acid	Organic acids	228000	276000	292000	9	9	9	977000	1230000	1070000	462000	414000	406000
Mul0389	Negative	289.1	191.2	0.83	290.1	[M-H]-	Argininosuccinate	Organic acids	9330	5830	8960	63900	65000	74600	61100	44100	38000	45800	58600	47900
Mul0246	Positive	189.1049	97	4.27	188.1049	[M+H]+	Azelaic acid	Organic acids	44900	39200	34400	42800	60600	49700	89300	85500	192000	63800	67800	52800
Mul0549	Negative	187.1049	125.1	4.27	188.1049	[M-H]-	Azelaic Acid	Organic acids	840000	870000	668000	1050000	1180000	1090000	1330000	1380000	2980000	1300000	1200000	1270000
Mul0368	Negative	173.016	137	0.75	174.016	[M-H]-	Cis-Aconitic acid	Organic acids	47400	9830	17000	9	17600	9	18200	29000	24900	8110	8470	9950
Mul0399	Negative	147	85	1	148	[M-H]-	Citramalate	Organic acids	130000	125000	146000	54800	204000	66100	473000	489000	496000	246000	209000	242000
Mul0386	Negative	191	131	0.81	192.027	[M-H]-	Citric acid	Organic acids	20700000	24900000	33400000	36000000	29000000	35700000	24800000	24000000	24600000	38200000	39000000	39500000
Mul0377	Negative	130.0695	88	0.77	131.07	[M-H]-	Creatine	Organic acids	89100	74700	65700	34300	77700	46400	200000	185000	188000	71000	82800	110000
Mul0437	Negative	153	78.9	1.72	154	[M-H]-	Diethyl phosphate	Organic acids	41300	67600	34500	480000	110000	187000	559000	438000	281000	257000	237000	221000
Mul0284	Positive	231.152	149.1	5.95	230.152	[M+H]+	Dodecanedioic aicd	Organic acids	18200	16400	21300	8060	11500	11400	37300	30800	24000	20200	18400	18400
Mul0133	Positive	477.2	258.1	2.23	476.168	[M+H]+	D-Pantothenic dcid	Organic acids	44200	43600	57700	117000	115000	118000	150000	124000	99100	87000	104000	87700
Mul0408	Negative	165.05	74.9	1.18	166.048	[M-H]-	D-Xylonic acid	Organic acids	563000	441000	545000	99000	157000	105000	563000	573000	467000	264000	260000	218000
Mul0447	Negative	131.04	86.9	2.02	132.042	[M-H]-	ethylmalonate	Organic acids	16500	12100	9	16500	22500	13200	19700	24000	18800	20100	12100	14700
Mul0423	Negative	115	97.9	1.24	116.011	[M-H]-	Fumaric acid	Organic acids	20900	13800	31100	20900	32500	11100	186000	151000	149000	76500	82500	82700
Mul0440	Negative	131.0423	86.9	1.89	132.0423	[M-H]-	Glutaric acid	Organic acids	344000	389000	182000	350000	484000	327000	465000	624000	626000	485000	374000	411000
Mul0455	Negative	167.1	122.1	2.2	168.042	[M-H]-	Homogentisic acid	Organic acids	9	9	17000	122000	125000	136000	9	41200	43600	60000	53600	44500
Mul0476	Negative	188.0426	144.1	2.62	189.0426	[M-H]-	Kynurenic acid	Organic acids	38900	34700	25700	60200	44600	45800	206000	169000	123000	69500	68300	59000
Mul0418	Negative	133	70.8	1.23	134.022	[M-H]-	L(-)-Malic acid	Organic acids	4370000	4680000	5200000	9	9	9	31100000	36700000	35000000	19700000	17900000	14600000
Mul0371	Negative	145.06	74	0.77	146.06	[M-H]-	Methylglutaric acid	Organic acids	30900	22400	42600	12100	42300	9110	21600	23000	28100	31000	28600	24600
Mul0434	Negative	117.02	100	1.55	118.0266087	[M-H]-	Methylmalonic acid	Organic acids	7720000	6590000	7350000	22800000	16400000	22100000	19800000	21900000	17600000	16800000	15100000	16100000
Mul0066	Positive	98.9	81.1	0.89	97.9	[M+H]+	Phosphoric acid	Organic acids	241000	280000	235000	679000	695000	692000	429000	351000	490000	336000	378000	436000
Mul0170	Positive	167.027	149.1	2.97	166.0266	[M+H]+	Phthalic acid	Organic acids	320000	248000	302000	25600	27100	37900	224000	170000	142000	99900	94000	91600
Mul0400	Negative	173.053	92.9	1.05	174.0528	[M-H]-	Shikimic acid	Organic acids	168000	110000	110000	177000	191000	178000	133000	101000	88000	132000	145000	152000
Mul0520	Negative	173.1	83	3.71	174.089	[M-H]-	Suberic acid	Organic acids	37100	35900	39200	49000	64000	60400	61900	65400	46800	59200	51200	54300
Mul0427	Negative	117.03	99.9	1.31	118.027	[M-H]-	Succinic acid	Organic acids	6660000	7280000	8240000	9	4560000	9	26700000	31800000	28800000	12500000	12000000	10700000
Mul0355	Positive	556.28	371.2	10.06	555.284	[M+H]+	Taurocholic acid	Organic acids	43100	49400	44000	40000	44000	44200	38400	43000	36600	42000	41600	48200
Mul0487	Negative	165.027	121.1	2.91	166.0266	[M-H]-	Terephthalic acid	Organic acids	1050000	626000	549000	2460000	2030000	2250000	1950000	1910000	1770000	1530000	1420000	1340000
Mul0359	Negative	141.03	58.7	0.63	142.027	[M-H]-	trans,trans-Muconic acid	Organic acids	534000	436000	523000	719000	544000	505000	438000	718000	521000	502000	680000	508000
Mul0008	Positive	175	98.9	0.61	174.016	[M+H]+	trans-Citridic acid	Organic acids	234000	443000	263000	286000	285000	391000	293000	261000	242000	334000	338000	332000
Mul0128	Positive	206.1	132.4	2.15	205.1	[M+H]+	Xanthurenic acid	Organic acids	10200	6420	6480	8700	6230	4640	9	6940	6450	8620	5430	5790
Mul0435	Negative	103.047	56.8	1.55	104.047	[M-H]-	α-Hydroxyisobutyric acid	Organic acids	9100	13600	13700	9950	10100	8750	16600	16600	24400	18800	12200	12000
Mul0019	Positive	104.06	87	0.69	103.063	[M+H]+	γ-aminobutyric acid	Organic acids	550000	453000	684000	150000	180000	63900	595000	581000	626000	435000	401000	444000
Mul0537	Negative	575	285.3	3.94	576.1268	[M-H]-	Procyanidin A2	Proanthocyanidins	9	9	9	7500	12100	11500	9	7790	7340	4350	2780	5760
Mul0493	Negative	577	407.3	3.08	578.1424	[M-H]-	Procyanidin B2	Proanthocyanidins	9	9	9	60000	56200	89900	19000	148000	129000	21900	19100	19500
Mul0485	Negative	577.1	407.3	2.87	578.1424	[M-H]-	Procyanidin B3	Proanthocyanidins	9	9	9	121000	88900	153000	12000	86000	81800	72200	76700	68700
Mul0277	Positive	412.3	184.7	5.64	411.3	[M+H]+	LysoPC 10:0	Lipids_Glycerophospholipids	9	9	9	4480	7390	5800	4310	6820	8930	2810	3010	2590
Mul0286	Positive	440.1	422.7	6.58	439.1	[M+H]+	LysoPC 12:1	Lipids_Glycerophospholipids	9	9	9	21100	36400	12400	33800	78700	47500	23700	23800	23500
Mul0313	Positive	468.1	450.5	7.57	467.1	[M+H]+	LysoPC 14:0	Lipids_Glycerophospholipids	20800	17500	30500	840000	1180000	473000	1450000	3720000	2990000	1040000	1030000	951000
Mul0307	Positive	468.3	450.5	7.43	467.3	[M+H]+	LysoPC 14:0 (2n isomer)	Lipids_Glycerophospholipids	32200	23700	15000	96700	579000	74500	430000	944000	819000	295000	276000	265000
Mul0319	Positive	482.3	184.5	8.1	481.3	[M+H]+	LysoPC 15:0	Lipids_Glycerophospholipids	112000	82800	248000	365000	259000	257000	250000	423000	358000	220000	248000	204000
Mul0312	Positive	480.1	184.5	7.54	479.1	[M+H]+	LysoPC 15:1	Lipids_Glycerophospholipids	9	9	5560	8590	10200	4470	10600	13100	12500	7820	7180	6890
Mul0335	Positive	496.3	478.3	8.65	495.3	[M+H]+	LysoPC 16:0	Lipids_Glycerophospholipids	21300000	18700000	29200000	34600000	29000000	29200000	33900000	37000000	36400000	29300000	32300000	30900000
Mul0331	Positive	496.33	478.3	8.49	495.33	[M+H]+	LysoPC 16:0 (2n isomer)	Lipids_Glycerophospholipids	3670000	2970000	3150000	1680000	1920000	1070000	5100000	9490000	7680000	3100000	3470000	3180000
Mul0328	Positive	494.3	476.6	8.38	493.3	[M+H]+	LysoPC 16:1	Lipids_Glycerophospholipids	9	9	9	15800	16700	10000	29500	40800	39600	16500	19300	20700
Mul0315	Positive	494.3	184.6	7.71	493.3	[M+H]+	LysoPC 16:1 (2n isomer)	Lipids_Glycerophospholipids	17400000	15000000	22600000	565000	2890000	414000	1730000	2320000	1970000	8970000	8930000	8520000
Mul0300	Positive	492.3	184.5	7.17	491.3	[M+H]+	LysoPC 16:2 (2n isomer)	Lipids_Glycerophospholipids	972000	668000	2260000	47700	207000	26200	206000	256000	225000	537000	499000	465000
Mul0346	Positive	510.1	510.4	9.22	509.1	[M+H]+	LysoPC 17:0	Lipids_Glycerophospholipids	45600	29300	115000	207000	99800	135000	116000	213000	110000	74000	95300	83200
Mul0349	Positive	524.36	507.4	9.57	523.36	[M+H]+	LysoPC 18:0	Lipids_Glycerophospholipids	68600	60000	102000	197000	153000	137000	298000	503000	315000	110000	168000	139000
Mul0342	Positive	524.1	507.4	8.94	523.1	[M+H]+	LysoPC 18:0 (2n isomer)	Lipids_Glycerophospholipids	101000	96900	151000	55500	274000	40600	209000	340000	225000	174000	159000	161000
Mul0343	Positive	522.1	125.5	8.95	521.1	[M+H]+	LysoPC 18:1	Lipids_Glycerophospholipids	116000	108000	155000	108000	75800	72800	130000	227000	212000	121000	111000	101000
Mul0336	Positive	522.4	184.5	8.76	521.4	[M+H]+	LysoPC 18:1 (2n isomer)	Lipids_Glycerophospholipids	13800000	14000000	20200000	7970000	29300000	6930000	25200000	32400000	26600000	23400000	23000000	21200000
Mul0326	Positive	520.3	502.3	8.27	519.3	[M+H]+	LysoPC 18:2	Lipids_Glycerophospholipids	296000	277000	514000	2230000	1730000	1450000	5600000	9140000	8250000	3130000	2910000	2890000
Mul0318	Positive	520	125	8.08	519	[M+H]+	LysoPC 18:2 (2n isomer)	Lipids_Glycerophospholipids	1550000	970000	3340000	4510000	17200000	3610000	22200000	27400000	23300000	16300000	15300000	14400000
Mul0334	Positive	518.3	459.6	8.65	517.3	[M+H]+	LysoPC 18:3	Lipids_Glycerophospholipids	1580000	1670000	2570000	3710000	2720000	2580000	3380000	5150000	4330000	2930000	3050000	3230000
Mul0311	Positive	518.2	500.3	7.54	517.2	[M+H]+	LysoPC 18:3 (2n isomer)	Lipids_Glycerophospholipids	26700000	26900000	24700000	14300000	25900000	12700000	26700000	26800000	25700000	26800000	26200000	26500000

Mul0289	Positive	538.1	184.5	6.66	537.1	[M+H]+	LysoPC 19:0	Lipids_Glycerophospholipids	6050	4120	21000	9190	6950	6750	9	9460	7400	10200	8850	9110
Mul0354	Positive	550.37	245.1	10.03	549.37	[M+H]+	LysoPC 20:1	Lipids_Glycerophospholipids	56200	65400	79200	16400	73100	16400	127000	184000	163000	80600	102000	88500
Mul0352	Positive	550.37	245.1	9.81	549.37	[M+H]+	LysoPC 20:1 (2n isomer)	Lipids_Glycerophospholipids	55900	66800	84500	20200	83000	19000	124000	206000	176000	79400	98800	89000
Mul0341	Positive	544.4	544.4	8.89	543.4	[M+H]+	LysoPC 20:4	Lipids_Glycerophospholipids	392000	568000	694000	277000	1440000	263000	947000	1580000	1370000	971000	932000	1030000
Mul0309	Positive	426.3	285.8	7.51	425.3	[M+H]+	LysoPE 14:0	Lipids_Glycerophospholipids	4970	6040	7120	206000	253000	163000	372000	881000	678000	227000	274000	274000
Mul0581	Negative	424.2	285.8	7.37	425.2	[M-H]-	LysoPE 14:0 (2n isomer)	Lipids_Glycerophospholipids	5390	8080	4470	16900	41600	20300	53400	105000	103000	34700	33300	32200
Mul0588	Negative	452	255.3	8.57	453	[M-H]-	LysoPE 16:0	Lipids_Glycerophospholipids	6300000	6650000	9180000	19600000	12800000	15000000	16300000	30900000	22300000	13700000	15900000	14100000
Mul0587	Negative	452.3	255.3	8.4	453.3	[M-H]-	LysoPE 16:0 (2n isomer)	Lipids_Glycerophospholipids	381000	394000	275000	386000	309000	338000	1290000	2650000	2250000	652000	788000	701000
Mul0350	Positive	482.1	341.9	9.69	481.1	[M+H]+	LysoPE 18:0	Lipids_Glycerophospholipids	3240	3050	5360	31600	12700	23700	7270	15700	10700	7670	9340	8150
Mul0339	Positive	482.1	314	8.82	481.1	[M+H]+	LysoPE 18:0 (2n isomer)	Lipids_Glycerophospholipids	7840	4930	27800	10800	8260	7070	15100	17300	10800	9130	9540	7550
Mul0591	Negative	478.1	281.4	8.87	479.1	[M-H]-	LysoPE 18:1	Lipids_Glycerophospholipids	541000	622000	694000	1450000	1450000	1470000	969000	1440000	1270000	900000	965000	949000
Mul0589	Negative	478.1	281.3	8.69	479.1	[M-H]-	LysoPE 18:1 (2n isomer)	Lipids_Glycerophospholipids	1520000	1950000	954000	1010000	5000000	1130000	2710000	3300000	2730000	2620000	2620000	2470000
Mul0323	Positive	478.6	337.9	8.2	477.6	[M+H]+	LysoPE 18:2 (2n isomer)	Lipids_Glycerophospholipids	31300	37300	47000	351000	433000	274000	1240000	1960000	1860000	599000	534000	526000
Mul0333	Positive	772.1	478.4	8.63	771.1	[M+H]+	PC 19:2/16:0	Lipids_Glycerophospholipids	1620	2990	5260	18900	7560	9550	23500	42200	33400	6000	14300	13400
Mul0306	Positive	694.1	335.8	7.41	693.1	[M+H]+	DGMG (18:1)	Lipids_Glycerolipids	85800	92300	73200	15400	25600	11400	110000	66200	65900	88000	77600	92300
Mul0316	Positive	696.4	337.8	7.92	695.4	[M+H]+	DGMG (18:2) isomer1	Lipids_Glycerolipids	53300	80400	73200	46000	95500	41200	565000	507000	438000	215000	229000	206000
Mul0304	Positive	696.4	696.2	7.41	695.4	[M+H]+	DGMG (18:2) isomer2	Lipids_Glycerolipids	24800	28100	29200	3780	9540	3480	36600	18700	16800	23700	20200	23500
Mul0320	Positive	696.4	696.3	8.12	695.4	[M+H]+	DGMG (18:2) isomer3	Lipids_Glycerolipids	57500	71100	60900	46600	87900	39200	461000	390000	443000	206000	249000	208000
Mul0332	Positive	357.2	339.9	8.57	356.2	[M+H]+	MAG (18:1) isomer1	Lipids_Glycerolipids	138000	274000	88500	34800	81600	45800	159000	108000	120000	123000	127000	144000
Mul0337	Positive	357.2	339.9	8.79	356.2	[M+H]+	MAG (18:1) isomer2	Lipids_Glycerolipids	117000	247000	76600	11500	34600	10900	146000	90600	107000	108000	114000	130000
Mul0356	Positive	355.1	337.9	10.36	354.1	[M+H]+	MAG (18:2)	Lipids_Glycerolipids	7350000	1360000	7630000	11600000	16100000	12200000	2880000	7060000	4980000	7650000	7680000	7470000
Mul0325	Positive	355.1	263.8	8.27	354.1	[M+H]+	MAG (18:2) isomer1	Lipids_Glycerolipids	12300	22400	16700	12400	21500	9040	128000	76000	75600	50000	54700	65700
Mul0353	Positive	353.2	261.8	9.91	352.2	[M+H]+	MAG (18:3) isomer1	Lipids_Glycerolipids	8960000	8700000	8500000	4430000	8620000	4710000	1500000	3430000	2600000	5370000	5250000	5760000
Mul0322	Positive	353.1	261.8	8.16	352.1	[M+H]+	MAG (18:3) isomer2	Lipids_Glycerolipids	59300	54400	70400	33300	63000	37600	79100	64000	62600	52400	69300	70900
Mul0329	Positive	353.3	335.8	8.43	352.3	[M+H]+	MAG (18:3) isomer3	Lipids_Glycerolipids	1040000	1460000	1390000	333000	885000	274000	1840000	969000	905000	1100000	1180000	1240000
Mul0351	Positive	353.1	173.7	9.77	352.1	[M+H]+	MAG (18:3) isomer4	Lipids_Glycerolipids	194000	202000	179000	103000	189000	107000	32300	76400	62800	121000	109000	127000
Mul0305	Positive	353.3	261.8	7.41	352.3	[M+H]+	MAG (18:3) isomer5	Lipids_Glycerolipids	912000	1360000	1070000	184000	324000	138000	1470000	761000	830000	829000	920000	908000
Mul0297	Positive	351.4	277.8	7.05	350.4	[M+H]+	MAG (18:4) isomer2	Lipids_Glycerolipids	9	9	12400	58100	27600	56200	6660	6670	3750	13500	15100	15500
Mul0314	Positive	351	259.9	7.64	350	[M+H]+	MAG (18:4) isomer3	Lipids_Glycerolipids	42900	35800	49800	4430	14000	6310	14700	14600	20100	15000	15400	17200
Mul0344	Positive	534.4	262.9	9.04	533.4	[M+H]+	MGMG (18:2) isomer1	Lipids_Glycerolipids	17600	21400	15000	24300	62100	23300	402000	339000	328000	165000	139000	153000
Mul0340	Positive	534.1	338	8.87	533.1	[M+H]+	MGMG (18:2) isomer2	Lipids_Glycerolipids	1290	1450	1750	2110	4800	1920	27700	27500	20900	12200	10900	14900
Mul0594	Negative	295	277.5	9.61	296	[M-H]-	12,13-EODE	Lipids_Fatty acids	943000	38500	659000	381000	398000	424000	1050000	1030000	1360000	755000	658000	663000
Mul0583	Negative	293	193.2	7.66	294	[M-H]-	13-HOTrE(r)	Lipids_Fatty acids	59000	60700	66800	125000	90500	63300	119000	94400	81300	91300	85800	90800
Mul0584	Negative	311.1	293.4	7.71	312.1	[M-H]-	13-HPODE	Lipids_Fatty acids	132000	19000	186000	23400	29300	25500	382000	148000	282000	150000	131000	138000
Mul0585	Negative	309	291.5	7.98	310	[M-H]-	13-HpOTrE(r)	Lipids_Fatty acids	106000	8480	73000	9	9	9	88400	43600	54200	23600	28200	31000
Mul0330	Positive	277.216	157.4	8.45	276.216	[M+H]+	14,15-Dehydrocrepenynic acid	Lipids_Fatty acids	1880000	854000	1130000	259000	336000	303000	560000	489000	872000	757000	694000	692000
Mul0287	Positive	318.3	300.9	6.63	317.3	[M+H]+	4-Hydroxysphinganine	Lipids_Fatty acids	200000	137000	193000	13900000	3540000	10100000	554000	434000	287000	1640000	2030000	2160000
Mul0590	Negative	295	183.3	8.82	296	[M-H]-	9,10-EODE	Lipids_Fatty acids	3640000	477000	3630000	3070000	3680000	3950000	7400000	6820000	7690000	4710000	4040000	4390000
Mul0586	Negative	293	275.4	8.29	294	[M-H]-	9-HOTrE	Lipids_Fatty acids	611000	381000	258000	106000	107000	109000	185000	151000	219000	241000	217000	227000
Mul0582	Negative	309.1	291.4	7.39	310.1	[M-H]-	9-HpOTrE	Lipids_Fatty acids	74500	45200	56300	44600	43800	29800	57300	51200	61100	50400	52400	51100
Mul0345	Positive	295.2	179.5	9.17	294.2	[M+H]+	9-Hydroxy-(10E,12Z,15Z)-octadecatrienoic acid	Lipids_Fatty acids	1540000	284000	1950000	2090000	2390000	2320000	5630000	5610000	9420000	3270000	3320000	3420000
Mul0592	Negative	293.2	249.9	9.03	294.2	[M-H]-	9-KODE	Lipids_Fatty acids	400000	234000	120000	90600	78900	93700	164000	172000	245000	185000	161000	181000
Mul0593	Negative	199	181.3	9.52	200	[M-H]-	Lauric acid (C12:0)	Lipids_Fatty acids	5790	2920	2920	14300	39100	14100	37100	96100	75400	35600	30000	31600
Mul0347	Positive	279.2	149.4	9.55	278.2	[M+H]+	Octadeca-11E,13E,15Z-trienoic acid	Lipids_Fatty acids	1710000	1420000	1530000	3310000	2070000	2090000	3010000	2920000	2480000	2440000	2240000	2450000
Mul0317	Positive	277.2	137.5	7.94	276.2	[M+H]+	Octadecadien-6-ynoic acid	Lipids_Fatty acids	39800	19300	59900	11800	16100	14100	74900	47600	76700	36400	36900	34400
Mul0338	Positive	279.3	201.5	8.81	278.3	[M+H]+	Punicic acid	Lipids_Fatty acids	5190000	749000	4970000	5380000	5760000	6670000	10300000	9680000	11000000	6700000	6830000	6690000
Mul0357	Positive	279.2	201.5	10.47	278.2	[M+H]+	α-Linolenic acid	Lipids_Fatty acids	3350000	1990000	2210000	1690000	853000	1180000	1670000	2040000	2600000	2190000	1720000	2030000
Mul0566	Negative	263	153.2	4.93	264.136	[M-H]-	(+)-cis,trans-Abscisic acid (ABA)	Phytohormones	3170000	2430000	2850000	1520000	1450000	1540000	3930000	2920000	2480000	2430000	2290000	2560000
Mul0576	Negative	209	58.7	5.64	210.126	[M-H]-	(+)-Jasmonic acid (JA)	Phytohormones	47400	49900	76400	44300	28200	26900	143000	74000	196000	77800	75100	79100
Mul0299	Positive	317.2	271.2	7.16	316.168	[M+H]+	GIBBERELLIN A9	Phytohormones	3180	5080	9	6950	11000	13000	5550	3390	3630	5990	5670	6020
Mul0255	Positive	176.1	130.1	4.56	175.063	[M+H]+	Indole 3-acetic acid (IAA)	Phytohormones	42700	6720	11500	10700	10900	15400	24700	14700	20300	23300	23900	16600
Mul0108	Positive	348.1	187.7	1.6	347.1	[M+H]+	Kinetin 9-riboside	Phytohormones	124000	168000	238000	329000	265000	313000	92000	109000	106000	143000	150000	202000
Mul0283	Positive	190.1	130.1	5.93	189.079	[M+H]+	Methyl indole-3-acetate	Phytohormones	60500	6340	7300	9	9	9	23400	14000	12000	13400	14900	14200
Mul0301	Positive	225.1	133.1	7.23	224.1412	[M+H]+	Methyl jasmonate	Phytohormones	6810	4600	5990	7930	5630	5190	3660	7230	5690	7540	4840	7220
Mul0579	Negative	322.401	130.1	6.38	323.401	[M-H]-	N-[(-)-Jasmonoyl]-(L)-Isoleucine (JA-L-Ile)	Phytohormones	41600	81200	34800	59400	78800	48700	39500	40200	182000	72000	65400	67400
Mul0224	Positive	204.251	136.1	3.82	203.117	[M+H]+	N6-Isopentenyladenine (iP)	Phytohormones	3730	1850	11900	31500	18800	43800	12100	2560	8800	17700	13500	13200
Mul0544	Negative	137	92.8	4.07	138.032	[M-H]-	Salicylic acid (SA)	Phytohormones	32800000	40400000	29400000	361000	256000	212000	510000	315000	444000	15500000	15000000	14700000
Mul0444	Negative	299.1	137.2	1.96	300.1	[M-H]-	Salicylic acid O-glucoside	Phytohormones	20200000	12200000	16700000	596000	555000	537000	400000	392000	221000	7330000	7290000	8240000
Mul0132	Positive	382.2	220.6	2.23	381.2	[M+H]+	trans-zeatin N-glucoside	Phytohormones	321000	654000	583000	312000	315000	370000	147000	122000	145000	307000	263000	320000

Supplementary Table S2. Differentially accumulated metabolites between CS and HG2.

Index	log2_FC(case_mean/control_mean)	Pvalue	fdr	vip	Ion mode	Q1 (Da)	Q3 (Da)	Rt (min)	Molecular Weight (Da)	Ionization model	Compounds	Class
Mul0541	2.027337085	0.001034	0.006145	1.001731	Negative	433.121	122.9	4.05	434.1213	[M-H]-	Naringenin 7-O-glucoside (Prunin)	Flavanone
Mul0113	-2.398989558	0.001985	0.00935	1.012965	Positive	285.1	153.1	1.74	284.076	[M+H]+	Xanthosine	Nucleotide and its derivates
Mul0048	-2.355145872	0.016715	0.042795	1.02527	Positive	258.2	125.2	0.8	258.2	Protonated	sn-Glycero-3-phosphocholine	Cholines
Mul0331	-1.06788631	0.006553	0.021504	1.027456	Positive	496.33	478.3	8.49	495.33	[M+H]+	LysoPC 16:0 (2n isomer)	Lipids_Glycerophospholipids
Mul0121	-0.50865055	0.014593	0.038356	1.163724	Positive	166	120.1	1.96	165.079	[M+H]+	L-Phenylalanine	Amino acids
Mul0432	-3.243661038	0.006634	0.02155	1.257786	Negative	282.092	150.1	1.44	283.092	[M-H]-	Guanosine	Nucleotide and its derivates
Mul0077	10.56313869	0.00054	0.004168	1.406658	Positive	308	162	1.18	307.084	[M+H]+	Glutathione reduced form	Amino acid derivatives
Mul0508	0.49386719	0.00333	0.012761	1.435059	Negative	337.1	190.9	3.44	338.1	[M-H]-	3-O-p-Coumaroyl quinic acid	Quinate and its derivatives
Mul0467	-9.595809975	0.000638	0.004615	1.502298	Negative	771.1	609.5	2.47	772.1	[M-H]-	Luteolin O-hexosyl-O-hexosyl-O-hexoside	Flavone
Mul0501	1.539264337	0.008486	0.02585	1.514385	Negative	449.1	329.3	3.21	450.1	[M-H]-	Eriodictyol C-hexoside	Flavone C-glycosides
Mul0200	-3.266886731	0.002752	0.011118	1.547779	Positive	558.282	264.8	3.5	557.282	[M+H]+	N', N''-disinapoylspermidine	Phenolamides
Mul0511	-3.658848904	0.009483	0.028137	1.575244	Negative	121.04	91.9	3.58	122.037	[M-H]-	4-Hydroxybenzaldehyde	Benzoic acid derivatives
Mul0398	2.438216825	5.79E-05	0.001186	1.670083	Negative	259	96.9	0.99	260.03	[M-H]-	D-Glucose 6-phosphate	Carbohydrates
Mul0445	-18.89805373	0.001273	0.007201	1.681785	Negative	355.1	181.2	1.97	356.1	[M-H]-	Homovanilloyl quinic acid	Quinate and its derivatives
Mul0212	18.92728757	5.25E-05	0.001114	1.720114	Positive	611.2	299.8	3.67	610.2	[M+H]+	Luteolin 8-C-hexosyl-O-hexoside	Flavone C-glycosides
Mul0418	-19.00957108	3.98E-05	0.000946	1.771973	Negative	133	70.8	1.23	134.022	[M-H]-	L(-)-Malic acid	Organic acids
Mul0529	-5.180373951	0.000963	0.005779	1.803986	Negative	505.1	301.2	3.87	506.1	[M-H]-	Quercetin O-acetylhexoside	Flavonol
Mul0039	-1.227209438	0.006393	0.021097	1.818014	Positive	104.1	60.2	0.78	103.1	[M+H]+	Choline	Cholines
Mul0427	-2.282147941	0.02086	0.050782	1.855011	Negative	117.03	99.9	1.31	118.027	[M-H]-	Succinic acid	Organic acids
Mul0456	-0.272291303	0.048768	0.097209	1.894683	Negative	353.1	191.1	2.21	354.095	[M-H]-	1-O-Caffeoyl quinic acid	Quinate and its derivatives
Mul0477	-5.505257523	1.21E-05	0.000513	1.901403	Negative	341	179.1	2.62	342.132	[M-H]-	Coniferin	Hydroxycinnamoyl derivatives
Mul0546	19.39933109	0.000186	0.002245	2.020545	Negative	609.2	301.3	4.09	610.19	[M-H]-	Hesperetin 7-O-neohesperidoside (Neohesperidin)	Flavanone
Mul0269	-1.899748526	0.009051	0.027154	2.05525	Positive	273.1	137.1	5.3	272.069	[M+H]+	Butein	Flavanone
Mul0356	1.287980763	0.034145	0.073221	2.099873	Positive	355.1	337.9	10.36	354.1	[M+H]+	MAG (18:2)	Lipids_Glycerolipids
Mul0120	0.876953311	0.000924	0.0056	2.135517	Positive	167.1	121	1.95	166.1	[M+H]+	6-Methylmercaptopurine	Nucleotide and its derivates
Mul0260	2.899799198	0.01403	0.037039	2.15444	Positive	289.063	153.1	4.85	288.063	[M+H]+	Eriodictyol	Flavanone
Mul0588	1.098883608	0.018616	0.045882	2.223973	Negative	452	255.3	8.57	453	[M-H]-	LysoPE 16:0	Lipids_Glycerophospholipids
Mul0287	5.69939239	0.040924	0.084996	2.228317	Positive	318.3	300.9	6.63	317.3	[M+H]+	4-Hydroxysphinganine	Lipids_Fatty acids
Mul0173	19.69395676	0.000205	0.002245	2.237187	Positive	463.123	301.4	3	463.123	Protonated	Peonidin O-hexoside	Anthocyanins
Mul0135	-19.7232669	0.001073	0.006308	2.243324	Positive	465.1	303.1	2.3	465.1	Protonated	Delphinidin 3-O-glucoside (Mirtillin)	Anthocyanins
Mul0094	-1.812711101	0.012839	0.034809	2.289364	Positive	130	83.9	1.24	129.043	[M+H]+	5-oxoproline	Amino acid derivatives
Mul0097	0.762549204	0.041176	0.085222	2.820262	Positive	132.1	86	1.27	131.0946	[M+H]+	L-Leucine	Amino acids
Mul0434	1.500853831	0.003003	0.011811	2.895807	Negative	117.02	100	1.55	118.0266	[M-H]-	Methylmalonic acid	Organic acids
Mul0140	20.50903097	0.005223	0.018313	2.89635	Positive	611.2	611.1	2.39	610.2	[M+H]+	Gallocatechin-gallocatechin	Catechin derivatives
Mul0084	-4.043480702	0.000267	0.002597	3.037874	Positive	136.1	136.1	1.21	135.0545	[M+H]+	Adenine	Nucleotide and its derivates
Mul0152	-2.950623206	0.000783	0.005129	3.050458	Positive	627.1	430	2.65	626.1	[M+H]+	8-C-hexosyl-hesperetin O-hexoside	Flavone C-glycosides
Mul0239	0.976742725	0.000837	0.005254	3.133984	Positive	465	302.9	4.04	464.096	[M+H]+	Quercetin 4'-O-glucoside (Spiraeoside)	Flavonol
Mul0444	-4.862336215	0.002409	0.010282	3.16846	Negative	299.1	137.2	1.96	300.1	[M-H]-	Salicylic acid O-glucoside	Phytohormones
Mul0110	1.778219279	0.00021	0.002245	3.231089	Positive	177	160.2	1.66	176.095	[M+H]+	serotonin	Tryptamine derivatives
Mul0315	-3.829398985	0.002019	0.009367	3.303221	Positive	494.3	184.6	7.71	493.3	[M+H]+	LysoPC 16:1 (2n isomer)	Lipids_Glycerophospholipids
Mul0217	20.82629653	2.61E-05	0.000815	3.324847	Positive	595.159	287	3.7	594.159	[M+H]+	Kaempferol 3-O-robinobioside (Biorobin)	Flavonol
Mul0043	-3.07098746	0.000203	0.002245	3.337993	Positive	116.1	70	0.79	115.0633	[M+H]+	L-Proline	Amino acids
Mul0233	20.93956136	3.21E-05	0.000929	3.458859	Positive	611.19	303	3.99	610.19	[M+H]+	Hesperetin 7-rutinoside (Hesperidin)	Flavanone
Mul0524	21.4321818	1.43E-05	0.000532	4.10533	Negative	593.159	285.1	3.77	594.1585	[M-H]-	Kaempferol 3-O-rutinoside (Nicotiflorin)	Flavonol
Mul0150	5.299932504	1.71E-06	0.00023	4.29001	Positive	595	287.9	2.6	595	Protonated	Cyanidin 3-O-rutinoside (Keracyanin)	Anthocyanins
Mul0531	1.837915752	3.77E-06	0.00032	4.568975	Negative	463.132	301.1	3.88	464.132	[M-H]-	Hesperetin 5-O-glucoside	Flavanone
Mul0516	1.897430266	5.15E-06	0.000383	4.635996	Negative	463	301.1	3.68	464.096	[M-H]-	Quercetin 3-O-glucoside (Isotrifoliin)	Flavonol
Mul0544	-6.951442914	0.000477	0.003778	4.702795	Negative	137	92.8	4.07	138.032	[M-H]-	Quercetin 3-O-glucoside (Isotrifoliin)	Phytohormones
Mul0145	21.87156126	1.85E-05	0.000611	4.778036	Positive	449.1	287.3	2.45	449.1	Protonated	Cyanidin 3-O-glucoside (Kuromanin)	Anthocyanins
Mul0219	21.88955193	9.86E-06	0.000513	4.811314	Positive	611.2	303.7	3.73	610.2	[M+H]+	Quercetin 7-O-rutinoside	Flavonol
Mul0201	21.91139069	7.71E-05	0.001275	4.835073	Positive	611.153	303	3.51	610.153	[M+H]+	Quercetin 3-O-rutinoside (Rutin)	Flavonol
Mul0413	1.402098444	0.02159	0.051711	5.435027	Negative	191	85	1.22	192.063	[M-H]-	Quinic acid	Quinate and its derivatives
Mul0460	23.69040866	0.001307	0.007255	8.860092	Negative	465.1	285.3	2.41	466.1	[M-H]-	Cyanidin O-syringic acid	Anthocyanins

Supplementary Table S3. Differentially accumulated metabolites between CS and BYW.

Index	log2_FC(case_mean/control_mean)	Pvalue	fdr	vip	Ion mode	Q1 (Da)	Q3 (Da)	Rt (min)	Molecular Weight (Da)	Ionization model	Compounds	Class
Mul0097	1.124666	0.00649	0.023086	4.318728	Positive	132.1	86	1.27	131.0946	[M+H]+	L-Leucine	Amino acids
Mul0092	1.117448	0.007765	0.02539	4.263655	Positive	132	85.9	1.23	131.0946	[M+H]+	L-Isoleucine	Amino acids
Mul0056	2.158148	0.000681	0.00586	3.360313	Positive	118.1	71.8	0.81	117.079	[M+H]+	L-Valine	Amino acids
Mul0064	2.08429	0.000845	0.006692	3.209017	Positive	118	88	0.85	117.079	[M+H]+	Dl-Norvaline	Amino acids
Mul0374	2.668951	0.000403	0.004583	1.989719	Negative	132	99.9	0.77	133.0375	[M-H]-	L-Aspartic acid	Amino acids
Mul0367	0.915354	0.041563	0.079897	1.96791	Negative	131.054	71.8	0.75	132.054	[M-H]-	L-Asparagine	Amino acids
Mul0078	3.112818	0.00051	0.005029	1.950255	Positive	150	72.8	1.18	149.051	[M+H]+	L-Methionine	Amino acids
Mul0375	-0.94945	0.042971	0.081138	1.91341	Negative	146.1	102.1	0.77	147.0532	[M-H]-	L-Glutamic acid	Amino acids
Mul0121	-0.87122	0.003195	0.015183	1.644755	Positive	166	120.1	1.96	165.079	[M+H]+	L-Phenylalanine	Amino acids
Mul0412	4.78118	4.59E-06	0.000545	1.027776	Negative	180.1	163	1.21	181.074	[M-H]-	L-(-)-Tyrosine	Amino acids
Mul0094	1.006717	0.006662	0.023361	3.049811	Positive	130	83.9	1.24	129.043	[M+H]+	5-oxoproline	Amino acid derivatives
Mul0443	-2.09068	0.022487	0.050981	2.817771	Negative	315.1	152.1	1.93	316.1	[M-H]-	2,5-dihydroxy benzoic acid O-hexside	Benzoic acid derivatives
Mul0511	-3.64115	0.009503	0.029145	1.75573	Negative	121.04	91.9	3.58	122.037	[M-H]-	4-Hydroxybenzaldehyde	Benzoic acid derivatives
Mul0048	-2.64714	0.01332	0.036128	1.175339	Positive	258.2	125.2	0.8	258.2	Protonated	sn-Glycero-3-phosphocholine	Cholines
Mul0471	1.691029	0.00176	0.011487	1.115058	Negative	315.1	153.2	2.55	316.1	[M-H]-	Protocatechuic acid O-glucoside	Catechin derivatives
Mul0200	-4.05889	0.00224	0.013176	1.764355	Positive	558.282	264.8	3.5	557.282	[M+H]+	N', N"-disinapoylspermidine	Phenolamides
Mul0001	-0.47896	0.047683	0.086882	1.519562	Positive	203	112	0.54	202	[M+H]+	Spermine	Phenolamides
Mul0120	1.597067	3.10E-05	0.002292	3.735124	Positive	167.1	121	1.95	166.1	[M+H]+	6-Methylmercaptopurine	Nucleotide and its derivates
Mul0084	-1.7892	0.000868	0.006781	2.904392	Positive	136.1	136.1	1.21	135.0545	[M+H]+	Adenine	Nucleotide and its derivates
Mul0117	-0.21882	0.003038	0.014654	1.638581	Positive	268.097	136.1	1.89	267.097	[M+H]+	Adenosine	Nucleotide and its derivates
Mul0051	-2.76812	0.041785	0.080066	1.616941	Positive	244.086	111.4	0.8	243.086	[M+H]+	Cytidine	Nucleotide and its derivates
Mul0404	2.915608	0.002827	0.014412	1.365623	Negative	565.1	323.3	1.18	566.1	[M-H]-	Uridine 5'-diphospho-D-glucose	Nucleotide and its derivates
Mul0432	-1.68863	0.015947	0.040483	1.199821	Negative	282.092	150.1	1.44	283.092	[M-H]-	Guanosine	Nucleotide and its derivates
Mul0155	1.919365	0.010725	0.031437	1.153718	Positive	298	135.1	2.73	297.09	[M+H]+	5'-Deoxy-5'-(methylthio)adenosine	Nucleotide and its derivates
Mul0113	-1.60372	0.003963	0.017438	1.015391	Positive	285.1	153.1	1.74	284.076	[M+H]+	Xanthosine	Nucleotide and its derivates
Mul0150	4.611689	0.001607	0.010723	3.691561	Positive	595	287.9	2.6	595	Protonated	Cyanidin 3-O-rutinoside (Keracyanin)	Anthocyanins
Mul0135	-5.82237	0.001149	0.00822	2.472055	Positive	465.1	303.1	2.3	465.1	Protonated	Delphinidin 3-O-glucoside (Mirtillin)	Anthocyanins
Mul0460	19.34018	0.003059	0.014654	2.170691	Negative	465.1	285.3	2.41	466.1	[M-H]-	Cyanidin O-syringic acid	Anthocyanins
Mul0148	18.41852	0.016506	0.04093	1.515909	Positive	433.1	271	2.58	433.1	Protonated	Pelargonidin 3-O-beta-D-glucoside（Callistephin chloride)	Anthocyanins
Mul0467	-6.42483	0.000665	0.005812	1.661539	Negative	771.1	609.5	2.47	772.1	[M-H]-	Luteolin O-hexosyl-O-hexosyl-O-hexoside	Flavone
Mul0530	2.980343	2.90E-06	0.000441	1.403585	Negative	503.1	341.3	3.88	504.1	[M-H]-	Chrysoeriol O-acetylhexoside	Flavone
Mul0515	-1.98656	0.004594	0.01903	1.142585	Negative	521.1	329.3	3.68	522.1	[M-H]-	Tricin O-saccharic acid	Flavone
Mul0221	-17.1767	0.003556	0.015933	1.020276	Positive	449	287.1	3.76	448	[M+H]+	Luteolin 7-O-glucoside	Flavone
Mul0201	21.36255	8.98E-05	0.002424	4.459434	Positive	611.153	303	3.51	610.153	[M+H]+	Quercetin 3-O-rutinoside (Rutin)	Flavonol
Mul0219	21.26202	2.97E-06	0.000441	4.306888	Positive	611.2	303.7	3.73	610.2	[M+H]+	Quercetin 7-O-rutinoside	Flavonol
Mul0529	2.331194	0.001979	0.012244	4.083021	Negative	505.1	301.2	3.87	506.1	[M-H]-	Quercetin O-acetylhexoside	Flavonol
Mul0516	0.935962	0.010116	0.030348	2.886952	Negative	463	301.1	3.68	464.096	[M-H]-	Quercetin 3-O-glucoside (Isotrifoliin)	Flavonol
Mul0239	0.612385	0.012174	0.034436	2.494541	Positive	465	302.9	4.04	464.096	[M+H]+	Quercetin 4'-O-glucoside (Spiraeoside)	Flavonol
Mul0524	18.60429	0.000462	0.004726	1.710056	Negative	593.159	285.1	3.77	594.1585	[M-H]-	Kaempferol 3-O-rutinoside (Nicotiflorin)	Flavonol
Mul0217	18.52576	0.003351	0.01543	1.648366	Positive	595.159	287	3.7	594.159	[M+H]+	Kaempferol 3-O-robinobioside (Biorobin)	Flavonol
Mul0162	2.099913	0.000595	0.005521	1.051297	Positive	713.1	713.1	2.83	712.1	[M+H]+	Quercetin 5-O-malonylhexosyl-hexoside	Flavonol
Mul0152	-0.92323	0.009675	0.02932	2.432712	Positive	627.1	430	2.65	626.1	[M+H]+	8-C-hexosyl-hesperetin O-hexoside	Flavone C-glycosides
Mul0531	0.966193	0.007041	0.024038	3.014463	Negative	463.132	301.1	3.88	464.132	[M-H]-	Hesperetin 5-O-glucoside	Flavanone
Mul0269	-3.02987	0.005179	0.020373	2.51429	Positive	273.1	137.1	5.3	272.069	[M+H]+	Butein	Flavanone
Mul0233	18.64135	0.000578	0.005452	1.714918	Positive	611.19	303	3.99	610.19	[M+H]+	Hesperetin 7-rutinoside (Hesperidin)	Flavanone
Mul0413	-1.46804	0.005017	0.019869	3.890246	Negative	191	85	1.22	192.063	[M-H]-	Quinic acid	Quinate and its derivatives
Mul0445	-18.8981	0.001273	0.008792	1.871537	Negative	355.1	181.2	1.97	356.1	[M-H]-	Homovanilloyl quinic acid	Quinate and its derivatives
Mul0508	0.609344	0.026779	0.057843	1.706714	Negative	337.1	190.9	3.44	338.1	[M-H]-	3-O-p-Coumaroyl quinic acid	Quinate and its derivatives
Mul0215	8.723201	0.006962	0.023905	1.106944	Positive	321.1	147.5	3.68	320.1	[M+H]+	5-O-p-Coumaroyl shikimic acid	Quinate and its derivatives
Mul0477	-1.49406	0.000295	0.004175	1.70672	Negative	341	179.1	2.62	342.132	[M-H]-	Coniferin	Hydroxycinnamoyl derivatives
Mul0050	-1.51908	0.010674	0.031437	2.070708	Positive	138	137.9	0.8	137.048	[M+H]+	Trigonelline	Alkaloids
Mul0074	2.505332	0.000351	0.004409	1.125143	Positive	86.1	56	1.17	85.1	[M+H]+	Piperidine	Alkaloids
Mul0398	2.328446	0.001041	0.007728	1.739124	Negative	259	96.9	0.99	260.03	[M-H]-	D-Glucose 6-phosphate	Carbohydrates
Mul0376	-1.62938	0.045979	0.084819	1.706329	Negative	179	70.9	0.77	180.063	[M-H]-	D(+)-Glucose	Carbohydrates
Mul0149	1.124494	0.037024	0.072105	1.285352	Positive	341	179.1	2.58	340.079	[M+H]+	Esculin (6,7-Dihydroxycoumarin-6-glucoside)	Coumarins
Mul0047	-1.16566	0.02939	0.062738	1.675012	Positive	256.2	124	0.8	255.2	[M+H]+	Nicotinate ribonucleoside	Nicotinic acid derivatives
Mul0139	2.699733	0.001198	0.00847	1.064639	Positive	206.1	147.5	2.39	205.1	[M+H]+	Methoxyindoleacetic acid	Indole derivatives
Mul0418	2.850806	6.09E-05	0.002413	4.913999	Negative	133	70.8	1.23	134.022	[M-H]-	L(-)-Malic acid	Organic acids
Mul0427	1.976722	0.000151	0.002849	4.212903	Negative	117.03	99.9	1.31	118.027	[M-H]-	Succinic acid	Organic acids
Mul0434	1.452999	0.000617	0.005637	3.189504	Negative	117.02	100	1.55	118.0266	[M-H]-	Methylmalonic acid	Organic acids
Mul0025	2.64545	0.000146	0.002849	2.087319	Positive	132.1	86.2	0.75	131.1	[M+H]+	5-Aminolevulinate	Organic acids
Mul0480	1.456675	0.000208	0.003195	2.061985	Negative	175.1	131.3	2.73	176.1	[M-H]-	2-Isopropylmalate	Organic acids

Mul0450	0.757568	0.002087	0.012647	1.157112	Negative	131.042	86.9	2.15	132.0423	[M-H]-	2-Methylsuccinic acid	Organic acids
Mul0067	2.868755	0.000163	0.002942	1.080412	Positive	132.095	76.9	0.93	131.0946	[M+H]+	6-Aminocaproic acid	Organic acids
Mul0490	-2.75018	0.021653	0.050046	1.016282	Negative	137.03	92.5	2.98	138.032	[M-H]-	4-Hydroxybenzoic acid	Organic acids
Mul0318	3.636946	0.00021	0.003195	4.270911	Positive	520	125	8.08	519	[M+H]+	LysoPC 18:2 (2n isomer)	Lipids_Glycerophospholipids
Mul0315	-3.1916	0.001914	0.011966	3.59373	Positive	494.3	184.6	7.71	493.3	[M+H]+	LysoPC 16:1 (2n isomer)	Lipids_Glycerophospholipids
Mul0588	1.65101	0.021884	0.050382	3.436401	Negative	452	255.3	8.57	453	[M-H]-	LysoPE 16:0	Lipids_Glycerophospholipids
Mul0335	0.632806	0.018268	0.043405	3.032388	Positive	496.3	478.3	8.65	495.3	[M+H]+	LysoPC 16:0	Lipids_Glycerophospholipids
Mul0336	0.810786	0.016636	0.041004	3.004758	Positive	522.4	184.5	8.76	521.4	[M+H]+	LysoPC 18:1 (2n isomer)	Lipids_Glycerophospholipids
Mul0326	4.402583	0.002378	0.013324	2.416415	Positive	520.3	502.3	8.27	519.3	[M+H]+	LysoPC 18:2	Lipids_Glycerophospholipids
Mul0331	1.185721	0.032205	0.066888	1.739536	Positive	496.33	478.3	8.49	495.33	[M+H]+	LysoPC 16:0 (2n isomer)	Lipids_Glycerophospholipids
Mul0313	6.890017	0.015719	0.040483	1.430511	Positive	468.1	450.5	7.57	467.1	[M+H]+	LysoPC 14:0	Lipids_Glycerophospholipids
Mul0334	1.1438	0.017493	0.042068	1.32586	Positive	518.3	459.6	8.65	517.3	[M+H]+	LysoPC 18:3	Lipids_Glycerophospholipids
Mul0323	5.451924	0.001856	0.011853	1.149302	Positive	478.6	337.9	8.2	477.6	[M+H]+	LysoPE 18:2 (2n isomer)	Lipids_Glycerophospholipids
Mul0587	2.55955	0.013398	0.036175	1.144428	Negative	452.3	255.3	8.4	453.3	[M-H]-	LysoPE 16:0 (2n isomer)	Lipids_Glycerophospholipids
Mul0589	0.982282	0.014343	0.037866	1.045934	Negative	478.1	281.3	8.69	479.1	[M-H]-	LysoPE 18:1 (2n isomer)	Lipids_Glycerophospholipids
Mul0353	-1.79664	0.000416	0.004583	2.216916	Positive	353.2	261.8	9.91	352.2	[M+H]+	MAG (18:3) isomer1	Lipids_Glycerolipids
Mul0338	1.505818	0.011019	0.031928	2.215065	Positive	279.3	201.5	8.81	278.3	[M+H]+	Punicic acid	Lipids_Fatty acids
Mul0345	2.452674	0.014471	0.038034	2.01394	Positive	295.2	179.5	9.17	294.2	[M+H]+	9-Hydroxy-(10E,12Z,15Z)-octadecatrienoic acid	Lipids_Fatty acids
Mul0590	1.49988	0.012081	0.034436	1.855271	Negative	295	183.3	8.82	296	[M-H]-	9,10-EODE	Lipids_Fatty acids
Mul0544	-6.33719	0.000485	0.004886	5.19421	Negative	137	92.8	4.07	138.032	[M-H]-	Salicylic acid (SA)	Phytohormones
Mul0444	-5.59902	0.002288	0.013199	3.553304	Negative	299.1	137.2	1.96	300.1	[M-H]-	Salicylic acid O-glucoside	Phytohormones

Supplementary Table S4. Differentially accumulated metabolites between HG2 and BYW.

Index	log2_FC(case_mean/control_mean)	Pvalue	fdr	vip	Ion mode	Q1 (Da)	Q3 (Da)	Rt (min)	Molecular Weight (Da)	Ionization model	Compounds	Class
Mul0056	1.561504	0.000574	0.004732	2.898584	Positive	118.1	71.8	0.81	117.079	[M+H]+	L-Valine	Amino acids
Mul0064	1.575115	0.000649	0.005004	2.802461	Positive	118	88	0.85	117.079	[M+H]+	Dl-Norvaline	Amino acids
Mul0092	0.4197	0.002011	0.009406	2.780579	Positive	132	85.9	1.23	131.0946	[M+H]+	L-Isoleucine	Amino acids
Mul0097	0.362117	0.001691	0.008439	2.636583	Positive	132.1	86	1.27	131.0946	[M+H]+	L-Leucine	Amino acids
Mul0367	2.026472	0.000274	0.00362	2.568908	Negative	131.054	71.8	0.75	132.054	[M-H]-	L-Asparagine	Amino acids
Mul0374	1.561211	0.001238	0.00699	1.628287	Negative	132	99.9	0.77	133.0375	[M-H]-	L-Aspartic acid	Amino acids
Mul0094	2.819428	8.37E-05	0.002106	3.840097	Positive	130	83.9	1.24	129.043	[M+H]+	5-oxoproline	Amino acid derivatives
Mul0077	-6.71187	0.000565	0.004728	1.444157	Positive	308	162	1.18	307.084	[M+H]+	Glutathione reduced form	Amino acid derivatives
Mul0443	-1.06751	0.029846	0.065576	1.484179	Negative	315.1	152.1	1.93	316.1	[M-H]-	2,5-dihydroxy benzoic acid O-hexside	Benzoic acid derivatives
Mul0039	1.549138	0.000977	0.00652	2.304508	Positive	104.1	60.2	0.78	103.1	[M+H]+	Choline	Cholines
Mul0140	-7.19485	0.005355	0.018698	2.99802	Positive	611.2	611.1	2.39	610.2	[M+H]+	Gallocatechin-gallocatechin	Catechin derivatives
Mul0001	-0.29401	0.008255	0.025147	1.132749	Positive	203	112	0.54	202	[M+H]+	Spermine	Phenolamides
Mul0120	0.720114	0.000236	0.003334	2.647598	Positive	167.1	121	1.95	166.1	[M+H]+	6-Methylmercaptopurine	Nucleotide and its derivates
Mul0084	2.254285	0.000555	0.004709	1.54097	Positive	136.1	136.1	1.21	135.0545	[M+H]+	Adenine	Nucleotide and its derivates
Mul0460	-4.35023	0.001593	0.008228	8.948604	Negative	465.1	285.3	2.41	466.1	[M-H]-	Cyanidin O-syringic acid	Anthocyanins
Mul0145	-21.8716	1.85E-05	0.001761	4.929835	Positive	449.1	287.3	2.45	449.1	Protonated	Cyanidin 3-O-glucoside (Kuromanin)	Anthocyanins
Mul0150	-0.68824	0.007661	0.023658	2.668597	Positive	595	287.9	2.6	595	Protonated	Cyanidin 3-O-rutinoside (Keracyanin)	Anthocyanins
Mul0173	-4.97923	0.000233	0.003334	2.266757	Positive	463.123	301.4	3	463.123	Protonated	Peonidin O-hexoside	Anthocyanins
Mul0148	18.41852	0.016506	0.041722	1.407497	Positive	433.1	271	2.58	433.1	Protonated	Pelargonidin 3-O-beta-D-glucoside（Callistephin chloride)	Anthocyanins
Mul0530	7.709239	4.77E-07	0.000284	1.397094	Negative	503.1	341.3	3.88	504.1	[M-H]-	Chrysoeriol O-acetylhexoside	Flavone
Mul0515	-1.91744	0.002114	0.009683	1.046573	Negative	521.1	329.3	3.68	522.1	[M-H]-	Tricin O-saccharic acid	Flavone
Mul0529	7.511568	0.000805	0.005624	4.234331	Negative	505.1	301.2	3.87	506.1	[M-H]-	Quercetin O-acetylhexoside	Flavonol
Mul0524	-2.82789	3.26E-05	0.001761	3.926413	Negative	593.159	285.1	3.77	594.1585	[M-H]-	Kaempferol 3-O-rutinoside (Nicotiflorin)	Flavonol
Mul0516	-0.96147	0.000718	0.005199	3.870477	Negative	463	301.1	3.68	464.096	[M-H]-	Quercetin 3-O-glucoside (Isotrifoliin)	Flavonol
Mul0217	-2.30053	0.000143	0.00266	3.052021	Positive	595.159	287	3.7	594.159	[M+H]+	Kaempferol 3-O-robinobioside (Biorobin)	Flavonol
Mul0219	-0.62753	0.000894	0.006177	2.912885	Positive	611.2	303.7	3.73	610.2	[M+H]+	Quercetin 7-O-rutinoside	Flavonol
Mul0201	-0.54884	0.012824	0.034783	2.669649	Positive	611.153	303	3.51	610.153	[M+H]+	Quercetin 3-O-rutinoside (Rutin)	Flavonol
Mul0239	-0.36436	0.005761	0.01901	2.146034	Positive	465	302.9	4.04	464.096	[M+H]+	Quercetin 4'-O-glucoside (Spiraeoside)	Flavonol
Mul0162	17.62822	0.000207	0.003185	1.128998	Positive	713.1	713.1	2.83	712.1	[M+H]+	Quercetin 5-O-malonylhexosyl-hexoside	Flavonol
Mul0577	2.838234	0.011357	0.031921	1.049348	Negative	329.1	314.3	5.76	330.1	[M-H]-	Di-O-methylquercetin	Flavonol
Mul0152	2.027396	0.000798	0.005624	2.126526	Positive	627.1	430	2.65	626.1	[M+H]+	8-C-hexosyl-hesperetin O-hexoside	Flavone C-glycosides
Mul0501	-1.87475	0.003725	0.014184	1.666746	Negative	449.1	329.3	3.21	450.1	[M-H]-	Eriodictyol C-hexoside	Flavone C-glycosides
Mul0212	-1.88469	0.000486	0.004374	1.503384	Positive	611.2	299.8	3.67	610.2	[M+H]+	Luteolin 8-C-hexosyl-O-hexoside	Flavone C-glycosides
Mul0531	-0.87172	0.001026	0.006554	3.700161	Negative	463.132	301.1	3.88	464.132	[M-H]-	Hesperetin 5-O-glucoside	Flavanone
Mul0233	-2.29821	0.000109	0.002407	3.18854	Positive	611.19	303	3.99	610.19	[M+H]+	Hesperetin 7-rutinoside (Hesperidin)	Flavanone
Mul0260	-2.97923	0.012971	0.035021	2.221447	Positive	289.063	153.1	4.85	288.063	[M+H]+	Eriodictyol	Flavanone
Mul0546	-3.24372	0.000293	0.003659	1.964797	Negative	609.2	301.3	4.09	610.19	[M-H]-	Hesperetin 7-O-neohesperidoside (Neohesperidin)	Flavanone
Mul0413	-2.87014	0.006293	0.020097	6.796869	Negative	191	85	1.22	192.063	[M-H]-	Quinic acid	Quinate and its derivatives
Mul0478	0.829689	0.042367	0.086482	2.062756	Negative	353.095	191.1	2.69	354.0951	[M-H]-	Chlorogenic acid (3-O-Caffeoylquinic acid)	Quinate and its derivatives
Mul0456	0.239501	0.02939	0.065138	1.858831	Negative	353.1	191.1	2.21	354.095	[M-H]-	1-O-Caffeoyl quinic acid	Quinate and its derivatives
Mul0215	9.119339	0.006949	0.021839	1.022803	Positive	321.1	147.5	3.68	320.1	[M+H]+	5-O-p-Coumaroyl shikimic acid	Quinate and its derivatives
Mul0477	4.011197	0.001172	0.006922	1.133373	Negative	341	179.1	2.62	342.132	[M-H]-	Coniferin	Hydroxycinnamoyl derivatives
Mul0110	-1.46409	0.001764	0.008618	3.138517	Positive	177	160.2	1.66	176.095	[M+H]+	serotonin	Tryptamine derivatives
Mul0050	-1.15915	0.000406	0.00409	1.631196	Positive	138	137.9	0.8	137.048	[M+H]+	Trigonelline	Alkaloids
Mul0379	0.73187	0.008483	0.025709	1.425679	Negative	341	58.8	0.78	342.1162	[M-H]-	D-(+)-Sucrose	Carbohydrates
Mul0376	-0.89414	0.018312	0.045322	1.065684	Negative	179	70.9	0.77	180.063	[M-H]-	D(+)-Glucose	Carbohydrates
Mul0149	3.562936	0.001687	0.008439	1.70681	Positive	341	179.1	2.58	340.079	[M+H]+	Esculin (6,7-Dihydroxycoumarin-6-glucoside)	Coumarins
Mul0047	-0.65315	0.000613	0.004921	1.139391	Positive	256.2	124	0.8	255.2	[M+H]+	Nicotinate ribonucleoside	Nicotinic acid derivatives
Mul0418	21.86038	3.24E-05	0.001761	4.911363	Negative	133	70.8	1.23	134.022	[M-H]-	L(-)-Malic acid	Organic acids
Mul0427	4.25887	0.000202	0.003185	4.391659	Negative	117.03	99.9	1.31	118.027	[M-H]-	Succinic acid	Organic acids
Mul0386	-0.45621	0.016668	0.041953	2.398184	Negative	191	131	0.81	192.027	[M-H]-	Citric acid	Organic acids
Mul0480	1.138898	8.06E-05	0.002106	1.781241	Negative	175.1	131.3	2.73	176.1	[M-H]-	2-Isopropylmalate	Organic acids
Mul0025	1.526189	0.000131	0.00266	1.71567	Positive	132.1	86.2	0.75	131.1	[M+H]+	5-Aminolevulinate	Organic acids
Mul0450	0.767389	0.000954	0.006517	1.08689	Negative	131.042	86.9	2.15	132.0423	[M-H]-	2-Methylsuccinic acid	Organic acids
Mul0318	1.525641	0.027269	0.061355	3.112995	Positive	520	125	8.08	519	[M+H]+	LysoPC 18:2 (2n isomer)	Lipids_Glycerophospholipids
Mul0326	2.087306	0.005734	0.01901	1.970729	Positive	520.3	502.3	8.27	519.3	[M+H]+	LysoPC 18:2	Lipids_Glycerophospholipids
Mul0331	2.253607	0.010679	0.030643	1.946593	Positive	496.33	478.3	8.49	495.33	[M+H]+	LysoPC 16:0 (2n isomer)	Lipids_Glycerophospholipids
Mul0587	2.583099	0.013124	0.035275	1.046758	Negative	452.3	255.3	8.4	453.3	[M-H]-	LysoPE 16:0 (2n isomer)	Lipids_Glycerophospholipids
Mul0356	-1.41914	0.010944	0.031252	2.325112	Positive	355.1	337.9	10.36	354.1	[M+H]+	MAG (18:2)	Lipids_Glycerolipids
Mul0287	-4.43296	0.044453	0.088905	2.248124	Positive	318.3	300.9	6.63	317.3	[M+H]+	4-Hydroxysphinganine	Lipids_Fatty acids
Mul0338	0.79865	0.001247	0.00699	1.730812	Positive	279.3	201.5	8.81	278.3	[M+H]+	Punicic acid	Lipids_Fatty acids
Mul0345	1.603234	0.022003	0.052489	1.687546	Positive	295.2	179.5	9.17	294.2	[M+H]+	9-Hydroxy-(10E,12Z,15Z)-octadecatrienoic acid	Lipids_Fatty acids

Mul0590	1.033979	0.000513	0.004545	1.60706	Negative	295	183.3	8.82	296	[M-H]-	9,10-EODE	Lipids_Fatty acids
Mul0566	1.04875	0.020197	0.049371	1.005155	Negative	263	153.2	4.93	264.136	[M-H]-	(+)-cis,trans-Abscisic acid (ABA)	Phytohormones

Supplementary Table S5. Statistics of the sequencing reads for mulberry fruits.

Sequencing details	CS-1	CS-2	CS-3	HG2-1	HG2-2	HG2-3	BYW-1	BYW-2	BYW-3
Raw reads	46837546	45809226	45936772	45296040	48417824	50684876	46650630	46498720	57557936
Clean reads	45704960	44576292	44390454	44081086	46640874	49137728	44845108	45172650	56284532
Clean bases	6.27G	6.10G	6.06G	6.03G	6.36G	6.72G	6.11G	6.19G	7.72G
Q20 (%)	98.29	98.19	97.99	98.23	97.94	98.10	97.86	98.13	98.22
Q30 (%)	95.01	94.75	94.31	94.87	94.19	94.55	93.97	94.61	94.64
GC (%)	46.77	47.27	47.28	46.97	46.95	46.99	48.32	47.41	47.05

Supplementary Table S6. Structural genes involved in flavonoid metabolic pathway in mulberry fruits.

**Function**	**Gene**	**Enzyme**	**KO id (EC no.)**	**No. All[a]**	**No. Changed[b]**
Phenylpropanold biosynthesis	*PAL*	Phenylalanine ammonia-lyase	k10775 (4.3.1.24)	6	6
	C4H	Trans-cinnamate 4-monooxygenase	k00487 (1.14.13.11)	4	3
	4CL	4-coumarate--CoA ligase	k01904 (6.2.1.12)	7	6
Anthocyanin biosynthesis	*CHS*	Chalcone synthase	K00660 (2.3.1.74)	7	6
	CHI	Chalcone isomerase	K01859 (5.5.1.6)	2	2
	F3H	Flavanone 3-hydroxylase	K00475 (1.14.11.9)	2	2
	CYP75B1	***Flavonoid 3'-hydroxylase***	K05280 (1.14.13.21)	1	1
	CYP75B2	***Flavonoid 3'-hydroxylase***	K05280 (1.14.13.21)	2	2
	DFR	Dihydroflavonol 4-reductase	K13082 (1.1.1.219)	1	1
	ANS	Anthocyanidin synthase	K05277 (1.14.11.19)	1	1
	UFGT	Anthocyanidin 3-O-glucosyltransfersae	K12930 (2.4.1.115)	1	1
	UGAT	Cyanidin-3-O-glucoside 2"-O-glucuronosyltransferase	k12937 (2.4.1.254)	1	1
	UGT75C1	Anthocyanidin 3-O-glucoside 5-O-glucosyltransferase	K12338 (2.4.1. 298)	1	1
Flavone and flavonol biosynthesis	*FNS*	Flavone synthase	K13077 (1.14.11.22)	1	1
	FLS	Flavonol synthase	K05278 (1.14.11.23)	2	1
Flavanone biosynthesis	*LAR*	Leucoanthocyanidin reductase	K13081 (1.17.1.3)	1	1
	ANR	Anthocyanidin reductase	K08695 (1.3.1.77)	1	1

[a] No. All, the total number of uni-transcripts analysed.

[b] No.Changed, the number of uni-transcripts with expression significantly changed in friuts of Mulberry among the three cultivars.

Supplementary Table S7. Plant materials used in this study.

Abbreviation	Cultivars	Collection date	Collection place	Fruit color
CS	*Morus notabilis* C.K. Schneid	August	Ya'an City, Sichuan Province, China	Yellow-green color
HG2	*Morus alba* L. cv. Hongguo2	May	Beibei District, Chongqing City, China	Purple color
D10	*Morus alba* L. cv. Da10	May	Beibei District, Chongqing City, China	Purple color
BYW	*Morus alba* L. cv. Baiyuwang	June	Beibei District, Chongqing City, China	Oyster-white color
ZZB	*Morus alba* L. cv. Zhenzhubai	June	Beibei District, Chongqing City, China	White color
LJ109	*Morus alba* L. cv. Lunjiao109 (Monoecious)	May	Beibei District, Chongqing City, China	Red color
HGDBZZ	*Morus alba* L. cv. Hanguodabaizhenzhu	June	Beibei District, Chongqing City, China	Oyster-white color
F1-W	F1 population derived from LJ109 (male parent) and ZZB (female parent)	June	Beibei District, Chongqing City, China	White color
F1-Male	F1 population derived from LJ109 (male parent) and ZZB (female parent)	May	Beibei District, Chongqing City, China	No fruit, male tree
F2-P	F2 population derived from F1-W and F1-Male	May	Beibei District, Chongqing City, China	Red alternating with white
F2-R	F2 population derived from F1-W and F1-Male	May	Beibei District, Chongqing City, China	Red color
F2-W	F2 population derived from F1-W and F1-Male	June	Beibei District, Chongqing City, China	White color

Supplementary Table S8. Primers used in this work.

Primer Name	Forward primer	Reverse primer
**Primers used for cloning candidate mulberry genes.**		
MYBA	5’-ATGCAAACGGAGGGCTGTTC-3’	5’-CTACTGCATTTCAGCATTTCCT-3’
TT2L1	5’-ATGGGAAGAAACCCTTGTTGC-3’	5’-TTAATCAGTCAACCAGTTTTCTTCATCG-3’
TT2L2	5’-ATGGGAAGAAAACCTTGTTGTTCAA-3’	5’-TTAAACTTGAAGCCACCTATCGAAG-3’
TT2L3	5’-GATCTGGAGAGGGAGAGTGA-3’	5’-TTATAGGGCTAGAGTAAAGTCAATA-3’
bHLH3	5’-ATGTCTTGTCTTGATTTGACCTTCT-3’	5’-TCAATTATCGCTGTGGGGTAT-3’
GL3	5’-ATGGCTACTGGGCTCCAAAAC-3’	5’-TCAACTGTTCCAAGCAATTCTCTG-3’
TTG1	5’-AATTTTCCGTTATTCTGGCG-3’	5’-AGCAAGAAAAAATAAAAATCAAAAG-3’
MYBF	5’-ATGGGAAGAGCTCCGTGCT-3’	5’-TCAGGAGAGAAGCCAAGCGA-3’
MYB4	5’-ATGAACAAAGGAGCTTGGTCAA-3’	5’-TCATGTGAAGAGATTTTGAAGCGT-3’
CYP75B1	5’-ATGGCCTCTATCACCACCAT-3’	5’-TCAATATAAGTGTTGGTCTAGTCT-3’
CYP75B2	5’-ATGCCCTCTATCTTCACCATTC-3’	5’-CTAAATTTTTGCTTCATATACATG-3’
L484_012842	5’-AGTAATTCCATTATGGACTCCTCG-3’	5’-ATTTACACAAACAATCACCACACAT-3’
**Primers for cell-feeding assays in yeast**		
CYP75B-pYES2	5’-accgagctcggatccATGGCCTCTATCACCACCAT-3’	5’-agatgcatgctcgagTCAATATAAGTGTTGGTCTAGTCT-3’
CYP75B2-pYES2	5’-accgagctcggatccATGCCCTCTATCTTCACCATTC-3’	5’-agatgcatgctcgagCTAAATTTTTGCTTCATATACATG-3’
L484_012842-pYES2	5’-accgagctcggatccATGGACTCCTCGTCGTCGT-3’	5’-agatgcatgctcgagTCACCACACATCACGTAGATACCT-3’
**Primers for dual-luciferase reporter and transient color assays**		
CHS2P-LUC	5’-ggtatcgataagcttGTCTGAAAACAGGAGTCGGA-3’	5’-agaactagtggatccTTTCAAAGAATTCACCGAGGATCT-3’
CHIP-LUC	5’-ggtatcgataagcttGTAGCCTCATTTCGTCCTTT-3’	5’-agaactagtggatccTTGGGTTCGGTTTTTGTTGA-3’
FNSIP-LUC	5’-ggtatcgataagcttTACGCAATTTTATACAAATCGAC-3’	5’-agaactagtggatccTTATTACTTGTCTGTAAGATGTCAAAAT-3’
CYP75B1P-LUC	5’-ggtatcgataagcttAAATTTCTTGCACGCAATTA-3’	5’-agaactagtggatccAAGATAAAAGAGTTTCTGGAAGC-3’
CYP75B2P-LUC	5’-ggtatcgataagcttTTTGGGCAAAGCAGGGACTA-3’	5’-agaactagtggatccTGTGCTGTCTATGTTTTGTTTTTTT-3’
FLS1P-LUC	5’-ggtatcgataagcttGAATCAGCGAACTAAACGGG-3’	5’-agaactagtggatccGTTGTCTACTTAGCTAGCTAGATATCTT-3’
FLS3P-LUC	5’-ggtatcgataagcttTTTGAAGAACGCATAAGGTC-3’	5’-agaactagtggatccGGCTACCAATAAGTTAAAGCTAG-3’
DFRP-LUC	5’-ggtatcgataagcttTAGCTAACGGTACGTTTGGTAGTGA-3’	5’-agaactagtggatccATTGCTGCTAATCTTTTGAAAGACA-3’
LARP-LUC	5’-ggtatcgataagcttTTTAGTCGACGTTGTGAGAGTATG-3’	5’-agaactagtggatccGTTTGCTCTTTTTTTTTTTTTTTTT-3’
ANRP-LUC	5’-ggtatcgataagcttCATATTTGACGTTAAGCCTCTACG-3’	5’-agaactagtggatccGGCTATTGGATTCTTATTTTCTTTT-3’
ANSP-LUC	5’-ggtatcgataagcttTTGAAACAAGGAGGGTTAAACTCA-3’	5’-agaactagtggatccATTAGCCTAATTTGTACCACTACTTTTC-3’
UFGTP-LUC	5’-ggtatcgataagcttATGTTTGTCTCATTCTCGTA-3’	5’-agaactagtggatccGGTAAGTTTGTTATGGAGGT-3’
MYB4P-LUC	5’-ggtatcgataagcttGCTTGAATTACTTTTGTGCGTGTG-3’	5’-agaactagtggatccTTCTCGCTTCTCACAGCAAGG-3’
MYBA-SK	5’-agaactagtggatccATGCAAACGGAGGGCTGTTC-3’	5’-taccgaattggtaccCTACTGCATTTCAGCATTTCCT-3’
TT2L1-SK	5’-agaactagtggatccATGGGAAGAAACCCTTGTTGC-3’	5’-taccgaattggtaccTTAATCAGTCAACCAGTTTTCTTCATCG-3’
TT2L2-SK	5’-agaactagtggatccATGGGAAGAAAACCTTGTTGTTCAA-3’	5’-taccgaattggtaccTTAAACTTGAAGCCACCTATCGAAG-3’
TT2L3-SK	5’-agaactagtggatccATGGGAGAGAGACGTCCTCG-3’	5’-taccgaattggtaccTCAATATTGTTGATCAGGAAAGGT-3’
bHLH3-SK	5’-agaactagtggatccATGTCTTGTCTTGATTTGACCTTCT-3’	5’-taccgaattggtaccTCAATTATCGCTGTGGGGTAT-3’
GL3-SK	5’-agaactagtggatccATGGCTACTGGGCTCCAAAAC-3’	5’-taccgaattggtaccTCAACTGTTCCAAGCAATTCTCTG-3’
TTG1-SK	5’-agaactagtggatccATGGAGAACACGACACAAGAATC-3’	5’-taccgaattggtaccTCAAACTTTCAAAAGCTGCATTT-3’
MYBF-SK	5’-agaactagtggatccATGGGAAGAGCTCCGTGCT-3’	5’-taccgaattggtaccTCAGGAGAGAAGCCAAGCGA-3’
MYB4-SK	5’-agaactagtggatccATGAACAAAGGAGCTTGGTCAA-3’	5’-taccgaattggtaccTCATGTGAAGAGATTTTGAAGCGT-3’
**Primers for Y1H, Y2H and Y3H assays.**		
MYBA-AD	5’-gattacgctcatatgCAAACGGAGGGCTGTTC-3’	5’-catctgcagctcgagCTACTGCATTTCAGCATTTCCT-3’
TT2L1-AD	5’-gattacgctcatatgGGAAGAAACCCTTGTTGC-3’	5’-catctgcagctcgagTTAATCAGTCAACCAGTTTTCTTCATCG-3’
TT2L2-AD	5’-gattacgctcatatgGGAAGAAAACCTTGTTGTTCAA-3’	5’-catctgcagctcgagTTAAACTTGAAGCCACCTATCGAAG-3’
TT2L3-AD	5’-gattacgctcatatgGGAGAGAGACGTCCTCG-3’	5’-tcatctgcagctcgagTCAATATTGTTGATCAGGAAAGGT-3’
TTG1-AD	5’-gattacgctcatatgGAGAACACGACACAAGAATC-3’	5’-catctgcagctcgagTCAAACTTTCAAAAGCTGCATTT-3’
MYBF-AD	5’-gattacgctcatatgGGAAGAGCTCCGTGCT-3’	5’-catctgcagctcgagTCAGGAGAGAAGCCAAGCGA-3’
MYB4-AD	5’-gattacgctcatatgAACAAAGGAGCTTGGTCAA-3’	5’-catctgcagctcgagTCATGTGAAGAGATTTTGAAGCGT-3’
MYBA-BK	5’-gaggacctgcatatgCAAACGGAGGGCTGTTC-3’	5’-caggtcgacggatccCTACTGCATTTCAGCATTTCCT-3’
TT2L1-BK	5’-gaggacctgcatatgGGAAGAAACCCTTGTTGC-3’	5’-caggtcgacggatccTTAATCAGTCAACCAGTTTTCTTCATCG-3’
TT2L2-BK	5’-gaggacctgcatatgGGAAGAAAACCTTGTTGTTCAA-3’	5’-caggtcgacggatccTTAAACTTGAAGCCACCTATCGAAG-3’
TT2L3-BK	5’-gaggacctgcatatgGGAGAGAGACGTCCTCG-3’	5’-caggtcgacggatccTCAATATTGTTGATCAGGAAAGGT-3’
bHLH3-BK	5’-gaggacctgcatatgTCTTGTCTTGATTTGACCTTCT-3’	5’-caggtcgacggatccTCAATTATCGCTGTGGGGTAT-3’
GL3-BK	5’-gaggacctgcatatgGCTACTGGGCTCCAAAAC-3’	5’-caggtcgacggatccTCAACTGTTCCAAGCAATTCTCTG-3’
TTG1-BK	5’-gaggacctgcatatgGAGAACACGACACAAGAATC-3’	5’-caggtcgacggatccTCAAACTTTCAAAAGCTGCATTT-3’
MYBF-BK	5’-gaggacctgcatatgGGAAGAGCTCCGTGCT-3’	5’-caggtcgacggatccTCAGGAGAGAAGCCAAGCGA-3’
MYB4-BK	5’-gaggacctgcatatgAACAAAGGAGCTTGGTCAA-3’	5’-caggtcgacggatccTCATGTGAAGAGATTTTGAAGCGT-3’
ANSP-pBait	5’-*GGGGTACC*TTGAAACAAGGAGGGTTAAACTCA-3’	5’-*CCGCTCGAG*ATTAGCCTAATTTGTACCACTACTTTTC-3’
LARP-pBait	5’-*GGGGTACC*TTTAGTCGACGTTGTGAGAGTATG-3’	5’-*CCGCTCGAG*GTTTGCTCTTTTTTTTTTTTTTTTT-3’
FLS1P-pBait	5’-*GGGGTACC*GAATCAGCGAACTAAACGGG-3’	5’-*CCGCTCGAG*GTTGTCTACTTAGCTAGCTAGATATCTT-3’
TTG1-pBridge	5’-gtatcgccggaattcATGGAGAACACGACACAAGAATC-3’	5’-caggtcgacggatccTCAAACTTTCAAAAGCTGCATTT-3’
bHLH3-pBridge	5’-aaaggtggcggccgcaATGTCTTGTCTTGATTTGACCTTCT-3’	5’-tcagcccgaagatctTCAATTATCGCTGTGGGGTAT-3’
GL3-pBridge	5’-aaaggtggcggccgcaATGGCTACTGGGCTCCAAAAC-3’	5’-tcagcccgaagatctTCAACTGTTCCAAGCAATTCTCTG-3’
**Primers for BiFC assays**		

MYBA-BiFC	5'-gccactagtggatccATGCAAACGGAGGGCTGTTC-3'	5'-cccgggagcggtaccCTGCATTTCAGCATTTCCT-3'
TT2L1-BiFC	5'-gccactagtggatccATGGGAAGAAACCCTTGTTGC-3'	5'-cccgggagcggtaccATCAGTCAACCAGTTTTCTTCATCG-3'
TT2L2-BiFC	5'-gccactagtggatccATGGGAAGAAAACCTTGTTGTTCAA-3'	5'-cccgggagcggtaccAACTTGAAGCCACCTATCGAAG-3'
TT2L3-BiFC	5'-gccactagtggatccATGGGAGAGAGACGTCCTCG-3'	5'-cccgggagcggtaccATATTGTTGATCAGGAAAGGT-3'
TTG1-BiFC	5'-gccactagtggatccATGGAGAACACGACACAAGAATC-3'	5'-cccgggagcggtaccAACTTTCAAAAGCTGCATTT-3'
MYB4-BiFC	5'-gccactagtggatccATGAACAAAGGAGCTTGGTCAA-3'	5'-cccgggagcggtaccTCATGTGAAGAGATTTTGAAGCGT-3'
bHLH3-BiFC	5'-gccactagtggatccATGTCTTGTCTTGATTTGACCTTCT-3'	5'-cccgggagcggtaccATTATCGCTGTGGGGTAT-3'
GL3-BiFC	5'-gccactagtggatccATGGCTACTGGGCTCCAAAAC-3'	5'-cccgggagcggtaccACTGTTCCAAGCAATTCTCTG-3'
**Primers for split luciferase complementation assays**		
MYBA-Nluc	5'-gacgagctcggtaccATGCAAACGGAGGGCTGTTC-3'	5'-ccatttgttggatccCTGCATTTCAGCATTTCCT-3'
TT2L1-Nluc	5'-gacgagctcggtaccATGGGAAGAAACCCTTGTTGC-3'	5'-ccatttgttggatccATCAGTCAACCAGTTTTCTTCATCG-3'
TT2L2-Nluc	5'-gacgagctcggtaccATGGGAAGAAAACCTTGTTGTTCAA-3'	5'-ccatttgttggatccAACTTGAAGCCACCTATCGAAG-3'
TT2L3-Nluc	5'-gacgagctcggtaccATGGGAGAGAGACGTCCTCG-3'	5'-ccatttgttggatccATATTGTTGATCAGGAAAGGT-3'
TTG1-Nluc	5'-gacgagctcggtaccATGGAGAACACGACACAAGAATC-3'	5'-ccatttgttggatccAACTTTCAAAAGCTGCATTT-3'
MYB4-Nluc	5'-gacgagctcggtaccATGAACAAAGGAGCTTGGTCAA-3'	5'-ccatttgttggatccTGTGAAGAGATTTTGAAGCGT-3'
bHLH3-Cluc	5'-tcccggggcggtaccATGTCTTGTCTTGATTTGACCTTCT-3'	5'-ccatttgttggatccTCAATTATCGCTGTGGGGTAT-3'
GL3-Cluc	5'-tcccggggcggtaccATGGCTACTGGGCTCCAAAAC-3'	5'-ccatttgttggatccTCAACTGTTCCAAGCAATTCTCTG-3'
TTG1-Cluc	5'-tcccggggcggtaccATGGAGAACACGACACAAGAATC-3'	5'-ccatttgttggatccTCAAACTTTCAAAAGCTGCATTT-3'
**Primers for transient expression studies in tobacco.**		
bHLH3P-GUS	*5'-CGCGGATCC*CATGTTGTGTGGACCTTTTTATGTCC-3'	*5'-CATGCCATGG*TCTCACAAGCTGATCAGAGTTTCC-3'
MYB4P-GUS	*5'-CGCGGATCC*GCTTGAATTACTTTTGTGCGTGTG-3'	*5'-CATGCCATGG*TTCTCGCTTCTCACAGCAAGG-3'
**Primers for subcellular localization (GFP) assays**		
MYBA-PZYGC	5'-agaggacagggtaccATGCAAACGGAGGGCTGTTC-3'	5'-gacactagtggatccCTGCATTTCAGCATTTCCT-3'
TT2L1-PZYGC	5'-agaggacagggtaccATGGGAAGAAACCCTTGTTGC-3'	5'-gacactagtggatccATCAGTCAACCAGTTTTCTTCATCG-3'
TT2L2-PZYGC	5'-agaggacagggtaccATGGGAAGAAAACCTTGTTGTTCAA-3'	5'-gacactagtggatccAACTTGAAGCCACCTATCGAAG-3'
TT2L3-PZYGC	5'-agaggacagggtaccATGGGAGAGAGACGTCCTCG-3'	5'-gacactagtggatccATATTGTTGATCAGGAAAGGT-3'
bHLH3-PZYGC	5'-agaggacagggtaccATGTCTTGTCTTGATTTGACCTTCT-3'	5'-gacactagtggatccATTATCGCTGTGGGGTAT-3'
GL3-PZYGC	5'-agaggacagggtaccATGGCTACTGGGCTCCAAAAC-3'	5'-gacactagtggatccACTGTTCCAAGCAATTCTCTG-3'
TTG1-PZYGC	5'-agaggacagggtaccATGGAGAACACGACACAAGAATC-3'	5'-gacactagtggatccAACTTTCAAAAGCTGCATTT-3'
MYBF-PZYGC	5'-agaggacagggtaccATGGGAAGAGCTCCGTGCT-3'	5'-gacactagtggatccGGAGAGAAGCCAAGCGA-3'
MYB4-PZYGC	5'-agaggacagggtaccATGAACAAAGGAGCTTGGTCAA-3'	5'-gacactagtggatccTGTGAAGAGATTTTGAAGCGT-3'
**Primers for transformation of tobacco and Arabidopsis.**		
MYBA-PLGNL	5'-agaggacagggtaccATGCAAACGGAGGGCTGTTC-3'	5'-gacactagtggatccCTACTGCATTTCAGCATTTCCT-3'
TT2L1-PLGNL	5'-agaggacagggtaccATGGGAAGAAACCCTTGTTGC-3'	5'-gacactagtggatccTTAATCAGTCAACCAGTTTTCTTCATCG-3'
TT2L2-PLGNL	5'-agaggacagggtaccATGGGAAGAAAACCTTGTTGTTCAA-3'	5'-gacactagtggatccTTAAACTTGAAGCCACCTATCGAAG-3'
bHLH3-PLGNL	5'-agaggacagggtaccATGTCTTGTCTTGATTTGACCTTCT-3'	5'-gacactagtggatccTCAATTATCGCTGTGGGGTAT-3'
GL3-PLGNL	5'-agaggacagggtaccATGGCTACTGGGCTCCAAAAC-3'	5'-gacactagtggatccTCAACTGTTCCAAGCAATTCTCTG-3'
TTG1-PLGNL	5'-agaggacagggtaccATGGAGAACACGACACAAGAATC-3'	5'-gacactagtggatccTCAAACTTTCAAAAGCTGCATTT-3'
MYBF-PLGNL	5'-agaggacagggtaccATGGGAAGAGCTCCGTGCT-3'	5'-gacactagtggatccTCAGGAGAGAAGCCAAGCGA-3'
MYB4-PLGNL	5'-agaggacagggtaccATGAACAAAGGAGCTTGGTCAA-3'	5'-gacactagtggatccTCATGTGAAGAGATTTTGAAGCGT-3'
**Primers for qRT-PCR and semi quantitative RT-PCR assays.**		
CHS2	5'-ATCATGGCAATCGGGACG-3'	5'-TGCTCACTGTTAGTAATACGGAAA-3'
CHI	5'-GGGAAGTCGGTGGAGGAA-3'	5'-CGTCTGCGTCAGTGTAAATC-3'
F3H	5'-CGCCGTGCTGAGATTTG-3'	5'-CTAACCGCTTCACCCTGTA-3'
FNS	5'-CCCTCACCATCCTGCTC-3'	5'-GCTTTCGGCGGGCTTAT-3'
CYP75B1	5'-AATCAACGGCTACCACATCC-3'	5'-TCATACCCGCACAAATCCTAC-3'
CYP75B2	5'-TATCAATGGCTACCACATACCTAA-3'	5'-CCGACCCAAATGGAATCAC-3'
FLS1	5'-TTCCCGAGATACCCACCGTC-3'	5'-TTGGCGTAAAACTCTTTCTCCT-3'
FLS3	5'-CATCCCTACCGAGGTCATACG-3'	5'-TTCTTTCCATTCACGATCTTTTG-3'
DFR	5'-TTGTCGGACCGTAAAGATG-3'	5'-GCAGTGGGACCAAGAAAT-3'
LAR	5'-TTTGGGCACGACGTAGAC-3'	5'-GCCATCACCGTAGATTTTG-3'
ANS	5'-GCTTGCGACCACCATACTTT-3'	5'-TGCCGTCACCCATTTGC-3'
ANR	5'-GTGTCAAAGCGAAAACGG-3'	5'-CACAGGGTAGCCCCAAGTA-3'
UFGT	5'-GAGACGGGGAGGGAGGTTAG-3'	5'-GGGTTCGTTTTCACTTCCTTTAG-3'
MYBA	5'-GAAGCCGGATGTCAAAAG-3'	5'-TCCTACCCGCAATCAACG-3'
TT2L1	5'-GAATTACTGGAACACCAATTTAGG-3'	5'-TCTTATTGGACGGTGATGCC-3'
TT2L2	5'-TCCTTGGCAACAGATGGT-3'	5'-TGTTTCTTGGTTGGCTTTG-3'
TT2L3	5'-CCGATGAAGGTTCGAGTAGG-3'	5'-CTCCCAGATTTCTTGAGTCCC-3'
bHLH3	5'-TCCCAGTGGCTCTTAAAATACATC-3'	5'-AAGGCACAAGCGACCTCAAT-3'
GL3	5'-ACGGGATCATTTCCTTAATTATTCA-3'	5'-GTGAGAAAATAACAAGCTTCAACTGT-3'
TTG1	5'-GGCTCCGCAGAGTTACAGAC-3'	5'-AAAGGCGATGGCGATCCAAT-3'
MYBF	5'-TGTGAGAAAGTTGGGCTGAAG-3'	5'-CGGCATTCTTGGGTAGTGAT-3'
MYB4	5'-GGTCACGAGGTTTCAGATGGC-3'	5'-TTTTCCCGATTTGGGCTTTA-3'
MaActin	5'-GCATGAAGATCAAGGTGGTG-3'	5'-CATCTGCTGGAAGGTGCTAA-3'
NtActin	5'-TCACAGAAGCTCCTCCTAATCCA-3'	5'-GAGGGAAAGAACAGCCTGAATG-3'
AtActin	5'-GCTCCTCTTAACCCAAAGGC-3'	5'-CACACCATCACCAGAATCCAGC-3'

**Primers for transgene detection**		
MYB4P-GUS-check	5’-TCTTTAGCCTAAGTTTGCTGT-3’	5’-ACGCTGAGTAATTCGTTGAT-3’

The underline represents the restriction site;

Lower case letters: gene-specific primers with 15 bp extensions homologous to the ends of the linearized vector for In-Fusion cloning technology.

Supplemental Table S9. Accession numbers of sequences used in this study.

Species	Sequence Name	Accession numbers
Antirrhinum majus	AmDEL	AAA32663
Arabidopsis thaliana	AtCOP1	NP_180854.1
	AtEGL3	Q9CAD0
	AtGL3	NP_680372
	AtMYB11	NP_191820.1
	AtMYB111	NP_199744.1
	AtMYB113	NP_176811.1
	AtMYB12	NP_182268.1
	AtMYB3	NP_564176.2
	AtMYB4	NP_195574.1
	AtMYC1	Q8W2F1
	AtPAP1	NP_176057.1
	AtPAP2	NP_176813.1
	AtTT2	NP_198405.1
	AtTT8	Q9FT81
	AtTTG1	NP_001318637.1
Brassica napus	BnTTG1	XP_013643681.1
Brassica oleracea var. botrytis	BoTT8	ADP76654
	BoTTG1	XP_013599438.1
Brassica rapa	BrCOP1	VDC70492.1
	BrTTG1	XP_009150977.1
Cajanus cajan	CcCOP1	XP_020233903.1
Diospyros kaki	DkMYB2	AB503699.1
Epimedium sagittatum	EsMYBA1	KC335202
Fragaria x ananassa	FabHLH3	AFL02463
	FaMYB1	AF401220_1
	FaMYB11	AFL02461.1
Fagopyrum tataricum	FtMYB15;	KX758436
Fragaria vesca subsp. vesca	FvEGL1	XP_004308377
Gossypium hirsutum	GhCOP1L	XP_016696389.1
Garcinia mangostana	GmMYB10	ACM62751.1
Gerbera hybrid cv. 'Terra Regina'	GMYB10	AJ554700
Gentiana triflora	GtMYBP3	AB733016
	GtMYBP4	AB289446
Hordeum vulgare subsp. Vulgare	HvMYB10	AB645844
Ipomoea nil	InDEL	BAE94393
	InIVS	BAE94394
Ipomoea purpurea	InMYB1	AB232769
Lilium hybrid division I	LhbHLH1	BAE20057
	LhbHLH2	BAE20058
Lotus japonicus	LiTT8	AB490778
	LjGL3	AB492284
	LjTT2a	BAG12893.1
	LjTT2b	BAG12894
	LjTT2c	BAG12895
Malus domestica	MdbHLH3	ADL36597
	MdbHLH33	ABB84474
	MdMYB3	JN544704
	MdMYBA	AB279598
Morella rubra	MrMYB1	GQ340767
Medicago truncatula	MtMYB2	XM_003616340
Perilla frutescens	MYC-RP	AB024050
Capsella rubella	CrCOP1	XM_023784273.1
Nicotiana tabacum	NtAN1a	HQ589208
	NtAN1b	HQ589209
	NtJAF13	KF298397
Pyrus communis	PcMYB10	EU153575
Perilla frutescens	PfF3G1	AB103172
Petunia x hybrida	PhAN1	AAG25928
	PhAN11	AAC18914.1
	PhAN2	AAF66727.1
	PhJAF13	AAC39455
	PhMYB27	AHX24372
	PhMYB4	ADX33331.1
Prunus persica	PpMYB10	EU155160
	PpMYB18	KT159234
Populus trichocarpa	PtMYB134	FJ573151
	PtMYB165	XP_002315890.2
	PtMYB182	XP_002305872.1

Ricinus communis	RcCOP1	XP_002534127.1
Raphanus sativus	RsTT8	KY651179
	RsTTG1	XP_018484504.1
Solanum tuberosum	StAN1	JX848659
	StMTF1	EU310399
Trifolium repens	Tr-MYB134	KT699109
	Tr-MYB7	KT699107
Vitis labrusca x Vitis vinifera	VlMYBA1-1	AB073010
Vitis vinifera	VvMYB4a	ABL61515.1
	VvMYB4b	ACN94269.1
	VvMYBC1	ACC68685
	VvMYBC2-L1	AFX64995.1
	VvMYBC2-L2	ACX50288.2
	VvMYBC2-L3	AIP98385.1
	VvMYBF1	GQ423422
	VvMYBPA2	EU919682
	VvMYCA1	ABM92332
Zea mays	ZmB	CAA40544
	ZmIN1	AAB03841
	ZmLC	P13526
	ZmMYB-IF35	AF521880
	ZmPAC1	NP_001310302.1
Morus alba L. cv. 'hongguo2'	bHLH3	MN337861
	GL3	MN337862
	MYB4	MN337865
	MYBA	MN337857
	MYBF	MN337864
	TT2L1	MN337859
	TT2L2	MN337858
	TT2L3	MN337860
	TTG1	MN337863
Morus notabilis C.K. Schneid	Morus24997	EXB39302.1
	Morus25446	EXC01078.1
	CYP75B1	EXC06425.1
	CYP75B2	EXC06424.1
Allium cepa	F3'H	AY541035.1
Arabidopsis thaliana	F3'H	AF271651.1
Callistephus chinensis	F3'5'H	AF313489
	F3'H	FJ216428.1
Campanula medium	F3'5'H	D14590.1
Coptis japonica	CYP80B2	AB025030.1
Delphinium grandiflorum	F3'5'H	AY856345.1
Eschscholzia californica	CYP80B1	AF014801.1
Gentiana triflora	F3'5'H	D85184.1
	F3'H	AB193313.1
Gerbera hybrida	F3'H	DQ218417
Glycine max	F3'H	AF499731.1
	F3'5'H	AY117551.1
Gossypium hirsutum	F3'5'H	AY275430.1
Hieracium pilosella	F3'H	DQ319866
Ipomoea nil	F3'H	AB113264.1
Lycianthes rantonneti	F3'5'H	AF313490.1
Matthiola incana	F3'H	AF313491.1
Nierembergia sp.	F3'5'H	AB078514.1
Osteospermum hybrida	F3'5'H	KP899545
	F3'H	DQ250711
Oryza sativa	F3'H	XP_015613041.1
	F3'Hb	AK070442
Pelargonium hortorum	F3'H	AF315465.1
Pericallis cruenta	F3'5'H	AY791885
Perilla frutescens	F3'H	AB045593.1
Petunia x hybrida	F3'H	AF155332.1
	F3'5'H	AY245545.1
Solanum melongena	F3'5'H	X70824.1
Solanum tuberosum	F3'5'H	AY675559.1
Torenia hybrida	F3'5'H	AB012925.1
	F3'H	AB057673.1
Vinca major	F3'5'H	AB078781.1

Supplemental Table S10. Transcription factors correlated with *bHLH3* expression in connection network

ID in MorusDB	Family	Symbol	Description	Correlation
L484_012139	bZIP	BZIP61	Transcription factor RF2b [Morus notabilis]	Positive correlation
L484_020313	MYB_related	-	PREDICTED: protein RADIALIS-like 4 [Theobroma cacao]	Positive correlation
L484_022975	MYB	-	MYB4 [Morus notabilis]	Positive correlation
L484_020177	ERF	ERF012	Ethylene-responsive transcription factor [Morus notabilis]	Positive correlation
L484_019376	bHLH	ORG2	Transcription factor ORG2 [Morus notabilis]	Positive correlation
L484_013609	ZF-HD	-	Mini zinc finger 2 isoform 2, partial [Theobroma cacao]	Positive correlation
L484_014404	S1Fa-like	S1FA1	DNA-binding protein S1FA1 [Morus notabilis]	Positive correlation
L484_016991	C2H2	-	Transcriptional regulator TAC1 [Morus notabilis]	Positive correlation
L484_001828	Dof	DOF1.2	Dof zinc finger protein [Morus notabilis]	Positive correlation
L484_003860	WRKY	WRKY44	WRKY transcription factor 44 [Morus notabilis]	Positive correlation
L484_026215	HD-ZIP	GL2	Homeobox-leucine zipper protein GLABRA 2 [Morus notabilis]	Positive correlation
L484_000097	MYB	-	Transcription factor [Morus notabilis]	Positive correlation
L484_004072	TALE	-	Homeobox protein [Morus notabilis]	Positive correlation
L484_009669	DBB	-	Zinc finger protein CONSTANS-LIKE 2 [Morus notabilis]	Negative correlation
L484_023484	LBD	LBD11	lateral organ boundaries domain protein [Boehmeria nivea]	Positive correlation
L484_012905	MYB_related	ETC1	PREDICTED: MYB-like transcription factor ETC1 [Eucalyptus grandis]	Positive correlation
L484_007959	MIKC	-	Floral homeotic protein DEFICIENS [Morus notabilis]	Positive correlation
L484_017595	MYB_related	-	TRIPTYCHO (TRY) [Arabidopsis thaliana]	Positive correlation
L484_001405	M-type	-	PREDICTED: transcription factor of morphogenesis MCM1-like [Ziziphus jujuba]	Positive correlation
L484_004404	AP2	-	AP2-like ethylene-responsive transcription factor [Morus notabilis]	Positive correlation
L484_006971	B3	-	PREDICTED: auxin response factor 16-like [Citrus sinensis]	Positive correlation
L484_020870	YABBY	-	YABBY protein [Corchorus olitorius]	Positive correlation
L484_018706	MYB	-	Transcription factor [Morus notabilis]	Positive correlation
L484_027360	MYB	C1	Anthocyanin regulatory C1 protein [Morus notabilis]	Positive correlation
L484_015439	bHLH	BHLH75	PREDICTED: transcription factor bHLH75-like isoform X4 [Prunus mume]	Positive correlation
L484_016584	B3	-	B3 domain-containing transcription factor NGA4 [Morus notabilis]	Positive correlation
L484_021618	ERF	-	Ethylene-responsive transcription factor [Morus notabilis]	Positive correlation
L484_022665	MYB	MYB86	Transcription factor [Morus notabilis]	Positive correlation
L484_012134	NAC	-	NAC domain-containing protein 29 [Morus notabilis]	Positive correlation
L484_019377	bHLH	-	Transcription factor ORG2 [Morus notabilis]	Positive correlation
L484_019377	bHLH	-	Transcription factor ORG2 [Morus notabilis]	Positive correlation
L484_026077	bHLH	-	PREDICTED: transcription factor bHLH96-like [Glycine max]	Positive correlation
L484_006054	NAC	NAC072	NAC domain-containing protein 72 [Morus notabilis]	Negative correlation
L484_006652	C2H2	-	Zinc finger protein ZAT4 [Morus notabilis]	Positive correlation
L484_012750	HD-ZIP	-	Homeobox-leucine zipper protein ATHB-16 [Morus notabilis]	Positive correlation
L484_008831	NAC	NAC083	NAC domain-containing protein 29 [Morus notabilis]	Positive correlation
